# Investigation of the sensitivity of functional near-infrared spectroscopy brain imaging to anatomical variations in 5- to 11-year-old children

**DOI:** 10.1117/1.NPh.5.1.011009

**Published:** 2017-09-18

**Authors:** Ashley C. Whiteman, Hendrik Santosa, Daniel F. Chen, Susan Perlman, Theodore Huppert

**Affiliations:** a University of Pittsburgh, Department of Radiology, Pittsburgh, Pennsylvania, United States; b University of Pittsburgh, Department of Psychiatry, Pittsburgh, Pennsylvania, United States; c University of Pittsburgh, Department of Bioengineering, Pittsburgh, Pennsylvania, United States

**Keywords:** near-infrared spectroscopy, anatomical variability, structural anatomy, Monte Carlo simulation, radiative transport equation

## Abstract

Functional near-infrared spectroscopy (fNIRS) is a noninvasive brain imaging technique that uses scalp-placed light sensors to measure evoked changes in cerebral blood oxygenation. The portability, low overhead cost, and ability to use this technology under a wide range of experimental environments make fNIRS well-suited for studies involving infants and children. However, since fNIRS does not directly provide anatomical or structural information, these measurements may be sensitive to individual or group level differences associated with variations in head size, depth of the brain from the scalp, or other anatomical factors affecting the penetration of light into the head. This information is generally not available in pediatric populations, which are often the target of study for fNIRS. Anatomical magnetic resonance imaging information from 90 school-age children (5 to 11 years old) was used to quantify the expected effect on fNIRS measures of variations in cerebral and extracerebral structure. Monte Carlo simulations of light transport in tissue were used to estimate differential and partial optical pathlengths at 690, 780, 808, 830, and 850 nm and their variations with age, sex, and head size. This work provides look-up tables of these values and general guidance for future investigations using fNIRS sans anatomical information in this child population.

## 1 Introduction

Functional near-infrared spectroscopy (fNIRS) is a noninvasive neuroimaging technique that uses low levels of red to near-infrared light (650 to 950 nm) to measure changes in the optical absorption of tissue due to hemoglobin. In this region of wavelengths, often referred to as the “optical window,” light can propagate up to several centimeters through tissue, which is deep enough to reach parts of the cerebral cortex from optical emitters and detectors typically placed on the surface of the scalp. Using a grid of these sensors placed within a head cap and worn by the participant, the underlying changes in evoked cerebral hemodynamic responses can be spatially and temporally recorded. The precise penetration of light into the brain, however, depends on a number of individual factors such as optical scattering and the anatomical structure of the brain and extracerebral layers. Variations between individuals in the cortical folding of the brain, head-size, skull thickness, or layers of cerebral spinal fluid (CSF) can influence the recorded fNIRS signals and the sensitivity to underlying brain activity. This is a limiting situation since one of the advantages of fNIRS technology is often cited as its portability, low cost, and ability to record brain activity from pediatric or other special subject populations for whom magnetic resonance imaging (MRI) may be difficult or contraindicated. The objective of this current work is to investigate how these factors affect fNIRS measurements in school-age children and to systematically examine the quantitative effect of age, head-size, and sex on fNIRS measurements. Specifically, we examine the effects on the optical differential pathlength factor (DPF) and partial pathlength factor (PPF) using Monte Carlo modeling of the optical transport model. A dataset of structural MRI volumes from 90 children (58 to 131 months) is examined in this work.

### 1.1 Functional Near-Infrared Spectroscopy Imaging

Over the last 40 years, since it was first demonstrated by Jobsis,^1^ fNIRS has been applied to a growing number of applications in psychology, psychiatry, and brain development.^2^^–^^6^ In particular, the application of fNIRS in child and infant populations has been successfully demonstrated by numerous researchers.^7^^–^^12^ Compared to functional magnetic resonance imaging (fMRI), fNIRS recordings can be made in a nonrestrictive environment and do not require a specialized scanning room nor the participant to lie in a motionless supine position. During fNIRS imaging, the participant can sit or even stand while wearing the fNIRS head cap, allowing reasonable movement of the participant and interactions with other people or the environment.

While a number of researchers have used fNIRS to examine group-level changes in brain activity with respect to subject age or sex in the context of child development, a persistent underlying confound of such work is the potential for systematic differences in the underlying structure of the head, brain, and other factors that affect the magnitude of the fNIRS signal. While light in the near-infrared window can penetrate up to several centimeters of tissue due to low intrinsic absorption in this range, light passing through the tissue is highly scattered resulting in the diffusion of the light through the tissue. Thus, the path of this scattering depends on the structure of the head, particularly the boundaries of the brain, skull, and CSF. In these layers, systematic differences with age or sex would result in a bias in the reported magnitude of the fNIRS recordings. For instance, in older adults, atrophy of the frontal and temporal cortices^13^ could result in a decrease in the sensitivity and reported magnitude of measured brain activation as the distance increases between the brain and the surface of the scalp where the fNIRS sensors are positioned.^14^ Similarly, in children, the growth of the head and/or brain could cause similar biases. Previous work by Beauchamp et al.^15^ examined changes in the structure of the brain over a range of pediatric structural MRI volumes in 71 children ages 0 to 12 year old. While this work documented the changes in the scalp-brain distance with age, the direct quantitative impact on fNIRS was not examined. In particular, these changes would have an effect on the optical path of light in tissue and the fraction of the signal coming from the actual brain compared to the superficial layers.

### 1.2 Modified Beer–Lambert Law

Cope et al.^16^ introduced the concept of the modified Beer–Lambert law (MBLL) as a way to approximate the effect of scattering on the propagation of light in tissue and the resulting increase in the effective distance (optical pathlength) that light travels as it moves through the tissue. The modified Beer–Lambert relationship is given by

$${\Delta \mathrm{OD}}^{\lambda }≅(\underset{i}{\sum }\epsilon_{i}^{\lambda }\cdot c_{i})\cdot L\cdot {\mathrm{DPF}}^{\lambda }\cdot {\mathrm{PVF}}^{\lambda },$$ (1)

where ΔOD is the change in optical density (absorption) measured between an fNIRS source-to-detector pair, ϵ is the extinction coefficient at a particular wavelength (λ) and for a particular i’th chromophore, and $c_{i}$ is the concentration of that chromophore. In the case of fNIRS, the two chromophores of interest are typically oxy- and deoxy-hemoglobin (${\mathrm{HbO}}_{2}$ and Hb, respectively). In the original Beer–Lambert law, optical density is proportional to the optical pathlength through the sample. However, in the MBLL, this is replaced by an effective pathlength to account for scattering of the light and the diffuse path that photons will travel in the tissue. The effective pathlength through the tissue is approximated by the product of the distance along the surface between a source–detector pair (L) and a wavelength correction term called the DPF, which is a unitless scalar that adjusts for scattering. As an example, for a scalp distance (L) of 3 cm between an fNIRS source–detector pair, the photon will typically travel an effective distance of ∼18  cm as it scatters back and forth though the tissue. In this case, the DPF would be 6 (18  cm=3  cm×6). However, of this 18 cm, most of this is through the extracerebral skin, skull, and CSF layers that are of little interest to fNIRS. Thus, the partial volume factor (PVF) in Eq. (1) is applied to adjust for the fraction of this path that is actually in the brain. The resulting correction to the MBLL ($L\cdot {\mathrm{DPF}}^{\lambda }\cdot {\mathrm{PVF}}^{\lambda }$) represents the effective pathlength through the brain volume of interest specifically. The PPF is defined as

$${\mathrm{PPF}}^{\lambda }={\mathrm{DPF}}^{\lambda }\cdot {\mathrm{PVF}}^{\lambda },$$ (2)

giving the brain-specific MBLL as

$${\Delta \mathrm{OD}}^{\lambda }≅(\underset{i}{\sum }\epsilon_{i}^{\lambda }\cdot c_{i})\cdot L\cdot {\mathrm{PPF}}^{\lambda }.$$ (3)

Thus, PPF is a wavelength specific, unitless scalar that adjusts for the effective pathlength in the brain. In contrast, DPF adjusts for the pathlength through all tissues. Since the optical measurements between a source-to-detector pair (ΔOD) are proportional to PPF and the magnitude of the signal change in the brain, the PPF term is directly relevant in examining systemic differences with age, sex, or head size.

## 2 Methods

### 2.1 Subject Population

All MRI data were collected at 3 Tesla on a Siemens TIM TRIO scanner using T1-wieghted MPRAGE imaging. All subjects had participated in one of the several imaging studies between 2012 and 2015 as part of the healthy/control cohort at the University of Pittsburgh and provided written IRB consent via parent/guardian proxy.^17^ Structural MR images from a total of 95 subjects were used in this work. Five of the subjects were removed from analysis due to low MRI quality and/or errors in the anatomical registration or segmentation algorithms. Of the 90 remaining subjects, 46 were female. The age range was 58 to 131 months (mean 95 months; SD 17 months). The demographics of these subjects are provided in Table 1.

**Table 1:** Subject demographics.

	Males (n=46)				Females (n=44)			
	Mean	StdDev	Min	Max	Mean	StdDev	Min	Max
Age (months)	96.1	15.9	60.9	124.2	92.3	17.7	58.1	131.4
Weight (Kg)	28.9	7.3	15.9	46.3	30.6	10.7	15.9	68.0
Height (m)	1.29	0.10	1.02	1.50	1.26	0.14	0.91	1.52
Head circumference (cm)	38.9	2.4	33.7	44.5	38.7	2.0	34.0	42.6
Arc length AP (cm)	37.1	1.1	34.5	39.0	37.3	1.3	34.5	40.2
Arc length RL (cm)	34.5	0.9	32.2	36.1	34.7	1.2	32.2	37.4

Note: 3-T MRI data from a total of 90 subjects was used in this study. Age (months), height, and weight were taken at the time of scan. Head circumference and the arc length in the anterior–posterior (AP; nasion to inion) and right–left (between preauricular points) directions were computed *post hoc* from the structural MRI data as described in the text.

### 2.2 Magnetic Resonance Imaging Processing and Segmentation

The structural T1-weighted MR images were processed through the FreeSurfer-based^18^ HCP structural pipelines.^19^ This automated processing pipeline is designed to produce minimally distorted structural volumes for each subject both in “native” space and standardized Montreal Neurological Institute (MNI) space.

T1-weighted images were internally cropped to a smaller field of view to remove the neck using FSL’s “robustfov” tool, then aligned to the MNI template space using a 12 degree-of-freedom affine FMRIB's linear image registration tool. A brain mask was applied and then a 6-degree-of-freedom transform was used to align the anterior commissure (AC), posterior commissure (PC), and AC-PC line. The AC-PC aligned brain extracted images were then registered linearly and nonlinearly to the MNI template. These warps were inverted and the template brain masks were brought back into the AC-PC aligned space.

The aligned T1-weighted images were then intensity normalized and FreeSurfer’s “recon-all” function was run to generate white matter and pial surfaces. Pial surfaces were generated using Gaussian parameters (3 standard deviations above and below gray matter mean intensity). Morphometric measurements of volumes, surface areas, and thicknesses were then computed from these surfaces.

A secondary segmentation was performed on the skin, skull, and CSF layers using the FreeSurfer watershed algorithm “mri_watershed.”^20^ The watershed segmentation algorithm was used to determine the intensity values for white matter, gray matter, and CSF. An elliptical surface was fitted to the brain and the shape of the surface fit was evaluated against a previously derived template. The brain surface file was then grown outward to generate an inner skull surface. A fifth-order icosahedral surface was fit around the outer edge of the volume and smoothed to make the skin surface. Finally, this skin surface was shrunk to make the outer skull surface. Following automated segmentation using the FreeSurfer and “mri_watershed” algorithms, the brain head-layer surfaces were visually examined. A total of five subjects were discarded due to either poor segmentation, registration, or general data quality leaving 90 final datasets that were used in analysis.

### 2.3 Functional Near-Infrared Spectroscopy Modeling

Each of the 90 subjects’ segmented anatomical volumes was used to model the sensitivity and characteristics of theoretical fNIRS measurements using Monte Carlo simulations.^21^^,^^22^ A four-layer (skin, skull, CSF, and brain) anatomical mesh-based model was created from the segmented boundaries of the skin, outer skull, inner skull, and pial surface of the brain using the iso2mesh program from Fang and Boas.^23^ Monte Carlo simulations were run at five wavelengths (690, 780, 808, 830, and 850 nm). The optical properties for these tissues and wavelengths are given in Table 2. In this work, gray and white matter brain tissues were assigned the same optical properties similar to the earlier work in Strangman et. al.^24^

**Table 2:** Tissue optical properties for Monte Carlo simulations.

		Wavelength (nm)				
		690	780	808	830	850
Skin	$\mu_{A}$ (${\mathrm{mm}}^{-1}$)	0.021	0.014	0.012	0.012	0.012
	$\mu_{S}^{\prime }$ (${\mathrm{mm}}^{-1}$)	2.91	2.45	2.33	2.24	2.16
	N	1.45	1.45	1.45	1.45	1.45
	g	0.89	0.89	0.89	0.89	0.89
Skull	$\mu_{A}$ (${\mathrm{mm}}^{-1}$)	0.026	0.025	0.025	0.025	0.027
	$\mu_{S}^{\prime }$ (${\mathrm{mm}}^{-1}$)	1.82	1.67	1.62	1.59	1.57
	N	1.45	1.45	1.45	1.45	1.45
	g	0.89	0.89	0.89	0.89	0.89
CSF	$\mu_{A}$ (${\mathrm{mm}}^{-1}$)	0.001	0.002	0.002	0.003	0.004
	$\mu_{S}^{\prime }$ (${\mathrm{mm}}^{-1}$)	0.01	0.01	0.01	0.01	0.01
	N	1.33	1.33	1.33	1.33	1.33
	g	0.89	0.89	0.89	0.89	0.89
Brain (gray + white)	$\mu_{A}$ (${\mathrm{mm}}^{-1}$)	0.010	0.011	0.011	0.012	0.014
	$\mu_{S}^{\prime }$ (${\mathrm{mm}}^{-1}$)	1.44	1.18	1.12	1.07	1.03
	N	1.45	1.45	1.45	1.45	1.45
	g	0.89	0.89	0.89	0.89	0.89

Note: This table provides the optical properties for the skin, skull (bone), CSF, and brain layers used in the Monte Carlo simulations. Optical properties were adopted from Jacques.^19^ $\mu_{A}$ is the optical absorption coefficient, $\mu_{s}^{\prime }$ is the reduced scattering coefficient, N is the index of refraction, and g is the anisotropy coefficient.

The optical properties were computed from the general tissue models given by Jacques^25^ and optical scattering ($\mu_{s}^{\prime }$) and absorption ($\mu_{A}$) was computed by

$$\mu_{s}^{\prime }=a\cdot {\left[\frac{\lambda }{500(\mathrm{nm})}\right]}^{-b},$$ (4)

$$\mu_{A}=B\cdot S\cdot \mu_{A,\mathrm{HbO}2}+B\cdot (1-S)\cdot \mu_{A,\mathrm{Hb}}+W\cdot \mu_{A,\text{water}}+F\cdot \mu_{A,\text{fat}},$$ (5)

where the parameters for these two equations were compiled from various empirical sources in the literature as given in Jacques.^25^ For scattering, the parameters for the skin ($a=4.6\;{\mathrm{mm}}^{-1}$, b=1.421), bone ($a=2.29\;{\mathrm{mm}}^{-1}$, b=0.716), and brain ($a=2.42\;{\mathrm{mm}}^{-1}$, b=1.611) were used. For the brain, we assumed oxygen saturation (S)=70%, blood volume fraction (B)=2.2% [=50  μM×(66,458  gm/mol)/(150  gm Hb/L blood)], water fraction (W)=70%, and fat fraction (F)=0%. For the other tissues, we used values given by Jacques.^25^

**Fig. 1:**
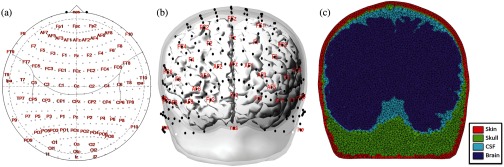
Example of segmentation and Monte Carlo simulations. (a) and (b) The locations of the 10-5 coordinate points on the surface of one of the subjects and the equivalent polar projection of this map. (c) An example mesh used for the Monte Carlo simulations in the same subject.

For each subject, we modeled the optical sensitivity (forward model) using a mesh-based Monte Carlo method.^22^ Functional NIRS sources were modeled from 346 positions on the head for each subject at the international 10-5 coordinate positions (see Fig. 1). For each source and wavelength, $5\times 10^{7}$ photons were simulated. Thus, over the whole cohort, 157,000 simulations were run for a total of 72,000 CPU-hours of computing on a 240 CPU high-performance computing cluster. For each simulation, the exiting photons were monitored at all other positions on the surface of the head yielding a 346×2564 matrix of virtual fNIRS source–detector combinations from around each source position.

For each simulated source–detector pair, the optical pathlength through the entire tissue (DPF) and partial pathlength through the brain (PPF) was recorded. The DPF and PPF were computed for each 10-5 position by fitting the ∼120 measurements between 10 and 40 mm around each source to a linear regression model using an iterative robust regression algorithm (MATLAB function “robustfit”).

### 2.4 Topographic Projections

Each subject was registered to a 10-5 head coordinate space by aligning the left/right preauricular ear points and nasion point in the MRI data to an elliptical template of 10-5 points included in the SPM software (“ext1020.sfp”)^26^ followed by an iterative closest point registration and refinement using the head surface. Based on this head coordinate registration, the head circumference (at 10% up along the arc length between preauricular ear points) and the arc lengths from nasion to inion and between preauricular ear points was computed retrospectively.

To compare estimated parameters (cortical depth, optical pathlength, etc.) across subjects, values of interest were first projected along the surface normal to the nearest position on the scalp. The scalp positions were then projected using a Clarke’s twilight azimuthal projection using a normalized head radius into a two-dimensional topographic map and interpolated onto an equidistant polar grid. This projection allowed data from subjects to be compared in a standardized space independent of the subject head circumference and size. Except where noted, the median value of the parameters was computed across subjects in this polar (10-5 coordinate) space.

## 3 Results

### 3.1 Characterization of Anatomical Variations

**Fig. 2:**
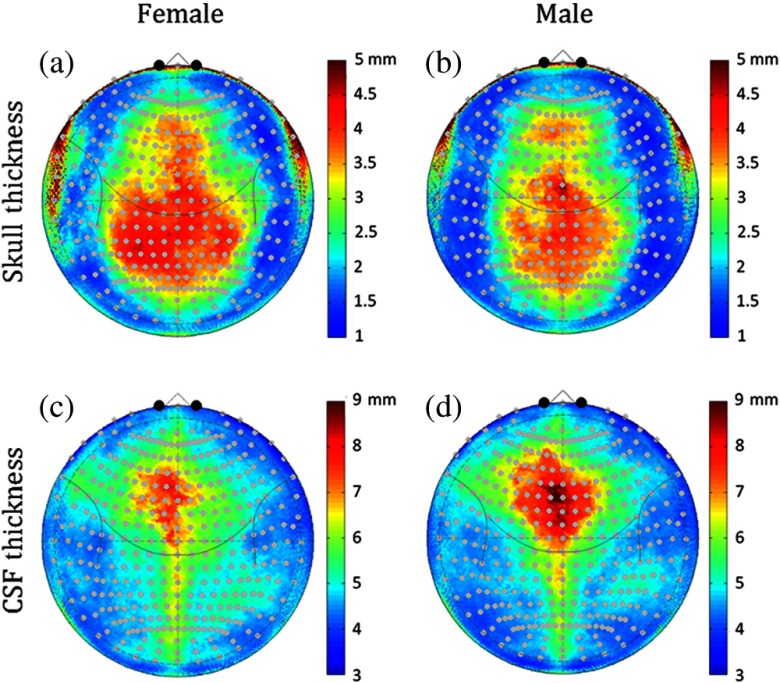
Gender differences in skull CSF layer thickness. (a) and (b) The median skull and (c) and (d) CSF thickness for the male and female subjects is shown in the normalized polar (10-5 coordinate) space. The color scales show the thickness of these layers in millimeters.

Using the anatomical MRI volumes from the 90 children, we computed the median skull thickness and CSF layer thickness according the 10-5 space projected values. The median value for the male and female groups is shown in Fig. 2. The skull thickness ranged between 1.1 and 9.6 mm {2.51±0.92 [median±0.67499×(median absolute deviation)]} in the females and 1.1 to 8.3 mm (2.66±0.92) in the males. In both sexes, the skull thickness at the crown and posterior of the head (posterior of 10-5 position FpZ and lateral out to positions Cp3/Cp4) was about 2 to 3 times thicker (3.2 to 5 mm) than the other frontal or temporal regions as shown in Fig. 2.

The CSF thickness (defined as the distance between the inner surface of the skull and the pial surface of the brain) was between 3 and 9 mm in both sexes (male 5.12±0.95; female 5.05±0.89) and greatest (7 to 9 mm) at the slightly anterior to the crown of the head around 10-5 position Fz and extending posterior along the sagittal sulcus. The area of this thicker CSF region was larger in the males than the females. In our segmentations, we did not consider the venous dural sinuses, and some of this CSF thickness at the sagittal sulcus is likely due to the sagittal sinus, which is labeled as CSF in the segmentations.

**Fig. 3:**
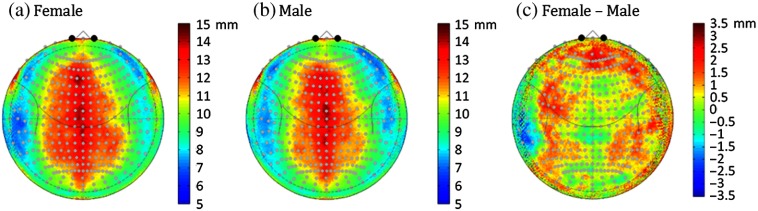
Gender differences in the scalp-to-brain depth. (a) and (b) The median distance normal to the surface from the scalp to the nearest position on the pial cortical surface in the subject normalized polar (10-5 coordinate) space. (c) The difference between the depth of the females compared to the males. In most regions, the brain of the female subjects up to 3-mm deeper.

The depth of the surface of the cortex relative to the scalp is shown in Figs. 3(a) and 3(b) for both sexes. The cortical depth ranged from 6.2 to 14.7 mm in females (median 10.2±1.8) and 6.6 to 14.5 mm in males (median 10.13±1.73). This was lowest (7 to 9 mm) along the frontal cortex and bilateral lateral regions. This depth was about 50% deeper along the top of the head down the midline where it ranged from about 11 to 15 mm. This pattern reflected the same regions that had been observed to have thicker skull or CSF layers shown in Fig. 2 for the two sexes. Figure 3(c) shows the difference map of the cortical depth of the females verses the males. Regions in red color show parts of the cortex that were deeper in the females compared to the males. On average, the cortex was 0.1 mm (range [−5.6 to 4.89]; $p<1\times 10^{-15}$; $F_{30420,1}$. two-way ANOVA with position and group) deeper in the female participants. Notably, the medial and right frontal/medial cortex was between 2 and 3.5 mm deeper in the females compared to the males. The deeper depth of the cortex in the females means that fNIRS measures will be less sensitive to changes in brain activity in the females compared to the males. This could result in underestimation of brain activity in the female group from these regions and a sex-related bias in the activity in group-level analysis models.

**Fig. 4:**
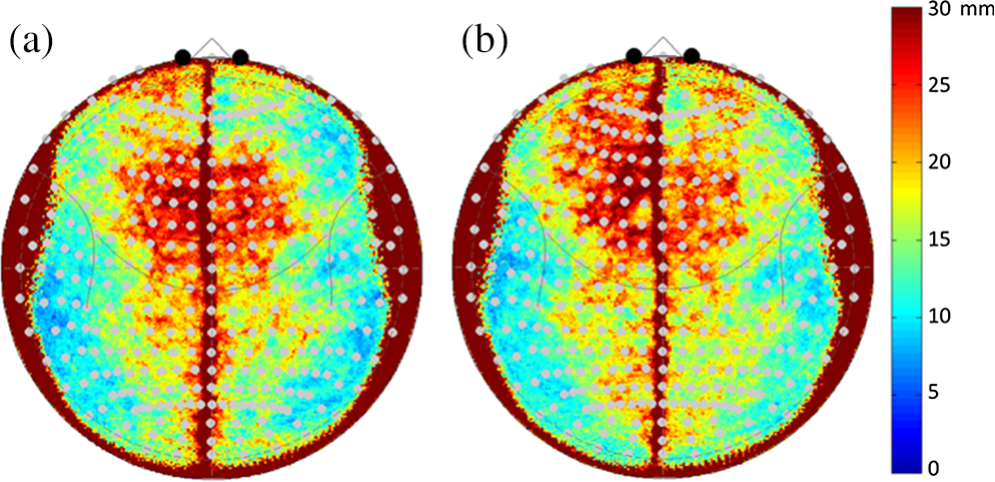
Underlying variability in brain (MNI) space measured from scalp-based fNIRS sensors. This figure shows the root-median-squared displacement in underlying registered MNI coordinates of the surface of the brain as measured from each position on the scalp. Red color indicates higher variability in the underlying structure and folding of the brain beneath each position and indicates areas where fNIRS measurements would have lower precision due to individual differences in the folding of the cortex. The results for the (a) female and (b) male subjects are shown with the same color scale.

We also examined variability in the location of the cortical folding pattern (mapped according to MNI space) relative to the 10-5 head coordinate system. Using FreeSurfer, the cortical surfaces are extracted and registered as surfaces into the “fsaverage” space where parcellation labels are assigned. In this space, the equivalent anatomical regions can be morphed from one subject’s anatomy onto another. Figure 4 shows the median displacement in the equivalent fsaverage cortical positions relative to the surface of the head in both the males and females. The most conserved anatomical regions between subjects [shown in blue-green colors in Figs. 4(a) and 4(b)] were in the lateral frontal, temporal, and posterior/occipital regions and varied from about 5 to 30 mm median displacements. The most anatomically variable regions were along the midline and superior frontal regions of the head which varied up to 20 to 30 mm. Specifically, these regions of high variability in the underlying structure of the cortical folding are the regions where the alignment of fNIRS measurements according to the 10-5 positioning on the scalp would be more sensitive to unknown variability in the underlying brain region.

**Fig. 5:**
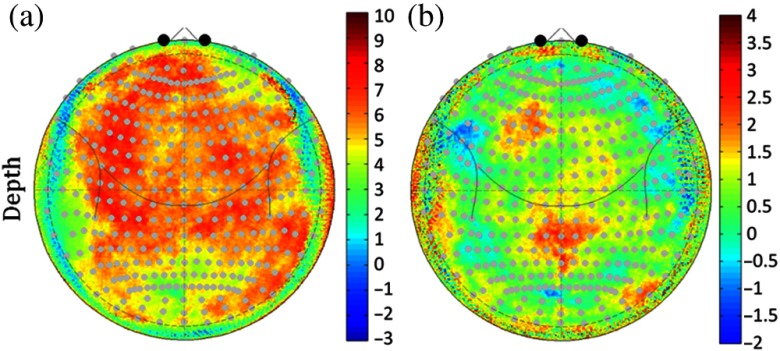
Correlation of scalp-to-brain depth with head circumference and age. This figure shows t-statistic maps (89 degrees of freedom) from the pooled data across both genders for a regression model against (a) head circumference and (b) age at time of MRI scan in months. Red indicates a positive correlation and increasing depth with circumference or age.

Finally, in Fig. 5, we looked at the effect of head size and age on the cortical depth. The registered polar (10-5 coordinate) space data for all 90 subjects across both sexes was pooled and regressed using the head circumference or subject age in a robust regression model. In Fig. 5, we show the t-statistic map for these two models. As shown in Fig. 5(a), we found that head circumference was more correlated to the depth of the brain than age. This is because over our group of subjects in this school-age range, we found that there was a considerable spread in head size, height, and weight, which was not well predicted by age alone and reflected different growth rates for the children. Specifically, head circumference was only marginally related to age ($R^{2}=0.176$). We found that the depth of the brain at the top of the head was positively related to head circumference [Fig. 5(a)], which indicates that this depth was greater for children with larger head sizes. This also indicates that the head is probably growing faster than the brain over this age range resulting in an increasing depth.

### 3.2 Variation in Optical Properties

As previously described in the methods, a Monte Carlo-based model was used to simulate fNIRS measurements for each of the 90 subjects at all 346 of the 10-5 positions on the head for wavelengths 690, 780, 808, 830, and 850 nm. These wavelengths were selected as the five most widely used wavelengths in currently commercially available fNIRS systems. At each 10-5 head position, virtual “photons” from an fNIRS emitter were simulated and mapped to all surface points symmetrically around the emitter. For each emitter position, the DPF was computed by a weighted least-squares fit of the detectors from 10 to 40 mm around a source. The differential pathlength (e.g., how far did the light travel within the tissue) is equal to the product of the DPF and the emitter–detector distance along the arc of the scalp [Eq. (1)]. Similarly, the PPF as defined in Eq. (2) was computed as the pathlength through only the cortex layer of the model. Both DPF and PPF are unitless scaling factors, which adjust the fNIRS emitter–detector distance in the MBLL.

**Fig. 6:**
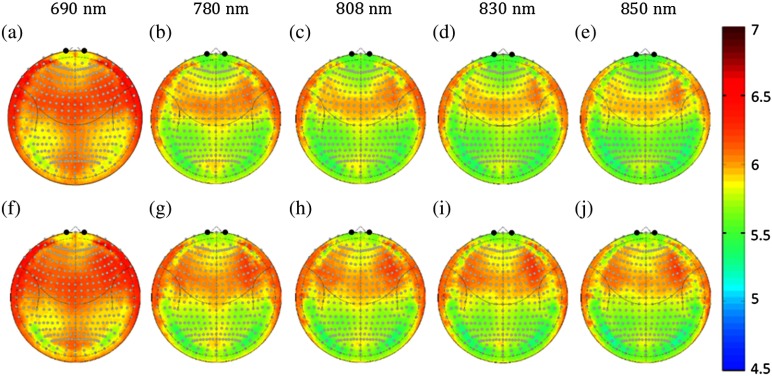
Gender and wavelength differences in the DPF. The DPF at the 5 simulated wavelengths is shown for the (a)–(e) female and (f)–(j) male subjects based on the Monte Carlo simulations. The median DPF for each position in the registered polar (10-5 coordinate) space is shown for each of the wavelengths.

Figure 6 shows the topographic maps of the estimated DPF for both sexes at all five simulated wavelengths. The DPF was highest for the 690 nm wavelength in both sexes; DPF ranged from 5.53 to 6.82 in the females (median=6.00) and from 5.38 to 6.84 in the males (median=6.04). Although lower in comparison, in all other wavelengths (780, 808, 830, and 850 nm), the median DPF between sexes was similar; 5.76, 5.71, 5.67, and 5.64 in females and 5.84, 5.79, 5.76, and 5.74 in the males, respectively). The DPF was slightly higher in the males by 1.3% to 1.6% compared to the females. Spatially, the DPF was symmetric across hemispheres and was highest along the top of the head along precentral sulcus and superior frontal cortex. The average DPF for several head regions is given in Table 3. Tables 5–15 for the DPF for both sexes and at each of the 346 10-5 coordinate positions are given in the Appendix.

**Table 3:** Sensitivity, DPF, PPF of anatomical regions (both sexes).

Region	Sensitivity (dB)	DPF (wavelength, nm)					PPF (wavelength, nm)				
		690	780	808	830	850	690	780	808	830	850
Left hemisphere											
Caudal middle frontal	−36.9	6.15	5.99	5.94	5.93	5.94	2.53	2.63	2.66	2.65	2.69
Frontal pole	−39.1	6.00	5.74	5.74	5.67	5.63	1.74	1.78	1.78	1.80	1.83
Inferior parietal	−29.7	5.98	5.75	5.73	5.70	5.66	2.47	2.43	2.45	2.50	2.54
Inferior temporal	−35.2	6.03	5.86	5.79	5.77	5.74	2.38	2.43	2.42	2.43	2.48
Lateral occipital	−27.2	6.03	5.75	5.70	5.66	5.63	2.28	2.26	2.27	2.29	2.35
Middle temporal	−28.7	6.05	5.87	5.82	5.82	5.77	2.49	2.54	2.54	2.54	2.54
Pars orbitalis	−41.1	6.10	5.93	5.92	5.92	5.88	2.39	2.44	2.47	2.48	2.49
Pars triangularis	−37.9	6.14	5.98	5.93	5.90	5.88	2.57	2.59	2.64	2.58	2.65
Postcentral	−30.7	6.13	5.94	5.91	5.88	5.87	2.63	2.70	2.71	2.72	2.76
Precentral	−33.5	6.13	6.00	5.95	5.93	5.92	2.66	2.72	2.76	2.75	2.78
Rostral middle frontal	−31.4	6.11	5.94	5.90	5.87	5.84	2.35	2.39	2.39	2.39	2.42
Superior frontal	−30.9	6.17	6.00	5.95	5.91	5.89	2.32	2.35	2.38	2.39	2.42
Superior parietal	−36.7	6.01	5.79	5.77	5.74	5.71	2.42	2.49	2.52	2.49	2.56
Superior temporal	−41.1	6.10	5.92	5.87	5.85	5.83	2.66	2.64	2.64	2.57	2.60
Supramarginal	−33.7	6.02	5.81	5.76	5.77	5.73	2.56	2.60	2.60	2.67	2.67
Right hemisphere											
Caudal middle frontal	−38.2	6.22	6.04	5.97	5.94	5.91	1.96	1.98	2.00	2.04	2.03
Frontal pole	−40.3	6.20	5.93	5.82	5.81	5.75	1.38	1.37	1.38	1.38	1.39
Inferior parietal	−31.5	5.96	5.73	5.66	5.66	5.59	1.93	1.97	1.95	2.03	2.06
Inferior temporal	−35.2	6.02	5.73	5.67	5.67	5.61	1.74	1.72	1.77	1.70	1.83
Lateral occipital	−28.7	5.95	5.68	5.62	5.56	5.54	1.73	1.75	1.74	1.77	1.79
Middle temporal	−32.4	6.02	5.78	5.71	5.69	5.66	1.70	1.71	1.72	1.73	1.75
Pars orbitalis	−39.5	6.33	6.00	5.97	5.93	5.89	1.33	1.35	1.34	1.39	1.40
Pars triangularis	−35.8	6.28	6.07	6.05	6.02	5.95	1.83	1.81	1.82	1.86	1.94
Postcentral	−29.6	6.08	5.89	5.85	5.80	5.78	1.78	1.87	1.87	1.89	1.98
Precentral	−32.0	6.12	5.92	5.87	5.83	5.79	1.82	1.90	1.89	1.92	1.96
Rostra middle frontal	−32.8	6.30	6.05	5.97	5.94	5.87	1.60	1.60	1.64	1.65	1.64
Superior frontal	−34.2	6.17	6.01	5.95	5.92	5.89	1.86	1.92	1.92	1.93	1.99
Superior parietal	−34.9	6.04	5.78	5.72	5.68	5.67	1.94	1.96	1.96	1.95	2.02
Superior temporal	−38.3	6.06	5.85	5.81	5.77	5.74	1.66	1.65	1.67	1.68	1.71
Supramarginal	−32.2	6.03	5.84	5.77	5.74	5.68	1.74	1.78	1.82	1.87	1.93

Note: This table shows the DPF and PPF for several fNIRS-accessible regions on the head. Data has been combined for both male and female subjects. The sensitivity of the region to fNIRS is provided in decibels (dB; 10×log 10) and indicates the fraction of photons normalized to the incidence power reaching this region from the scalp at a source–detector distance of 30 mm.

**Fig. 7:**
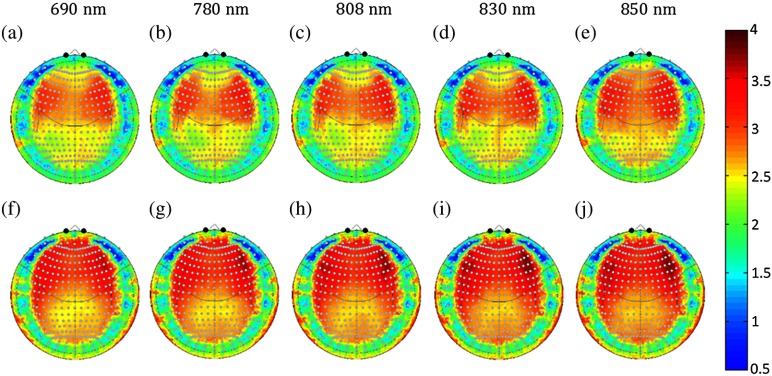
Gender and wavelength differences in the PPF. The PPF at the 5 simulated wavelengths is shown for the (a)–(e) female and (f)–(j) male subjects based on the Monte Carlo simulations. The median PPF for each position in the registered polar (10-5 coordinate) space is shown for each of the wavelengths.

Figure 7 shows the spatial distribution of the PPF for the two sexes and five simulated wavelengths. The PPF has less variation between wavelengths compared to DPF, but has a larger difference between the two sexes. The PPF was higher in the males by 15.4% to 18.8% across the whole head when compared to the females. The whole head median value of the PPF was 1.77, 1.77, 1.77, 1.80, and 1.90 for the females and 2.45, 2.44, 2.48, 2.45, and 2.45 for the males at 690, 780, 808, 830, and 850 nm, respectively. Although this difference was observed across the whole head, PPF sex differences were the greatest in the area around the medial frontal region (Fp1, FpZ, and Fp2); here females had a lower PPF value (1.15 compared to 1.45 [average of all 5 wavelengths; $p=1.5\times 10^{-4}$]). This corresponds to the same region around the frontal sinuses where the cortex was deeper among females, as shown in Fig. 3(c). The observed 2 to 3 mm increased depth in the females and corresponding decrease in the PPF directly translates to an expected 26% underestimation in fNIRS measurements of brain activity in these regions relative to males. The PPF across the head is summarized in Table 3. Due to space constraints, both males and females have been combined in Table 3. However, for both sexes, Tables 5–15 describing the 346 10-5 coordinate positions are given in the Appendix.

**Fig. 8:**
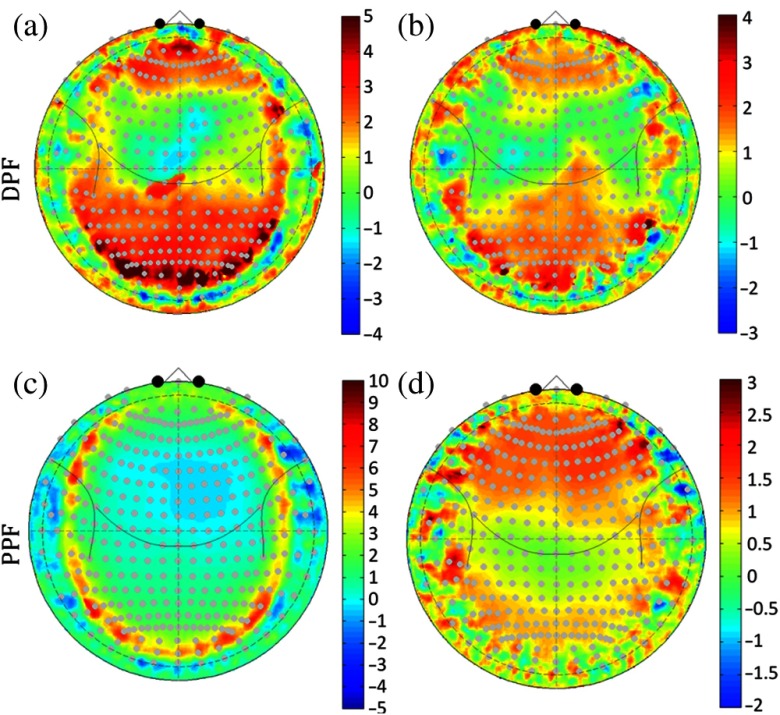
Correlation of DPF and PPF with head circumference and age. This figure shows t-statistic maps (89 degrees of freedom) from the pooled data across both genders for a regression model against (a) and (c) head circumference and (b) and (d) age at time of MRI scan in months. Red indicates a positive correlation and increasing pathlength with circumference or age.

Similar to the analysis of the scalp-to-brain depth with age and head circumference, we also examined DPF and PPF with these two regression models. Figure 8 shows the statistical result (t-statistic) from the regression model pooling data from all 90 subjects. Both head circumference and age had the biggest effect on the DPF in the frontal and occipital regions of the head with positive correction (e.g., increasing DPF with increased age or head circumference). For PPF, age was positively correlated with PPF in the frontal regions. Head circumference seemed to matter only along the equator of the head, but decreased with head circumference in most other regions. In Figure 8, variability in DPF and PPF, seen as a noticeable ring near the equator of the Clarke projection, is due to the inferior surface of the brain cutting in where it rests on the cranium floor. This is also evident in the positional variability maps shown in Fig. 4.

### 3.3 Regions-of-Interest

**Fig. 9:**
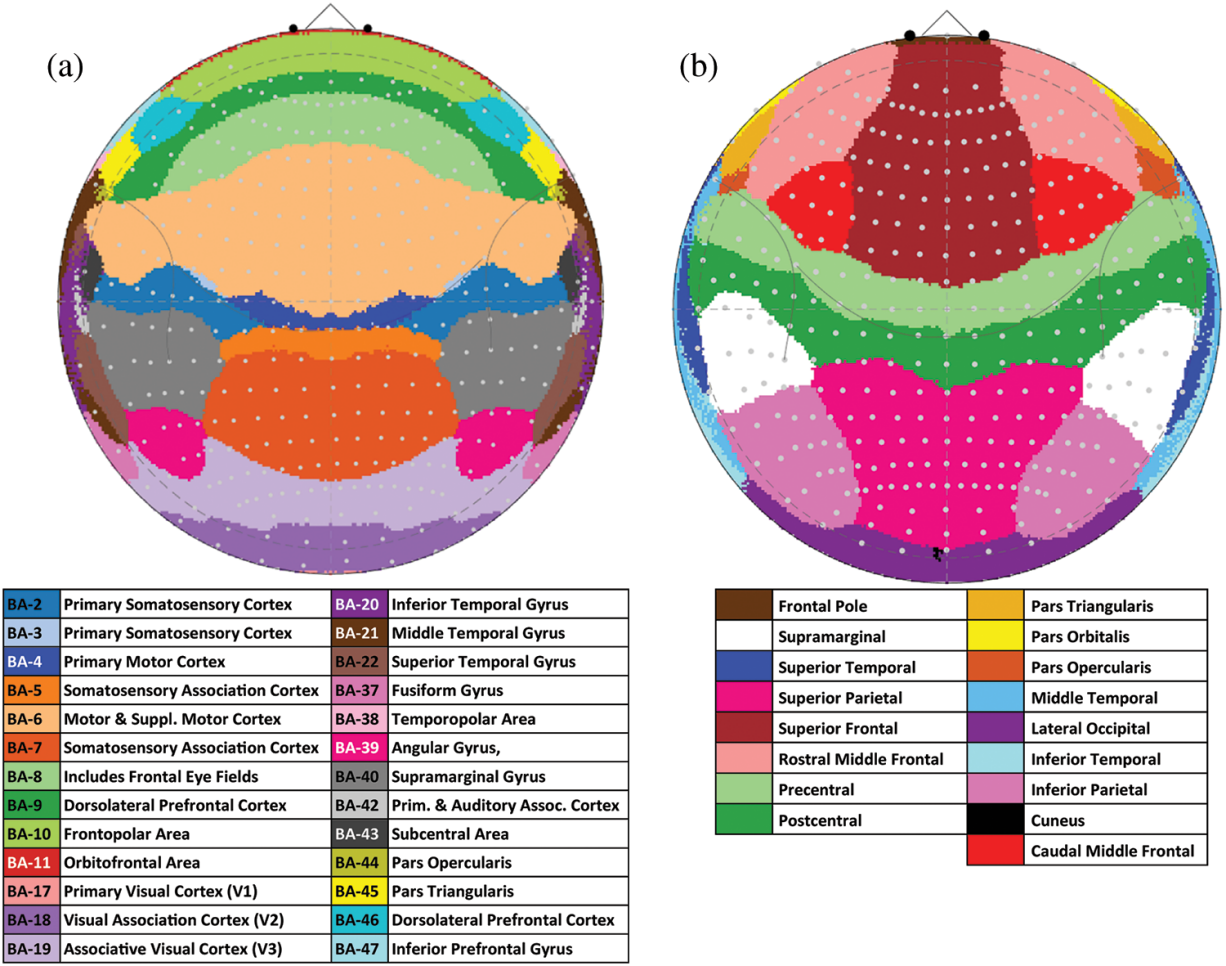
Underlying anatomical and Brodmann area maps. (a) The mode label across the 90 subjects is given for the anatomical labels of the gyri from FreeSurfer.^20^ (b) The Broadmann area labels based on the aal template^21^ are given. Both panels show the most frequent label (mode) across both genders.

The segmented MRI volumes for each of the 90 subjects were parcellated into 70 anatomical regions of the cortical surface using FreeSurfer.^27^ These parcellation labels were created for each subject and then projected into the registered polar (10-5 coordinate) space. Figure 9(a) shows the most frequent (mode) label across the head from all the subjects. Male and female subjects had only minor differences (see the Appendix). Similarly, we used the automatic anatomical labeling dataset^28^ to find Brodmann area labels on the cortical surface in MNI space [Fig. 9(b)]. As a note, the Brodmann areas from the automatic anatomical labeling dataset are defined in three-dimensional space and cover parts of the sulci as well as the gyri folds. Therefore, these regions extend to slightly deeper areas compared to the FreeSurfer gyri labels. In Table 4, we provide a list of the cortical depth and closest 10-5 head coordinate points for a subset of the most accessible of these regions. Tables 5–15 are provided in the Appendix.

**Table 4:** Nearest 10-5 location for recording Brodmann areas (both sexes).

BA	Region	Depth	Position 1		Position 2		Position 3	
		Med (min to max)	Name	Depth	Name	Depth	Name	Depth
Left hemisphere								
BA-1	Prim. somatosensory ctx	16.85 (6.31 to 26.80)	C3h	18.13	C3	18.72	C1	21.37
BA-2	Prim. somatosensory ctx	19.50 (10.24 to 26.80)	C3	18.99	C3h	20.78	C1	22.74
BA-3	Prim. somatosensory ctx	20.71 (9.70 to 27.58)	C1h	20.56	C1	22.47	C3h	23.33
BA-4	Prim. motor ctx	17.81 (9.90 to 26.61)	FCC5h	18.68	C1h	19.14	FCC3	20.32
BA-6	Motor and suppl. motor ctx	20.65 (10.38 to 25.98)	FCC1h	21.65	FCC1	22.58	FCCz	26.47
BA-7	Somatosensory assoc. ctx	23.45 (19.07 to 29.61)	CP1	21.79	CP1h	23.56	CCP1h	24.44
BA-8	Includes frontal eye fields	17.94 (7.31 to 24.82)	F1h	18.07	FFC1h	18.92	FFC1	19.12
BA-9	Dorsolateral prefrontal ctx	20.26 (12.06 to 27.22)	F1	19.51	FC3	23.68	FCC3	23.76
BA-10	Frontopolar area	19.02 (14.97 to 25.12)	F3	17.54	AFF1h	24.22	AFF1	24.30
BA-17	Prim. visual ctx (V1)	16.66 (11.62 to 26.78)	POO1	16.52	PO1	18.31	PO1h	18.93
BA-18	Visual assoc. ctx (V2)	18.50 (15.18 to 24.39)	PPO5h	17.29	PPO1h	19.21	PO1h	21.61
BA-19	Associative visual ctx (V3)	20.02 (15.59 to 24.85)	P3h	19.07	CPP3	20.58	CPP5h	21.42
BA-21	Middle temporal gyrus	21.62 (16.54 to 28.05)	CCP5	22.42	C5	24.26	FCC5	25.33
BA-22	Superior temporal gyrus	24.51 (20.95 to 28.74)	CCP5h	23.06	FCC5	25.16	FCC5h	31.26
BA-37	Fusiform gyrus	17.33 (11.79 to 24.04)	CPP5	18.30	CPP5h	18.73	CP5	19.94
BA-39	Angular gyrus	18.76 (14.61 to 26.24)	CPP3	19.12	CP3	19.78	CP3h	21.71
BA-40	Supramarginal gyrus	19.80 (13.52 to 25.98)	CCP3	19.76	CCP3h	21.97	C3h	22.80
BA-43	Subcentral area	14.70 (4.84 to 24.00)	C5h	15.29	FCC5h	16.58	FCC3	22.35
BA-45	Pars triangularis	16.35 (12.02 to 24.41)	FC5h	16.93	FFC5h	20.04	FC3	20.80
BA-46	Dorsolateral prefrontal ctx	17.49 (10.73 to 24.16)	FFC3	18.11	FFC5h	18.47	FC3	18.53
Right hemisphere								
BA-1	Prim. somatosensory ctx	16.74 (10.31 to 22.10)	C4	16.72	C4h	18.95	C2	20.88
BA-2	Prim. somatosensory ctx	18.49 (12.28 to 25.25)	C4	16.28	C4h	20.79	C2	21.86
BA-3	Prim. somatosensory ctx	17.69 (9.79 to 24.37)	C4h	17.40	C2	21.80	C2h	22.37
BA-4	Prim. motor ctx	18.65 (9.95 to 25.18)	C2h	19.95	FCC4h	23.55	C4h	24.47
BA-6	Motor and suppl. motor ctx	19.04 (10.36 to 24.12)	FCC4	17.96	FCC2h	21.64	FCC2	22.34
BA-7	Somatosensory assoc. ctx	22.87 (16.07 to 30.78)	CP2h	22.41	CP2	22.53	CCP2h	24.76
BA-8	Includes frontal eye fields	18.83 (11.15 to 24.62)	FC4h	19.64	FFC2h	20.76	FFC2	21.20
BA-9	Dorsolateral prefrontal ctx	19.45 (13.05 to 25.82)	FC4h	22.08	FC4	22.28	FCC4	22.83
BA-10	Frontopolar area	18.08 (15.34 to 23.34)	F4	17.39	AFF2	20.89	AFF2h	22.54
BA-17	Prim. visual ctx (V1)	18.20 (12.11 to 27.42)	PO4h	17.04	PO2	17.93	PO2h	18.18
BA-18	Visual assoc. ctx (V2)	18.47 (14.47 to 25.04)	PPO4	16.28	PPO2h	19.96	PO2h	20.60
BA-19	Associative visual ctx (V3)	19.05 (15.81 to 24.77)	P4h	19.07	P2	20.33	P2h	22.70
BA-21	Middle temporal gyrus	19.23 (13.02 to 25.89)	CCP6	19.31	C6	20.73	FCC6	23.57
BA-22	Superior temporal gyrus	23.32 (16.57 to 29.15)	CP6h	21.26	CCP6h	22.62	FCC6h	26.80
BA-37	Fusiform gyrus	17.75 (11.57 to 23.92)	CP6	17.18	CPP6	19.31	CPP6h	20.76
BA-39	Angular gyrus	18.70 (12.57 to 25.00)	CPP4	19.64	CP4	19.99	CP4h	20.00
BA-40	Supramarginal gyrus	20.83 (14.98 to 26.12)	CCP4	18.95	CCP4h	22.86	C4h	23.32
BA-43	Subcentral area	13.79 (5.01 to 26.59)	C6h	16.05	C4	17.64	FCC4	20.58
BA-45	Pars triangularis	14.48 (6.86 to 22.95)	FC6h	15.80	FC4	17.26	FFC6h	17.68
BA-46	Dorsolateral prefrontal ctx	16.83 (10.10 to 23.75)	FFC6h	17.63	FFC4	18.80	FC4	19.17

Note: This table provides the depth (median and range) of selected Brodmann areas based on the aal template in MNI space. For each region, the nearest three 10-5 coordinate positions and the depth of the region to this position are provided as guidance for the placement of fNIRS sensors. The individual results for the two genders are presented in the Appendix to this work.

## 4 Discussion

In this paper, we used 90 segmented MRI volumes from children ages 5 to 11 years to model the intersubject variations in head and brain anatomy and their effects on the sensitivity of fNIRS measurements. The overall objective of this paper was to quantitatively examine how variations in anatomy with age, sex, and head-size affected the optical properties of the DPF and PPF. These two factors are used in the MBLL [Eqs. (1)–(3)] to correct for the effective pathlength of light in the total tissue and brain, respectively, which in turn determines the quantitative accuracy of fNIRS. In most cases using continuous wave (CW) fNIRS recordings, the absolute quantification of the signals is not a concern. However, the potential that DPF and/or PPF could vary across spatial regions or subject demographics creates a confound that could bias group-level statistical comparisons. Namely, when DPF or PPF is ignored as a scaling factor in CW-fNIRS recordings, this assumes that these terms are constant over space/channels and subjects. This term appears in both the numerator and denominator of a t-test used in accessing brain activity from a linear model.^29^ This term would cancel only in the case of a first level model (a statistical test within a single subject and per optical channel), but not for comparing the magnitudes across channels in region-of-interest or group-level models. As an example from our results, although the exact value of PPF may be less of a concern, the finding that the PPF in the frontal cortex varied by about 20% in the frontal cortex between males and females means that there would be an expected bias toward underestimation of the magnitude of the brain signals specifically in the female group. Thus, if the magnitude of the hemodynamic response was exactly the same in the brain/cortex space of the two groups, the resulting optical measurements in the female group would be 20% smaller in these regions. While we caution against interpreting the results of statistical tests performed in fNIRS channel space as related to true differences in brain activity compared to systematic variations in anatomy, we believe that this work can provide valuable guidance toward how these biases may be addressed at the group level. Specifically, we suggest careful examination of sex difference in fNIRS datasets in future research projects.

### 4.1 Anatomical Variations in Subjects

In the first part of this paper, we examined differences in the structural anatomy of the brain with sex, head-size, and subject age based on segmentation and registration of the anatomical MRI data. In both the male and female populations, we found quite a bit of spatial variation in the depth of the brain relative to the scalp surface. While skull and CSF thicknesses were comparable across the two groups, we found that on average the cortical depth was slightly deeper in the female subjects. This difference was greatest in the frontal regions where we believe a larger sinus cavity in the female subjects is responsible for the 2 to 3 mm increase depth of the brain in these regions. As the distance between the brain and the scalp increases, the fraction of light reaching these brain regions decreases, therefore, we observed a lower PPF value in these regions for the female subjects. This is particularly relevant for fNIRS measurements that target these frontal regions due to accessibility of the forehead, absence of hair, and scientific interest in the underlying cognitive functional domains (e.g., Brodmann areas 10, 46, and 44). We also found an asymmetry in the cortical depth, with the right side being slightly deeper than the left side of the frontal lobe. This is consistent with the structural asymmetries noted previously in a similar analysis by Beuachamp et al.^15^ When we looked at head size and age, we found that head size was the better predictor of underlying anatomy rather than age in this range of 5- to 11-year-old children. Thus, a recommendation to future fNIRS studies might be to report head sizes as well as age when listing subject demographics.

In Fig. 4, we also examined variations in the cortical folding of the brain with respect to the scalp-based 10-5 mapping. We found that certain regions, including the crown of the head in both sexes and the left frontal in specifically males, tended to show more variability in the underlying cortical regions that would be measured from fNIRS sensors. For example, based on the results shown in Fig. 4, fNIRS sensors positioned on 10-5 location Fp1 (left superior frontal) in two subjects would, on average, be measuring from two different cortical regions displaced by 3 cm in registered cortical space. Conversely, regions along the lateral frontal area (e.g., around AF8/7-F8/7) are more conserved across subjects; fNIRS measurements from these regions would be expected to come from more similar cortical regions. This implies that studies focused on group-level analyses from these more variable regions should expect a lower effect size in brain activation and consequently should plan on larger sample sizes to counteract the variability in the cortical regions relative to the fNIRS sensors. Fortunately, we found that this effect was not sex specific and both males and females had similar areas of variability (Fig. 4). This means that sex is not expected to affect precision of fNIRS measurements, although we found evidence of a clear bias in the magnitude (accuracy) of the measurements due to cortical depth.

### 4.2 Effects of Variations on Optical Properties

Based on the segmented structural MRI data, we also ran Monte Carlo simulations to look at how PPF and DPF varied spatially and across age and sex. The regions that showed the greatest variations in cortical depth between sexes were also the most variable in the partial pathlength (PPF). Since optical PPF is defined as the multiplication factor to estimate the pathlength through specifically the brain [e.g., $L_{\text{brain}}=\mathrm{PPF}\times L_{\text{source-detector}}$; Eq. (2)] within the MBLL, these spatial variations have a direct impact on the quantitative report of the magnitude of estimated brain signals. In particular, we found about a 13% to 26% spatial difference going from the frontal or lateral regions of the head to superior regions (Fig. 7). This means that for the same change in hemoglobin within these brain regions, the measured optical signals in the lateral regions would predict a lower magnitude change compared to those from near the top of the head. We also noted significant sex differences in the optical PPF, particularly in the frontal cortex (Fig. 7). Specifically, the females had ∼13% to 18% smaller PPF values compared to the males. This seems to be the effect of the cortical depth of about 2 to 3 mm in the females in this region and we believe reflects a slightly larger sinus cavity at the front of the head. Unfortunately, this PPF difference implies that for the same underlying magnitude of hemoglobin changes in the brain, the optical measurements in the females are expected to be smaller. This introduces a systematic bias in group-level statistical analysis, which looks at sex as a covariate. Since the dominant source of noise in fNIRS measurements is probably physiological signals in the skin layers, which would be expected to be similar in both sexes, the lower PPF likely directly relates to a lower contrast-to-noise ratio and lower statistical effect sizes in this group. Even if a sex-adjusted DPF is applied in analysis, this difference in the expected statistical effect would have an effect on group-size and power estimates for analysis. Optical DPF did not have nearly as much spatial variation and showed only very little variation across the same regions. DPF is the multiplication factor between the source–detector distance and the total pathlength through the tissue. Although DPF can be measured directly by time-domain or frequency-domain fNIRS technologies, PPF cannot and must be modeled.

Although DPF and PPF varied over space, within a given region, they do not vary much across the five optical wavelengths examined in this work. This means that the potential for crosstalk in the separation of ${\mathrm{HbO}}_{2}$ and Hb does not seem to vary across space, although we did not explicitly model this factor. The work by Strangman et al.^24^ examined the effect of cross talk in the separation of ${\mathrm{HbO}}_{2}$ and Hb in the choice of wavelengths and how this cross-talk is affected by differences in the DPF/PPF values at different wavelengths. Our current work seems consistent with their findings in that we did not see much evidence that the level of cross-talk would be expected to vary much across space or by subject age/sex.

### 4.3 Guidance for Designing Future Studies

Tables 3 and 4 as well as Tables 5–15 in the appendix can be used to design future fNIRS studies. Table 3 provides estimates of the relative sensitivities of various brain regions to fNIRS measurements taken from the normalized count of photons able to reach these brain regions. This depended on both the spatial extent and depth of these regions. As a general guidance, based on our own experience, we believe that regions down to about −40 dB are fNIRS-accessible based on current instrument sensitivities. For example, the TechEN Inc. (Milford MA) system has an instrument noise floor of about 68 dB.^30^ Table 3 provides these values for the FreeSurfer anatomical parcellations of the cortex for a source–detector spacing of 30 mm. Tables 5–15 for Brodmann areas and by sex are also presented in the Appendix. Likewise, Table 4 provides a list of the nearest 10-5 head coordinate point to specific accessible Brodmann areas, which could be used to guide head cap designs for future studies.

### 4.4 Limitations of this Study

In this study, we used Monte Carlo modeling of the fNIRS forward solution to attempt to quantify the effects of head anatomy in this population of 5- to 11-year-old children. One limitation of this work is that our results depend on the parameters and optical properties that we used in these simulations. In particular, we used bulk optical properties for the skin, skull, CSF, and brain [gray/white] layers, but did not consider any subject or spatial variability in these values. Although both gray and white matters were segmented, the optical properties used for these two layers (Table 2) were the same. In addition, there is considerable variability in these values in the literature.^25^ Several previous papers have attempted to experimentally quantify these values using time-resolved or frequency-domain fNIRS methods. Duncan et al.^31^ used frequency-domain fNIRS on 283 subjects (ages 0 to 50 year old) in the frontal–temporal regions and examined the relationship of DPF with subject age. In that work, an equation was derived to predict DPF as a function of subject age. Additional work by Cooper^32^ examined DPF in neonates and several papers have also examined this in adults.^13^^,^^14^^,^^33^^–^^36^ The work by Scholkmann and Wolf^37^ provides a review and summary of these methods and offers a generalized model for the DPF in the frontal cortex. In comparison to this previous literature, our results are generally consistent with DPF being between about 4 to 6.5. Of note however, the work by Bonnery et al.^14^ did suggest some discrepancies between time-resolved experimental fNIRS measurements of DPF and Monte Carlo simulations. Therefore, we caution anyone trying to use the values provided in this work to provide quantitative accuracy. This work is intended as guidance for the design and interpretation of fNIRS studies in school-aged children. For example, we used the same optical absorption and scattering for all simulations, but it is possible that these values might change over age or the values used may offer some bias in the results. This study is not intended to substitute for direct measurements of DPF and optical properties that could be obtained by time-domain or frequency-domain fNIRS techniques. Further experimental work is still needed to verify these results.

A second limitation of this work is that our analysis of DPF and PPF in this study was based on the collection of data from simulated measurements of emitter–detectors spacing from 10 to 40 mm. The MBLL [Eq. (2)] assumes that the pathlength correction is linear with this spacing, however, this is probably not entirely true in regions with thick CSF layers such as the frontal pole. In particular, for short spacings or areas where the brain is deeper, the PPF term would be particularly sensitive to nonlinearities.

## Appendix:

Provided below are the tables describing the results of the Monte-Carlo simulations. Tables 5–14 provide information by either Brodmann area or anatomical region.

Where Table 3 in the text describes the sensitivity, DPF, and PPF of each wavelength by select anatomical regions, Table 5 describes these values for select Brodmann areas. Similarly, where Table 4 in the text describes the nearest 10-5 locations for select Brodmann areas, Table 6 describes these locations for select anatomical regions.

Tables 3–6 contain our findings for select areas/regions for both sexes and Tables 7–10 are separated by sex.

Tables 7–10 are the complete results of our sensitivity, DPF, and PPF findings specifically divided by sex and explained by either Brodmann areas or anatomical regions. Tables 11–14 are the complete results of the nearest 10-5 locations divided by sex and explained by either Brodmann areas or anatomical regions.

Finally, Table 15 contains the results of our generalized equation for DPF for each 10-5 location.

**Table 5:** Sensitivity, DPF, and PPF of select Brodmann areas (both sexes).

BA	Region	Sens.	DPF (nm)					PPF (nm)				
			690	780	808	830	850	690	780	808	830	850
BA-1	L prim. somatosensory ctx	−42.9	6.11	5.95	5.90	5.89	5.88	2.67	2.75	2.79	2.77	2.84
BA-1	R prim. somatosensory ctx	−36.4	6.13	5.91	5.88	5.78	5.78	1.78	1.85	1.86	1.86	1.91
BA-2	L prim. somatosensory ctx	−37.1	6.13	5.94	5.90	5.89	5.86	2.62	2.66	2.71	2.66	2.73
BA-2	R prim. somatosensory ctx	−35.5	6.11	5.88	5.79	5.76	5.74	1.87	1.90	1.96	1.96	2.08
BA-3	L prim. somatosensory ctx	−40.0	6.12	5.95	5.92	5.87	5.85	2.62	2.69	2.70	2.72	2.75
BA-3	R prim. somatosensory ctx	−35.0	6.13	5.87	5.83	5.79	5.77	1.96	1.99	2.04	2.01	2.07
BA-4	L prim. motor ctx	−40.6	6.14	6.00	5.95	5.88	5.92	2.60	2.66	2.72	2.71	2.74
BA-4	R prim. motor ctx	−41.2	6.08	5.83	5.78	5.76	5.73	1.81	1.85	1.87	1.89	1.94
BA-6	L motor and suppl. motor ctx	−34.2	6.18	5.99	5.97	5.93	5.91	2.62	2.55	2.59	2.59	2.67
BA-6	R motor and suppl. motor ctx	−27.9	6.16	5.93	5.92	5.85	5.85	1.90	1.94	1.94	1.96	2.02
BA-7	L somatosensory assoc. ctx	−36.5	6.03	5.80	5.75	5.72	5.68	2.33	2.29	2.35	2.32	2.37
BA-7	R somatosensory assoc. ctx	−37.2	6.03	5.78	5.73	5.71	5.69	2.05	2.07	2.07	2.13	2.14
BA-8	L includes frontal eye fields	−33.4	6.20	6.04	5.95	5.94	5.93	2.24	2.32	2.33	2.32	2.39
BA-8	R includes frontal eye fields	−35.8	6.29	6.07	6.05	6.01	5.96	1.92	2.01	2.02	2.01	2.05
BA-9	L dorsolateral prefrontal ctx	−34.5	6.15	5.95	5.93	5.95	5.91	2.43	2.48	2.52	2.49	2.51
BA-9	R dorsolateral prefrontal ctx	−39.6	6.31	6.04	5.98	5.93	5.91	1.72	1.74	1.77	1.78	1.78
BA-10	L frontopolar area	−30.7	6.04	5.86	5.82	5.76	5.74	1.98	1.91	1.92	1.95	2.04
BA-10	R frontopolar area	−30.8	6.22	5.99	5.91	5.85	5.81	1.55	1.56	1.57	1.61	1.59
BA-17	L prim. visual ctx (V1)	−39.6	5.99	5.73	5.70	5.64	5.61	1.99	1.93	1.97	1.97	2.05
BA-17	R prim. visual ctx (V1)	−41.7	5.97	5.71	5.63	5.58	5.56	1.62	1.66	1.64	1.66	1.70
BA-18	L visual assoc. ctx (V2)	−28.7	6.03	5.75	5.70	5.64	5.59	2.15	2.14	2.18	2.19	2.22
BA-18	R visual assoc. ctx (V2)	−27.9	5.94	5.66	5.61	5.54	5.51	1.56	1.57	1.59	1.64	1.73
BA-19	L associative visual ctx (V3)	−30.3	5.98	5.77	5.72	5.68	5.65	2.43	2.39	2.41	2.42	2.53
BA-19	R associative visual ctx (V3)	−31.8	5.93	5.68	5.62	5.59	5.56	2.00	1.96	1.98	2.00	2.10
BA-21	L middle temporal gyrus	−37.2	6.08	5.90	5.84	5.81	5.80	2.58	2.64	2.63	2.59	2.58
BA-21	R middle temporal gyrus	−34.0	6.01	5.80	5.77	5.73	5.69	1.68	1.61	1.64	1.67	1.76
BA-22	L superior temporal gyrus	−38.9	6.09	5.94	5.91	5.89	5.86	2.69	2.72	2.76	2.70	2.74
BA-22	R superior temporal gyrus	−36.0	6.07	5.86	5.82	5.79	5.74	1.71	1.73	1.76	1.78	1.78
BA-37	L fusiform gyrus	−37.9	5.97	5.76	5.70	5.67	5.63	2.38	2.35	2.40	2.45	2.52
BA-37	R fusiform gyrus	−34.3	5.96	5.70	5.64	5.61	5.58	1.74	1.73	1.77	1.83	1.83
BA-39	L angular gyrus	−35.7	5.97	5.77	5.73	5.70	5.68	2.25	2.29	2.31	2.36	2.44
BA-39	R angular gyrus	−36.5	5.96	5.72	5.65	5.65	5.61	1.86	1.92	1.92	1.98	2.02
BA-40	L supramarginal gyrus	−32.9	6.05	5.83	5.81	5.77	5.76	2.51	2.54	2.57	2.58	2.63
BA-40	R supramarginal gyrus	−31.0	6.03	5.83	5.79	5.75	5.72	1.72	1.75	1.77	1.82	1.84
BA-43	L subcentral area	−37.7	6.15	6.02	5.99	5.97	5.99	2.71	2.75	2.76	2.76	2.83
BA-43	R subcentral area	−40.0	6.08	5.86	5.84	5.79	5.76	1.75	1.78	1.82	1.84	1.83
BA-45	L pars triangularis	−39.6	6.15	5.98	5.95	5.90	5.91	2.61	2.63	2.65	2.62	2.68
BA-45	R pars triangularis	−39.4	6.30	6.07	6.01	5.96	5.93	1.77	1.83	1.81	1.86	1.86
BA-46	L dorsolateral prefrontal ctx	−36.4	6.17	5.97	5.94	5.89	5.89	2.48	2.52	2.52	2.48	2.50
BA-46	R dorsolateral prefrontal ctx	−36.6	6.29	6.02	5.96	5.94	5.88	1.63	1.64	1.67	1.71	1.77

Note: This table shows the differential (DPF) and PPF for several fNIRS-accessible Brodmann areas in the head. Data have been combined for both male and female subjects. The sensitivity of the region to fNIRS is provided in decibels (dB; 27 10×log 10) and indicates the fraction of photons normalized to the incidence power reaching this region from the scalp at a source–detector distance of 30 mm.

**Table 6:** Nearest 10-5 location for recording select anatomical regions (both sexes).

Region	Depth	Position 1		Position 2		Position 3	
	Med (min to max)	Name	Depth	Name	Depth	Name	Depth
L caudal middle frontal	20.33 (11.99 to 26.63)	FC3h	19.50	FCC3h	21.01	FC3	25.10
R caudal middle frontal	20.46 (11.36 to 25.70)	FC2	20.20	FCC2	20.43	FC4h	22.09
L frontal pole	14.70 (10.80 to 21.25)	AFp1	14.68	AFp3h	14.73	Fp1h	16.59
R frontal pole	15.34 (12.08 to 22.68)	AFp4h	15.30	AFp2h	15.80	Fp2h	16.95
L inferior parietal	18.20 (13.25 to 22.86)	CPP3h	17.98	CPP3	20.18	CPP5h	23.19
R inferior parietal	17.93 (13.18 to 23.42)	CP4h	19.01	CPP4	19.76	CP4	20.03
L inferior temporal	20.05 (15.60 to 28.07)	CP5	18.81	CPP5	19.88	TP7h	21.93
R inferior temporal	19.70 (13.89 to 27.26)	CPP6	18.68	CP6	18.78	T8h	26.15
L lateral occipital	14.15 (11.12 to 21.28)	PPO5h	15.33	PPO3	16.59	P5h	18.59
R lateral occipital	14.09 (11.54 to 21.02)	PPO4h	16.03	PPO4	16.07	P6h	17.84
L middle temporal	16.19 (12.21 to 21.67)	CP5	15.68	C5	20.58	CCP5	20.67
R middle temporal	16.27 (9.52 to 21.80)	CCP6	18.15	C6	19.15	FTT8h	22.78
L pars orbitalis	18.15 (13.52 to 29.05)	F5	18.28	FFC5	19.73	FC5	20.81
R pars orbitalis	17.86 (13.32 to 28.56)	F6h	17.99	F6	18.67	FFC6	19.17
L pars triangularis	17.64 (13.52 to 24.87)	FFC5	18.56	FC5h	18.59	FFC5h	21.27
R pars triangularis	17.11 (10.76 to 26.09)	FC6h	17.82	FFC4	19.32	FFC6h	20.21
L postcentral	18.22 (10.85 to 26.28)	C3	18.75	C3h	19.41	C5h	20.42
R postcentral	18.11 (11.33 to 24.70)	C4	17.97	C4h	19.76	C2	21.15
L precentral	20.46 (11.63 to 26.01)	FCC5h	20.29	FCC3	20.41	C1	22.97
R precentral	20.27 (12.58 to 26.21)	FCC4	19.37	FCC4h	20.50	C2	23.69
L rostral middle frontal	16.29 (11.35 to 21.98)	FFC3	18.19	FFC3h	18.84	F3h	19.07
R rostral middle frontal	17.21 (11.97 to 22.37)	FFC4	19.11	FC4	19.37	FFC4h	19.56
L superior frontal	20.57 (10.86 to 27.77)	FFC1h	19.70	FC1h	21.96	FCC1h	23.38
R superior frontal	21.69 (12.48 to 27.22)	FFC2h	21.41	FC2h	22.46	FCC2h	22.80
L superior parietal	20.97 (16.07 to 28.94)	CP1	22.01	CCP1	22.59	CPP1	22.68
R superior parietal	20.93 (16.19 to 27.98)	CP2h	20.94	CP2	23.51	CCP2	24.15
L superior temporal	22.43 (18.26 to 27.88)	FCC5	23.85	C5	23.96	CCP5h	24.42
R superior temporal	22.26 (13.63 to 28.74)	CCP6h	22.24	FCC6	23.81	C6	25.09

Note: This table provides the depth (median and range) of selected FreeSurfer anatomical regions. Data have been combined for both male and female subjects. For each region, the nearest three 10-5 coordinate positions and the depth of the region to this position is provided as guidance for the placement of fNIRS sensors.

**Table 7:** Sensitivity, DPF, PPF of Brodmann areas (females).

BA	Region	Sens.	DPF (nm)					PPF (nm)				
			690	780	808	830	850	690	780	808	830	850
BA-1	L prim. somatosensory ctx	−44.1	6.06	5.91	5.88	5.87	5.82	2.49	2.52	2.54	2.55	2.66
BA-1	R prim. somatosensory ctx	−36.2	6.08	5.90	5.79	5.78	5.78	1.63	1.73	1.71	1.76	1.81
BA-2	L prim. somatosensory ctx	−36.4	6.10	5.93	5.88	5.87	5.85	2.42	2.49	2.48	2.52	2.60
BA-2	R prim. somatosensory ctx	−34.7	6.05	5.84	5.80	5.77	5.81	1.82	1.88	1.95	1.95	2.03
BA-3	L prim. somatosensory ctx	−43.1	6.11	5.91	5.89	5.82	5.82	2.40	2.45	2.41	2.47	2.59
BA-3	R prim. somatosensory ctx	−36.3	6.13	5.85	5.84	5.79	5.80	1.90	1.92	1.99	1.97	2.03
BA-4	L prim. motor ctx	−41.4	6.10	5.90	5.87	5.84	5.81	2.40	2.49	2.44	2.52	2.61
BA-4	R prim. motor ctx	−41.3	5.98	5.79	5.76	5.75	5.75	1.72	1.77	1.81	1.85	1.86
BA-5	L somatosensory assoc. ctx	−44.7	6.08	5.84	5.78	5.76	5.73	2.00	2.03	2.12	2.13	2.20
BA-5	R somatosensory assoc. ctx	−44.9	6.06	5.80	5.76	5.73	5.74	1.86	1.94	1.90	2.02	2.11
BA-6	L motor and suppl. motor ctx	−35.3	6.18	5.97	5.96	5.93	5.88	2.21	2.43	2.44	2.46	2.52
BA-6	R motor and suppl. motor ctx	−27.2	6.21	5.97	5.91	5.87	5.88	1.75	1.84	1.86	1.88	1.94
BA-7	L somatosensory assoc. ctx	−37.5	5.96	5.73	5.70	5.64	5.60	2.13	2.13	2.15	2.19	2.23
BA-7	R somatosensory assoc. ctx	−37.3	6.02	5.73	5.67	5.63	5.60	1.86	2.00	1.96	2.07	2.12
BA-8	L includes frontal eye fields	−34.0	6.18	5.98	5.94	5.90	5.91	1.93	2.03	2.04	2.09	2.16
BA-8	R includes frontal eye fields	−35.7	6.35	6.09	6.06	6.02	5.96	1.64	1.68	1.70	1.74	1.73
BA-9	L dorsolateral prefrontal ctx	−34.1	6.08	5.88	5.88	5.82	5.86	2.40	2.36	2.43	2.38	2.47
BA-9	R dorsolateral prefrontal ctx	−39.5	6.35	6.03	5.98	5.93	5.91	1.46	1.57	1.57	1.60	1.65
BA–10	L frontopolar area	−31.4	6.00	5.85	5.74	5.73	5.70	1.65	1.70	1.71	1.75	1.78
BA–10	R frontopolar area	−31.1	6.21	6.00	5.92	5.85	5.82	1.34	1.41	1.41	1.40	1.39
BA–11	L orbitofrontal area	−58.3	6.02	5.79	5.75	5.70	5.65	1.64	1.63	1.66	1.69	1.70
BA–11	R orbitofrontal area	−46.0	6.26	5.93	5.87	5.87	5.81	1.19	1.18	1.18	1.21	1.24
BA–17	L prim. visual ctx (V1)	−38.0	6.01	5.71	5.63	5.59	5.51	1.50	1.57	1.58	1.62	1.70
BA–17	R prim. visual ctx (V1)	−41.4	6.00	5.67	5.60	5.52	5.49	1.22	1.25	1.27	1.33	1.37
BA–18	L visual assoc. ctx (V2)	−28.0	6.03	5.70	5.65	5.60	5.55	1.61	1.66	1.67	1.73	1.90
BA–18	R visual assoc. ctx (V2)	−28.6	5.96	5.68	5.62	5.52	5.48	1.18	1.23	1.25	1.28	1.36
BA–19	L associative visual ctx (V3)	−32.6	6.02	5.76	5.68	5.63	5.60	1.89	1.94	2.00	2.04	2.15
BA–19	R associative visual ctx (V3)	−31.4	5.94	5.65	5.59	5.58	5.54	1.68	1.71	1.72	1.75	1.79
BA–20	L inferior temporal gyrus	−49.6	6.02	5.90	5.86	5.82	5.84	2.39	2.51	2.53	2.53	2.56
BA–20	R inferior temporal gyrus	−43.0	6.00	5.75	5.72	5.66	5.64	1.80	1.76	1.77	1.71	1.81
BA–21	L middle temporal gyrus	−36.2	6.06	5.89	5.84	5.78	5.78	2.54	2.61	2.58	2.55	2.56
BA–21	R middle temporal gyrus	−31.0	6.02	5.82	5.82	5.77	5.72	1.82	1.82	1.80	1.67	1.86
BA-22	L superior temporal gyrus	−39.1	6.09	5.90	5.85	5.85	5.82	2.32	2.39	2.40	2.39	2.47
BA-22	R superior temporal gyrus	−36.7	6.12	5.88	5.83	5.78	5.74	1.73	1.74	1.77	1.77	1.85
BA-23	L ventral post. cingulate ctx	−85.5	6.06	5.90	5.83	5.83	5.78	2.10	2.20	2.27	2.36	2.36
BA-23	R ventral post. cingulate ctx	−80.0	6.10	5.90	5.84	5.80	5.82	1.66	1.79	1.88	2.03	2.21
BA-24	L ventral ant. cingulate ctx	−74.1	6.21	6.01	5.93	5.92	5.87	2.44	2.52	2.61	2.58	2.65
BA-24	R ventral ant. cingulate ctx	−68.6	6.15	5.95	5.86	5.84	5.80	1.82	1.88	1.88	1.91	2.00
BA-25	L subgenual ctx	−78.8	6.07	5.89	5.80	5.82	5.79	1.62	1.73	1.71	1.77	1.92
BA–25	R subgenual ctx	−67.3	6.20	6.00	5.91	5.90	5.83	1.45	1.41	1.41	1.49	1.56
BA-28	L post. entorhinal ctx	−88.0	6.17	5.98	5.89	5.86	5.86	2.35	2.43	2.43	2.40	2.53
BA-28	R post. entorhinal ctx	−84.3	6.08	5.83	5.82	5.73	5.71	1.54	1.55	1.56	1.52	1.52
BA-29	L retrosplenial cingular ctx	−78.9	5.96	5.74	5.66	5.64	5.59	1.87	1.92	1.96	1.96	2.12
BA-29	R retrosplenial cingular ctx	−81.3	5.96	5.72	5.68	5.62	5.60	1.83	1.93	1.90	1.98	2.03
BA-30	L part of cingular ctx	−69.7	6.01	5.73	5.70	5.63	5.59	1.80	1.86	1.86	1.90	1.97
BA-30	R part of cingular ctx	−71.4	5.91	5.61	5.59	5.54	5.52	1.58	1.66	1.62	1.66	1.67
BA-33	L part of ant. cingulate gyrus	−70.9	6.18	6.00	5.97	5.92	5.90	2.12	2.20	2.20	2.23	2.33
BA-33	R part of ant. cingulate gyrus	−64.8	6.28	6.07	5.99	5.95	5.97	1.64	1.71	1.73	1.75	1.79
BA-34	L ant. entorhinal ctx	−89.6	6.17	5.98	5.90	5.82	5.83	2.36	2.42	2.40	2.41	2.42
BA-34	R ant. entorhinal ctx	−87.0	6.09	5.79	5.74	5.68	5.69	1.52	1.51	1.56	1.49	1.42
BA-35	L perirhinal ctx	−90.0	6.04	5.83	5.79	5.78	5.74	2.22	2.31	2.32	2.27	2.32
BA-35	R perirhinal ctx	−83.8	5.99	5.76	5.72	5.69	5.68	1.55	1.52	1.53	1.52	1.60
BA-36	L parahippocampal ctx	−73.5	6.03	5.86	5.82	5.78	5.75	2.32	2.36	2.38	2.37	2.40
BA-36	R parahippocampal ctx	−62.5	6.04	5.80	5.73	5.72	5.71	1.65	1.66	1.68	1.61	1.69
BA-37	L fusiform gyrus	−37.4	5.99	5.79	5.76	5.75	5.69	2.22	2.27	2.23	2.27	2.39
BA-37	R fusiform gyrus	−33.4	5.91	5.66	5.61	5.60	5.50	1.55	1.58	1.59	1.62	1.64
BA-38	L temporopolar area	−58.3	6.19	6.02	5.93	5.89	5.90	2.29	2.39	2.40	2.37	2.44
BA-38	R temporopolar area	−53.0	6.13	5.86	5.86	5.77	5.74	1.52	1.50	1.50	1.47	1.54
BA-39	L angular gyrus	−36.4	5.97	5.73	5.66	5.67	5.60	2.09	2.06	2.07	2.14	2.20
BA-39	R angular gyrus	−35.3	5.95	5.72	5.65	5.66	5.61	1.69	1.81	1.81	1.86	1.96
BA-40	L supramarginal gyrus	−32.5	6.03	5.82	5.78	5.75	5.74	2.25	2.31	2.35	2.30	2.40
BA-40	R supramarginal gyrus	−30.6	6.04	5.84	5.79	5.76	5.73	1.62	1.71	1.72	1.75	1.83
BA-42	L prim. and auditory assoc. ctx	−47.9	6.04	5.87	5.83	5.82	5.82	2.60	2.71	2.71	2.70	2.67
BA-42	R prim. and auditory assoc. ctx	−41.0	6.08	5.82	5.77	5.69	5.67	1.52	1.61	1.55	1.61	1.64
BA-43	L subcentral area	−37.7	6.15	5.90	5.94	5.87	5.89	2.49	2.54	2.61	2.55	2.58
BA-43	R subcentral area	−38.6	6.11	5.86	5.85	5.77	5.76	1.71	1.78	1.82	1.83	1.83
BA-44	L pars opercularis	−42.4	6.19	6.05	6.01	6.00	5.96	2.38	2.51	2.55	2.41	2.58
BA-44	R pars opercularis	−43.5	6.23	6.04	5.97	5.96	5.85	1.61	1.53	1.57	1.74	1.71
BA-45	L pars triangularis	−39.2	6.14	5.97	5.97	5.90	5.89	2.41	2.54	2.59	2.49	2.57
BA-45	R pars triangularis	−39.4	6.33	6.13	6.04	5.99	5.91	1.89	1.91	1.86	1.93	1.96
BA-46	L dorsolateral prefrontal ctx	−36.2	6.12	5.87	5.83	5.79	5.79	2.29	2.34	2.37	2.40	2.42
BA-46	R dorsolateral prefrontal ctx	−35.8	6.35	6.11	5.99	5.96	5.87	1.41	1.45	1.47	1.52	1.57
BA-47	L inferior prefrontal gyrus	−44.9	6.11	5.89	5.84	5.80	5.81	2.30	2.35	2.38	2.38	2.44
BA-47	R inferior prefrontal gyrus	−49.1	6.26	6.01	5.94	5.87	5.91	1.51	1.51	1.52	1.53	1.59

Note: This table shows the DPF and PPF for several fNIRS-accessible Brodmann areas in the head in female subjects. The sensitivity of the region to fNIRS is provided in decibels (dB; 10×log 10) and indicates the fraction of photons normalized to the incidence power reaching this region from the scalp at a source–detector distance of 30 mm.

**Table 8:** Sensitivity, DPF, PPF of Brodmann areas (males).

BA	Region	Sens.	DPF (nm)					PPF (nm)				
			690	780	808	830	850	690	780	808	830	850
BA-1	L prim. somatosensory ctx	−41.6	6.18	6.03	5.95	5.93	5.91	2.73	2.81	2.82	2.79	2.85
BA-1	R prim. somatosensory ctx	−36.6	6.15	5.93	5.89	5.79	5.80	2.15	2.14	2.08	2.19	2.23
BA-2	L prim. somatosensory ctx	−38.0	6.16	5.97	5.91	5.92	5.86	2.70	2.76	2.78	2.80	2.80
BA-2	R prim. somatosensory ctx	−36.5	6.14	5.88	5.79	5.75	5.74	2.13	2.15	2.10	2.18	2.20
BA-3	L prim. somatosensory ctx	−39.1	6.13	6.01	5.99	5.98	5.93	2.74	2.80	2.84	2.80	2.82
BA-3	R prim. somatosensory ctx	−34.5	6.13	5.89	5.83	5.78	5.76	2.14	2.19	2.21	2.19	2.23
BA4	L prim. motor ctx	−39.8	6.15	6.05	6.04	6.03	6.00	2.71	2.78	2.83	2.75	2.85
BA-4	R prim. motor ctx	−40.9	6.09	5.85	5.81	5.76	5.73	2.21	2.24	2.23	2.18	2.24
BA-5	L somatosensory assoc. ctx	−39.8	6.07	5.88	5.89	5.85	5.85	2.63	2.66	2.70	2.68	2.71
BA-5	R somatosensory assoc. ctx	−44.8	6.10	5.87	5.84	5.79	5.78	2.08	2.16	2.15	2.17	2.19
BA-6	L motor and suppl. motor ctx	−32.1	6.18	6.03	6.01	5.99	5.96	2.71	2.79	2.79	2.82	2.80
BA-6	R motor and suppl.motor ctx	−28.9	6.15	5.92	5.92	5.84	5.81	2.11	2.14	2.17	2.18	2.18
BA-7	L somatosensory assoc. ctx	−35.4	6.06	5.91	5.85	5.82	5.80	2.66	2.71	2.72	2.73	2.77
BA-7	R somatosensory assoc. ctx	−36.9	6.08	5.86	5.80	5.75	5.74	2.16	2.21	2.23	2.18	2.20
BA-8	L includes frontal eye fields	−34.4	6.22	6.09	6.00	5.99	5.95	2.56	2.65	2.61	2.53	2.56
BA-8	R includes frontal eye fields	−35.9	6.26	6.07	6.05	5.97	5.97	2.10	2.08	2.06	2.09	2.14
BA-9	L dorsolateral prefrontal ctx	−34.8	6.21	6.06	6.01	6.00	5.98	2.64	2.58	2.58	2.53	2.58
BA-9	R dorsolateral prefrontal ctx	−36.3	6.29	6.04	5.97	5.94	5.90	1.97	1.96	2.01	1.95	2.02
BA-10	L frontopolar area	−30.0	6.06	5.88	5.87	5.84	5.84	2.18	2.19	2.23	2.19	2.23
BA-10	R frontopolar area	−30.5	6.23	5.95	5.89	5.84	5.79	1.61	1.61	1.62	1.63	1.63
BA-11	L orbitofrontal area	−49.2	6.10	5.88	5.86	5.80	5.75	1.91	1.94	1.90	1.84	1.85
BA-11	R orbitofrontal area	−48.5	6.24	5.98	5.93	5.91	5.84	1.32	1.31	1.32	1.38	1.39
BA-17	L prim. visual ctx (V1)	−38.9	5.98	5.75	5.78	5.77	5.72	2.42	2.41	2.40	2.38	2.42
BA-17	R prim. visual ctx (V1)	−40.2	5.96	5.72	5.68	5.63	5.60	2.15	2.11	2.08	2.01	2.03
BA-18	L visual assoc. ctx (V2)	−28.7	6.02	5.81	5.79	5.73	5.71	2.57	2.54	2.57	2.50	2.53
BA-18	R visual assoc. ctx (V2)	−28.0	5.93	5.63	5.61	5.54	5.51	2.09	2.14	2.16	2.15	2.18
BA-19	L associative visual ctx (V3)	−30.1	5.97	5.79	5.75	5.72	5.70	2.67	2.66	2.70	2.74	2.74
BA-19	R associative visual ctx (V3)	−31.2	5.92	5.68	5.64	5.63	5.59	2.25	2.30	2.28	2.33	2.36
BA-20	L inferior temporal gyrus	−45.0	6.09	5.92	5.90	5.84	5.88	2.76	2.78	2.81	2.79	2.80
BA-20	R inferior temporal gyrus	−50.4	5.99	5.78	5.71	5.66	5.65	1.82	1.86	1.86	1.88	1.88
BA-21	L middle temporal gyrus	−36.7	6.08	5.90	5.88	5.84	5.81	2.62	2.68	2.75	2.63	2.63
BA-21	R middle temporal gyrus	−36.5	6.00	5.76	5.73	5.68	5.63	1.58	1.61	1.60	1.67	1.64
BA-22	L superior temporal gyrus	−38.6	6.09	5.99	5.94	5.90	5.90	2.73	2.78	2.80	2.84	2.87
BA-22	R superior temporal gyrus	−36.1	6.06	5.86	5.79	5.80	5.75	1.70	1.73	1.76	1.78	1.75
BA-23	L ventral post. cingulate ctx	−86.4	6.12	5.96	5.97	5.91	5.89	2.46	2.38	2.52	2.38	2.38
BA-23	R ventral post. cingulate ctx	−81.6	5.97	5.85	5.78	5.79	5.75	1.97	1.97	1.97	2.01	2.01
BA-24	L ventral ant. cingulate ctx	−74.9	6.18	5.98	5.89	5.94	5.87	2.52	2.51	2.57	2.51	2.56
BA-24	R ventral ant. cingulate ctx	−75.4	6.05	5.85	5.81	5.79	5.82	2.05	2.03	2.09	2.14	2.14
BA-25	L subgenual ctx	−77.0	6.20	5.98	5.94	5.91	5.87	2.03	2.09	2.07	2.07	2.10
BA-25	R subgenual ctx	−64.1	6.25	6.01	5.95	5.93	5.90	2.06	2.08	2.11	2.15	2.15
BA-28	L post. entorhinal CTx	−85.4	6.22	6.07	6.02	6.00	5.98	2.81	2.93	3.04	2.94	2.94
BA-28	R post. entorhinal ctx	−86.1	6.02	5.76	5.76	5.70	5.71	1.90	1.89	1.87	2.01	1.98
BA-29	L retrosplenial cingular ctx	−79.4	6.02	5.82	5.79	5.75	5.71	2.67	2.66	2.66	2.63	2.63
BA-29	R retrosplenial cingular ctx	−81.2	6.00	5.83	5.78	5.74	5.77	2.46	2.42	2.42	2.40	2.42
BA-30	L part of cingular ctx	−70.0	6.00	5.84	5.77	5.73	5.70	2.79	2.78	2.81	2.86	2.83
BA-30	R part of cingular ctx	−70.5	5.93	5.64	5.60	5.55	5.52	2.16	2.11	2.14	2.24	2.25
BA-33	L part of ant. cingulate gyrus	−72.5	6.25	6.06	6.06	6.00	5.97	2.54	2.55	2.64	2.59	2.57
BA-33	R part of ant. cingulate gyrus	−62.3	6.22	6.06	6.05	6.02	5.99	2.08	2.12	2.12	2.09	2.10
BA-34	L ant. entorhinal ctx	−90.4	6.12	6.06	5.98	5.95	5.91	2.66	2.73	2.78	2.80	2.76
BA-34	R ant. entorhinal ctx	−87.6	6.10	5.81	5.77	5.74	5.72	2.04	2.03	2.00	2.06	2.08
BA-35	L perirhinal ctx	−90.6	6.02	5.84	5.80	5.76	5.76	2.58	2.64	2.67	2.62	2.67
BA-35	R perirhinal ctx	−86.6	6.00	5.78	5.72	5.69	5.67	1.64	1.58	1.59	1.66	1.67
BA-36	L parahippocampal ctx	−76.5	6.06	5.86	5.82	5.81	5.79	2.65	2.74	2.75	2.68	2.70
BA-36	R parahippocampal ctx	−67.6	5.96	5.74	5.71	5.67	5.64	1.70	1.73	1.71	1.71	1.73
BA-37	L fusiform gyrus	−37.3	5.93	5.76	5.69	5.61	5.61	2.41	2.43	2.45	2.51	2.52
BA-37	R fusiform gyrus	−35.2	5.99	5.73	5.71	5.66	5.61	1.83	1.91	1.93	1.95	2.00
BA-38	L temporopolar area	−55.9	6.22	6.00	5.99	5.99	5.91	2.69	2.80	2.85	2.86	2.86
BA-38	R temporopolar area	−52.8	6.08	5.91	5.88	5.81	5.77	1.84	1.88	1.86	1.93	1.92
BA-39	L angular gyrus	−35.2	5.96	5.80	5.79	5.75	5.72	2.58	2.63	2.68	2.65	2.70
BA-39	R angular gyrus	−34.7	5.98	5.72	5.64	5.63	5.59	2.01	2.06	2.04	2.09	2.06
BA-40	L supramarginal gyrus	−33.4	6.05	5.88	5.84	5.84	5.85	2.69	2.68	2.72	2.80	2.77
BA-40	R supramarginal gyrus	−31.5	6.02	5.82	5.75	5.73	5.71	1.79	1.78	1.79	1.87	1.90
BA-42	L prim. and auditory assoc. ctx	−45.2	6.03	5.92	5.89	5.89	5.82	2.75	2.80	2.78	2.86	2.83
BA-42	R prim. and auditory assoc. ctx	−38.5	6.05	5.82	5.79	5.83	5.77	1.75	1.81	1.80	1.91	1.79
BA-43	L subcentral area	−37.6	6.13	6.07	6.00	6.07	6.00	2.95	2.99	2.98	3.09	3.06
BA-43	R subcentral area	−41.2	6.04	5.80	5.82	5.84	5.74	1.78	1.80	1.86	1.86	1.86
BA-44	L pars opercularis	−43.7	6.14	6.07	5.98	6.00	5.99	2.81	2.83	2.85	2.90	2.93
BA-44	R pars opercularis	−44.0	6.20	5.95	5.92	5.85	5.83	1.65	1.65	1.68	1.69	1.73
BA-45	L pars triangularis	−40.3	6.15	6.00	5.94	5.91	5.93	2.67	2.75	2.75	2.74	2.76
BA-45	R pars triangularis	−39.5	6.27	6.01	5.97	5.92	5.94	1.72	1.66	1.80	1.77	1.76
BA-46	L dorsolateral prefrontal ctx	−36.4	6.20	6.04	5.99	5.92	5.93	2.62	2.68	2.68	2.66	2.69
BA-46	R dorsolateral prefrontal ctx	−37.4	6.27	6.00	5.95	5.94	5.89	1.72	1.76	1.75	1.78	1.83
BA-47	L inferior prefrontal gyrus	−48.6	6.12	5.97	5.89	5.88	5.86	2.47	2.50	2.58	2.57	2.57
BA-47	R inferior prefrontal gyrus	−47.4	6.30	6.00	5.96	5.92	5.88	1.91	1.95	1.99	2.01	2.02

Note: This table shows the DPF and PPF for several fNIRS-accessible Brodmann areas in the head in male subjects. The sensitivity of the region to fNIRS is provided in decibels (dB; 10×log 10) and indicates the fraction of photons normalized to the incidence power reaching this region from the scalp at a source–detector distance of 30 mm.

**Table 9:** Sensitivity, DPF, PPF of anatomical regions (females).

Region	Sens.	DPF (nm)					PPF (nm)				
		690	780	808	830	850	690	780	808	830	850
L banks of the superior temporal sulcus	−46.4	5.97	5.76	5.66	5.66	5.65	2.26	2.28	2.30	2.26	2.37
R banks of the superior temporal sulcus	−45.8	6.03	5.69	5.75	5.69	5.65	1.62	1.61	1.55	1.65	1.76
L caudal anterior cingulate	−79.0	6.18	6.00	5.94	5.92	5.88	2.12	2.18	2.21	2.21	2.43
R caudal anterior cingulate	−78.8	6.17	6.05	5.99	5.95	5.92	1.83	1.94	1.97	2.03	2.22
L caudal middle frontal	−37.3	6.14	5.94	5.91	5.92	5.90	2.40	2.44	2.49	2.48	2.54
R caudal middle frontal	−38.0	6.21	6.03	5.97	5.93	5.93	1.89	1.93	1.93	1.97	1.99
L cuneus	−52.7	5.98	5.71	5.60	5.55	5.52	1.59	1.80	1.81	2.00	2.00
R cuneus	−48.7	5.95	5.67	5.62	5.55	5.58	1.43	1.48	1.45	1.51	1.50
L entorhinal	−90.5	6.05	5.95	5.88	5.85	5.83	2.23	2.38	2.31	2.39	2.40
R entorhinal	−89.9	6.02	5.79	5.78	5.65	5.61	1.54	1.53	1.54	1.54	1.57
L frontal pole	−38.5	5.97	5.70	5.68	5.61	5.56	1.38	1.38	1.36	1.42	1.65
R frontal pole	−40.1	6.23	5.89	5.81	5.79	5.72	1.18	1.13	1.12	1.19	1.21
L fusiform	−55.6	6.04	5.84	5.76	5.73	5.69	2.27	2.34	2.32	2.35	2.37
R fusiform	−59.8	6.01	5.77	5.72	5.70	5.65	1.58	1.60	1.59	1.60	1.63
L inferior parietal	−29.8	5.98	5.73	5.69	5.67	5.60	2.01	2.01	2.02	2.08	2.20
R inferior parietal	−30.7	5.96	5.75	5.66	5.65	5.59	1.62	1.77	1.76	1.81	1.96
L inferior temporal	−35.1	6.03	5.85	5.80	5.79	5.74	2.30	2.43	2.42	2.39	2.46
R inferior temporal	−34.6	6.03	5.73	5.68	5.65	5.57	1.56	1.56	1.55	1.53	1.61
L insula	−62.0	6.15	5.98	5.94	5.91	5.91	2.47	2.55	2.59	2.57	2.61
R insula	−61.1	6.19	5.91	5.91	5.80	5.76	1.63	1.62	1.61	1.66	1.72
L isthmus cingulate	−88.0	5.96	5.77	5.73	5.65	5.60	2.04	2.07	2.06	2.09	2.16
R isthmus cingulate	−90.1	5.96	5.69	5.65	5.63	5.56	1.89	1.91	1.93	1.94	1.94
L lateral occipital	−27.7	6.02	5.74	5.69	5.64	5.61	1.79	1.84	1.85	1.84	1.92
R lateral occipital	−27.9	5.97	5.67	5.59	5.54	5.51	1.25	1.31	1.33	1.37	1.40
L lateral orbitofrontal	−64.1	6.01	5.83	5.82	5.76	5.70	2.03	2.07	2.15	2.11	2.15
R lateral orbitofrontal	−62.8	6.27	5.93	5.90	5.85	5.84	1.30	1.24	1.25	1.30	1.35
L lingual	−57.6	6.06	5.76	5.70	5.64	5.61	1.67	1.70	1.70	1.75	1.90
R lingual	−71.8	5.97	5.62	5.59	5.52	5.47	1.24	1.31	1.29	1.38	1.51
L medial orbitofrontal	−64.2	5.96	5.73	5.67	5.63	5.60	1.49	1.43	1.44	1.53	1.55
R medial orbitofrontal	−61.5	6.21	5.93	5.88	5.78	5.74	1.13	1.11	1.10	1.10	1.23
L middle temporal	−28.9	6.04	5.86	5.83	5.81	5.77	2.38	2.38	2.38	2.39	2.46
R middle temporal	−26.9	6.04	5.77	5.71	5.68	5.66	1.68	1.71	1.70	1.65	1.73
L paracentral	−53.3	6.06	5.88	5.89	5.83	5.80	2.35	2.35	2.39	2.47	2.51
R paracentral	−50.7	6.08	5.86	5.82	5.77	5.78	1.91	1.99	2.03	2.15	2.24
L parahippocampal	−85.4	6.02	5.83	5.77	5.73	5.74	2.22	2.32	2.26	2.38	2.47
R parahippocampal	−84.3	6.01	5.76	5.70	5.68	5.70	1.56	1.55	1.57	1.53	1.57
L pars opercularis	−42.8	6.12	5.97	5.94	5.90	5.89	2.42	2.56	2.59	2.53	2.61
R pars opercularis	−44.4	6.34	6.12	6.02	6.00	5.91	1.67	1.74	1.70	1.75	1.79
L pars orbitalis	−42.4	6.06	5.86	5.91	5.79	5.82	2.45	2.49	2.54	2.51	2.59
R pars orbitalis	−40.1	6.31	5.97	5.94	5.91	5.86	1.37	1.36	1.34	1.39	1.40
L pars triangularis	−36.9	6.17	5.97	5.93	5.85	5.89	2.36	2.43	2.47	2.46	2.50
R pars triangularis	−36.1	6.32	6.13	6.07	6.00	5.97	1.68	1.67	1.65	1.70	1.81
L pericalcarine	−45.5	6.00	5.71	5.66	5.62	5.57	1.83	1.89	1.86	1.94	1.96
R pericalcarine	−47.0	5.97	5.69	5.65	5.57	5.57	1.46	1.49	1.52	1.56	1.58
L postcentral	−30.8	6.10	5.90	5.89	5.84	5.83	2.29	2.37	2.37	2.34	2.42
R postcentral	−29.7	6.04	5.85	5.84	5.78	5.76	1.69	1.83	1.83	1.84	1.86
L posterior cingulate	−80.6	6.15	5.94	5.96	5.92	5.88	2.39	2.40	2.39	2.53	2.69
R posterior cingulate	−80.1	6.14	5.92	5.86	5.85	5.84	2.07	2.21	2.21	2.19	2.27
L precentral	−33.9	6.11	5.96	5.93	5.88	5.86	2.33	2.46	2.46	2.38	2.50
R precentral	−31.9	6.12	5.91	5.85	5.81	5.78	1.68	1.85	1.84	1.87	1.95
L precuneus	−58.6	5.96	5.72	5.68	5.61	5.57	1.91	1.93	1.96	1.97	1.97
R precuneus	−55.9	5.94	5.67	5.61	5.58	5.56	1.79	1.85	1.86	1.89	1.93
L rostral anterior cingulate	−71.1	5.99	5.81	5.74	5.67	5.62	1.54	1.57	1.60	1.60	1.81
R rostral anterior cingulate	−75.1	6.18	5.87	5.79	5.75	5.68	1.44	1.37	1.38	1.48	1.55
L rostral middle frontal	−31.6	6.08	5.88	5.80	5.77	5.78	2.00	2.00	2.06	2.02	2.13
R rostral middle frontal	−32.4	6.33	6.06	5.97	5.93	5.89	1.42	1.50	1.54	1.55	1.55
L superior frontal	−33.0	6.16	5.96	5.89	5.87	5.85	2.02	2.10	2.09	2.11	2.17
R superior frontal	−34.2	6.20	6.00	5.95	5.94	5.86	1.79	1.84	1.87	1.90	1.99
L superior parietal	−37.8	5.97	5.76	5.70	5.65	5.63	2.04	2.13	2.09	2.11	2.17
R superior parietal	−34.6	6.01	5.73	5.69	5.66	5.61	1.81	1.85	1.87	1.88	1.96
L superior temporal	−41.5	6.09	5.91	5.87	5.84	5.83	2.42	2.51	2.51	2.52	2.56
R superior temporal	−32.8	6.08	5.86	5.84	5.76	5.75	1.64	1.67	1.66	1.67	1.73
L supramarginal	−33.8	5.98	5.81	5.74	5.71	5.69	2.18	2.22	2.21	2.25	2.28
R supramarginal	−32.0	6.07	5.87	5.83	5.79	5.74	1.59	1.66	1.66	1.73	1.86
L temporal pole	−73.1	6.13	5.93	5.89	5.81	5.86	2.27	2.40	2.37	2.34	2.33
R temporal pole	−74.0	6.12	5.79	5.83	5.68	5.64	1.72	1.74	1.73	1.78	1.78
L transverse temporal	−64.9	6.08	5.91	5.86	5.83	5.84	2.76	2.71	2.71	2.84	2.80
R transverse temporal	−55.0	6.08	5.83	5.81	5.71	5.70	1.72	1.71	1.72	1.78	1.85

Note: This table shows the DPF and PPF for several fNIRS-accessible anatomical regions in the head in female subjects. The sensitivity of the region to fNIRS is provided in decibels (dB; 10×log 10) and indicates the fraction of photons normalized to the incidence power reaching this region from the scalp at a source–detector distance of 30 mm.

**Table 10:** Sensitivity, DPF, PPF of anatomical regions (males).

Region	Sens.	DPF (nm)					PPF (nm)				
		690	780	808	830	850	690	780	808	830	850
L banks of the superior temporal sulcus	−42.7	5.96	5.76	5.73	5.72	5.72	2.31	2.37	2.39	2.48	2.51
R banks of the superior temporal sulcus	−46.2	6.05	5.74	5.70	5.69	5.61	1.74	1.78	1.80	1.78	1.81
L caudal anterior cingulate	−80.1	6.26	6.04	5.99	5.96	5.90	2.52	2.52	2.53	2.47	2.50
R caudal anterior cingulate	−80.4	6.24	6.03	6.05	6.01	5.97	2.14	2.10	2.16	2.12	2.16
L caudal middle frontal	−36.3	6.17	5.99	5.97	5.98	5.97	2.80	2.86	2.89	2.80	2.83
R caudal middle frontal	−37.4	6.22	6.05	5.97	5.95	5.88	2.06	2.06	2.05	2.11	2.17
L cuneus	−53.3	5.97	5.77	5.70	5.68	5.65	2.60	2.64	2.67	2.59	2.66
R cuneus	−47.4	5.89	5.73	5.65	5.59	5.55	2.21	2.12	2.16	2.02	2.03
L entorhinal	−90.5	6.16	5.99	5.96	5.91	5.92	2.68	2.80	2.80	2.80	2.81
R entorhinal	−91.5	6.03	5.78	5.76	5.75	5.73	2.10	2.12	2.02	2.24	2.23
L frontal pole	−38.8	6.10	5.84	5.81	5.71	5.67	1.77	1.81	1.80	1.87	1.87
R frontal pole	−40.5	6.19	5.96	5.85	5.86	5.78	1.58	1.61	1.59	1.65	1.65
L fusiform	−51.4	6.03	5.80	5.79	5.75	5.75	2.62	2.64	2.65	2.68	2.72
R fusiform	−56.7	6.03	5.78	5.75	5.67	5.68	1.79	1.83	1.83	1.82	1.85
L inferior parietal	−29.6	5.98	5.79	5.77	5.73	5.70	2.61	2.60	2.61	2.65	2.65
R inferior parietal	−32.1	6.01	5.73	5.67	5.66	5.60	1.99	2.03	2.01	2.08	2.09
L inferior temporal	−37.7	6.05	5.86	5.78	5.76	5.75	2.56	2.44	2.46	2.48	2.49
R inferior temporal	−35.1	6.01	5.73	5.67	5.68	5.62	1.82	1.85	1.88	1.87	1.89
L insula	−2.3	6.15	6.02	5.99	5.96	5.93	2.70	2.78	2.81	2.82	2.84
R insula	−63.0	6.08	5.89	5.84	5.81	5.76	1.77	1.77	1.81	1.80	1.81
L isthmus cingulate	−89.7	5.99	5.81	5.74	5.73	5.74	2.60	2.59	2.62	2.62	2.56
R isthmus cingulate	−89.6	6.01	5.81	5.82	5.79	5.78	2.70	2.71	2.72	2.59	2.61
L lateral occipital	−27.1	6.03	5.87	5.82	5.80	5.75	2.62	2.51	2.55	2.70	2.73
R lateral occipital	−28.6	5.95	5.73	5.69	5.64	5.59	1.99	1.98	2.01	2.05	2.05
L lateral orbitofrontal	−60.9	6.12	5.95	5.91	5.88	5.84	2.21	2.21	2.25	2.23	2.24
R lateral orbitofrontal	−63.4	6.26	6.02	5.95	5.94	5.85	1.49	1.52	1.52	1.55	1.54
L lingual	−81.1	6.02	5.85	5.82	5.79	5.81	2.62	2.56	2.59	2.70	2.70
R lingual	−70.5	5.91	5.66	5.62	5.58	5.56	1.99	2.03	2.01	2.07	2.07
L medial orbitofrontal	−62.5	6.03	5.81	5.78	5.78	5.70	1.58	1.54	1.52	1.57	1.53
R medial orbitofrontal	−61.3	6.19	5.94	5.85	5.85	5.79	1.37	1.36	1.36	1.35	1.36
L middle temporal	−28.6	6.07	5.88	5.82	5.83	5.76	2.56	2.57	2.60	2.61	2.60
R middle temporal	−34.1	6.01	5.79	5.72	5.72	5.64	1.71	1.75	1.72	1.79	1.77
L paracentral	−51.9	6.09	5.98	5.99	5.93	5.90	2.64	2.67	2.69	2.69	2.72
R paracentral	−49.3	6.09	5.85	5.80	5.81	5.76	2.22	2.27	2.27	2.29	2.30
L parahippocampal	−86.8	6.03	5.85	5.77	5.76	5.75	2.64	2.64	2.67	2.60	2.61
R parahippocampal	−86.1	5.95	5.68	5.62	5.58	5.55	1.75	1.79	1.80	1.82	1.84
L pars opercularis	−42.7	6.15	5.94	5.90	5.90	5.87	2.60	2.74	2.74	2.69	2.76
R pars opercularis	−46.9	6.23	5.99	5.95	5.91	5.90	1.74	1.72	1.77	1.81	1.81
L pars orbitalis	−39.9	6.15	5.98	5.97	6.01	5.95	2.34	2.40	2.44	2.42	2.42
R pars orbitalis	−38.7	6.35	6.04	6.05	5.95	5.95	1.31	1.34	1.33	1.40	1.38
L pars triangularis	−36.8	6.12	6.00	5.91	5.93	5.88	2.66	2.79	2.77	2.75	2.78
R pars triangularis	−35.5	6.25	6.02	6.04	6.03	5.94	1.93	1.98	1.98	2.03	2.03
L pericalcarine	−47.3	5.99	5.80	5.75	5.69	5.67	2.57	2.49	2.51	2.42	2.45
R pericalcarine	−51.0	5.94	5.74	5.68	5.66	5.63	2.30	2.32	2.35	2.31	2.31
L postcentral	−30.6	6.13	6.00	5.93	5.94	5.93	2.74	2.78	2.80	2.77	2.79
R postcentral	−29.4	6.12	5.90	5.86	5.81	5.80	2.07	2.14	2.13	2.14	2.17
L posterior cingulate	−80.1	6.13	6.01	6.00	5.98	5.93	2.65	2.74	2.76	2.68	2.70
R posterior cingulate	−81.5	6.08	5.86	5.82	5.82	5.80	2.28	2.31	2.37	2.33	2.33
L precentral	−32.9	6.14	6.02	6.03	6.05	6.02	2.78	2.85	2.86	2.84	2.86
R precentral	−32.1	6.12	5.94	5.90	5.85	5.81	2.11	2.14	2.13	2.07	2.08
L precuneus	−56.4	6.00	5.91	5.87	5.85	5.83	2.72	2.70	2.71	2.67	2.65
R precuneus	−55.9	6.03	5.88	5.85	5.80	5.79	2.52	2.51	2.49	2.43	2.43
L rostral anterior cingulate	−74.9	6.10	5.86	5.82	5.76	5.71	2.11	2.11	2.16	2.11	2.10
R rostral anterior cingulate	−77.4	6.20	5.96	5.90	5.86	5.78	1.66	1.66	1.66	1.66	1.66
L rostral middle frontal	−30.7	6.15	6.00	5.98	5.96	5.93	2.47	2.47	2.45	2.44	2.45
R rostral middle frontal	−33.1	6.26	6.04	5.97	5.94	5.87	1.65	1.70	1.71	1.72	1.77
L superior frontal	−30.4	6.18	6.03	5.97	5.96	5.91	2.51	2.54	2.54	2.48	2.51
R superior frontal	−34.2	6.16	6.02	5.97	5.92	5.91	1.95	2.05	2.08	1.99	2.02
L superior parietal	−35.2	6.02	5.85	5.83	5.81	5.82	2.69	2.77	2.77	2.78	2.78
R superior parietal	−34.2	6.05	5.84	5.81	5.78	5.74	2.21	2.01	2.01	1.99	2.04
L superior temporal	−39.9	6.13	5.93	5.86	5.85	5.83	2.71	2.76	2.76	2.80	2.82
R superior temporal	−39.9	6.05	5.84	5.80	5.78	5.71	1.68	1.61	1.68	1.69	1.69
L supramarginal	−33.6	6.03	5.81	5.80	5.79	5.74	2.70	2.72	2.76	2.81	2.79
R supramarginal	−32.5	6.01	5.82	5.76	5.71	5.66	1.97	1.99	2.03	2.04	2.02
L temporal pole	−75.9	6.23	6.01	6.02	5.96	5.93	2.63	2.68	2.71	2.72	2.75
R temporal pole	−74.3	6.16	5.96	5.91	5.91	5.80	1.92	1.96	1.98	2.01	2.03
L transverse temporal	−65.1	6.05	5.90	5.86	5.84	5.82	2.72	2.74	2.77	2.72	2.74
R transverse temporal	−67.3	6.06	5.83	5.81	5.82	5.79	1.67	1.63	1.67	1.69	1.69

Note: This table shows the DPF and PPF for several fNIRS-accessible anatomical regions in the head of male subjects. The sensitivity of the region to fNIRS is provided in decibels (dB; 10×log 10) and indicates the fraction of photons normalized to the incidence power reaching this region from the scalp at a source–detector distance of 30 mm.

**Table 11:** Nearest 10-5 location for recording Brodmann areas (females).

BA	Region	Depth	Position 1		Position 2		Position 3	
		Med (min to max)	Name	Depth	Name	Depth	Name	Depth
BA-1	L prim. somatosensory ctx	17.74 (9.83 to 26.80)	C3h	18.83	C3	18.86	C1	21.91
BA-1	R prim. somatosensory ctx	16.66 (10.67 to 21.46)	C4	16.58	C4h	18.52	C2	21.39
BA-2	L prim. somatosensory ctx	19.51 (13.90 to 26.80)	C3	18.61	C3h	20.97	C1	22.91
BA-2	R prim. somatosensory ctx	18.68 (14.29 to 25.25)	C4	15.81	C4h	21.27	C2	22.61
BA-3	L prim. somatosensory ctx	21.04 (15.31 to 27.58)	FCC3h	22.31	C1	23.35	C3h	23.50
BA-3	R prim. somatosensory ctx	17.49 (14.38 to 24.37)	C4	18.17	C2	22.14	C2h	22.33
BA-4	L prim. motor ctx	17.23 (13.92 to 26.61)	FCC5h	19.14	C1h	19.40	FCC3	19.84
BA-4	R prim. motor ctx	18.42 (12.92 to 25.18)	C2h	20.02	FCC4h	23.31	C4h	23.65
BA-5	L somatosensory assoc. ctx	21.88 (17.56 to 28.54)	C1h	22.92	CCPz	25.65	Cz	28.59
BA-5	R somatosensory assoc. ctx	22.30 (17.25 to 29.13)	CCP2	22.58	CCP2h	22.69	C2h	26.14
BA-6	L motor and suppl. motor ctx	20.53 (16.11 to 25.98)	FCC1h	22.26	FCC1	22.58	FCCz	26.42
BA-6	R motor and suppl. motor ctx	18.57 (14.59 to 24.12)	FCC4	17.50	FCC2h	21.81	FCC2	22.36
BA-7	L somatosensory assoc. ctx	23.36 (19.07 to 29.61)	CP1	22.33	CP1h	23.50	CCP1h	24.14
BA-7	R somatosensory assoc. ctx	23.17 (19.08 to 30.78)	CP2	22.49	CP2h	22.60	CCP2h	24.08
BA-8	L includes frontal eye fields	17.65 (11.58 to 24.82)	F1h	18.42	FFC1h	19.25	FFC1	19.25
BA-8	R includes frontal eye fields	18.73 (12.52 to 23.75)	FC4h	19.60	FFC2h	20.49	FFC2	21.10
BA-9	L dorsolateral prefrontal ctx	20.25 (13.85 to 25.88)	F1	19.30	FCC3	23.50	FC3	24.01
BA-9	R dorsolateral prefrontal ctx	19.29 (14.34 to 25.82)	FC4	22.04	FCC4	22.51	FC4h	22.65
BA-10	L frontopolar area	19.11 (14.97 to 25.12)	F3	17.92	AFF1	23.95	AFF1h	24.01
BA-10	R frontopolar area	18.19 (15.34 to 23.34)	F4	17.58	AFF2	21.14	AFF2h	21.60
BA-11	L orbitofrontal area	33.41 (28.12 to 39.29)	AFp7	33.09	Fp1h	34.63	Fp1	36.82
BA-11	R orbitofrontal area	30.21 (25.89 to 38.14)	F6	25.38	Fp2	35.30	AFp8	37.84
BA-17	L prim. visual ctx (V1)	16.78 (11.62 to 26.78)	POO1	15.54	PO1	17.62	PO1h	18.74
BA-17	R prim. visual ctx (V1)	18.74 (14.12 to 25.58)	PO2	16.87	PO4h	17.11	PO2h	18.08
BA-18	L visual assoc. ctx (V2)	18.44 (15.39 to 24.39)	PPO3h	16.87	PPO1h	18.77	PO1h	20.92
BA-18	R visual assoc. ctx (V2)	18.54 (15.78 to 25.04)	PPO4h	16.71	PPO2	17.32	PPO2h	20.19
BA-19	L associative visual ctx (V3)	19.96 (16.78 to 24.85)	P1	20.43	CPP3	20.73	CPP5h	21.83
BA-19	R associative visual ctx (V3)	18.92 (16.35 to 24.77)	P4h	18.80	P2	20.44	P2h	22.73
BA-20	L inferior temporal gyrus	25.54 (21.70 to 34.18)	CCP5	27.47	T7h	31.55	C5	34.81
BA-20	R inferior temporal gyrus	25.99 (19.92 to 33.58)	CCP6	24.14	C6	27.86	T8h	30.48
BA-21	L middle temporal gyrus	21.60 (17.42 to 28.05)	CP5h	21.87	CCP5	22.68	C5	24.33
BA-21	R middle temporal gyrus	18.62 (13.02 to 25.89)	CCP6	17.52	C6	20.17	FCC6	23.26
BA-22	L superior temporal gyrus	24.08 (20.95 to 27.83)	CCP5h	23.16	C5h	27.15	FCC5h	30.81
BA-22	R superior temporal gyrus	23.02 (17.13 to 29.15)	CCP6h	21.63	FCC6h	26.27	C6h	27.08
BA-23	L ventral post. cingulate ctx	56.82 (46.48 to 63.40)	FCCz	46.02	Cz	46.94	FCC1h	47.40
BA-23	R ventral post. cingulate ctx	44.10 (35.34 to 51.29)	FCC2h	42.34	C2h	42.53	C2	43.82
BA-24	L ventral ant. cingulate ctx	47.76 (35.43 to 56.48)	FCC1h	40.09	FC1h	40.57	FCz	43.47
BA-24	R ventral ant. cingulate ctx	38.18 (28.41 to 45.94)	C6h	34.65	FC2h	38.13	FCC2h	38.81
BA-25	L subgenual ctx	57.24 (46.45 to 61.82)	F1	43.01	F1h	43.26	AFF1h	44.42
BA-25	R subgenual ctx	53.07 (27.08 to 61.68)	FFC4	33.41	FC6h	33.94	FC4	35.13
BA-28	L post. entorhinal ctx	44.83 (38.39 to 53.50)	FCC5	44.92	FTT7h	45.19	T7h	46.43
BA-28	R post. entorhinal ctx	42.91 (35.51 to 49.62)	FCC6	42.79	T8h	44.40	FTT8h	44.97
BA-29	L retrospleniendral cingular ctx	48.81 (39.72 to 55.55)	CPP1h	42.29	Pz	42.75	CPPz	43.08
BA-29	R retrosplenial cingular ctx	47.77 (40.83 to 53.92)	CPz	43.79	CPP2h	44.13	CP2h	44.69
BA-30	L part of cingular ctx	45.68 (40.97 to 51.42)	PPOz	39.63	P3h	41.55	P3	42.41
BA-30	R part of cingular ctx	44.24 (40.28 to 50.43)	P4h	40.28	P4	42.00	CPP6h	43.56
BA-33	L part of ant. cingulate gyrus	44.31 (33.02 to 53.70)	FC1h	38.46	FFC1	40.50	FFC1h	41.16
BA-33	R part of ant. cingulate gyrus	36.60 (22.79 to 41.50)	FC4	33.49	FC2h	38.00	FFC2h	38.33
BA-34	L ant. entorhinal ctx	59.50 (51.99 to 64.17)	T7h	47.81	FTT7h	47.88	FCC5	47.98
BA-34	R ant. entorhinal ctx	52.41 (45.77 to 58.64)	C6	45.97	FCC6	45.98	FTT8h	46.85
BA-35	L perirhinal ctx	50.42 (46.74 to 56.03)	CP5	47.13	CCP5	47.71	C5	48.48
BA-35	R perirhinal ctx	45.22 (38.15 to 54.14)	CCP6	44.12	CP6	44.86	C6	45.09
BA-36	L parahippocampal ctx	41.38 (38.26 to 46.72)	TP7h	39.01	CP5	41.41	CCP5	43.67
BA-36	R parahippocampal ctx	38.09 (31.44 to 44.12)	CP6	32.80	CCP6	39.48	C6	40.59
BA-37	L fusiform gyrus	17.22 (11.79 to 24.04)	CPP5h	17.98	CPP5	18.82	CP5	19.83
BA-37	R fusiform gyrus	16.62 (11.57 to 23.33)	CP6	16.59	CP6h	18.37	CPP6h	19.85
BA-38	L temporopolar area	32.44 (27.60 to 41.44)	FC5	31.97	FT7h	34.97	FTT7h	37.19
BA-38	R temporopolar area	29.50 (22.72 to 36.56)	FC6	29.13	FCC6	29.72	FTT8h	34.47
BA-39	L angular gyrus	18.91 (14.61 to 26.24)	CP3	19.53	CPP3	19.58	CP3h	21.81
BA-39	R angular gyrus	18.40 (12.94 to 25.00)	CP4h	18.94	CP4	19.63	CPP4	19.91
BA-40	L supramarginal gyrus	20.14 (16.16 to 25.98)	CCP3	19.49	CCP3h	22.03	C3h	23.20
BA-40	R supramarginal gyrus	20.10 (15.64 to 26.12)	CCP4	18.71	CCP4h	22.57	C4h	22.84
BA-42	L prim. and auditory assoc. ctx	24.85 (16.20 to 29.57)	C5h	24.54	CCP5h	25.97	FCC5h	28.06
BA-42	R prim. and auditory assoc. ctx	20.78 (12.53 to 27.26)	C6h	21.51	CCP6h	22.05	C4	23.10
BA-43	L subcentral area	14.98 (8.86 to 24.00)	C5h	15.33	FCC5h	16.68	FCC3	21.84
BA-43	R subcentral area	12.37 (5.01 to 20.88)	C6h	15.14	C4	17.10	FCC4	19.85
BA-44	L pars opercularis	18.53 (13.47 to 25.58)	FC5h	19.22	FCC5h	19.58	FC3	22.17
BA-44	R pars opercularis	17.85 (8.33 to 25.40)	FCC6h	18.83	FC6h	18.89	FC4	21.75
BA-45	L pars triangularis	16.27 (12.91 to 24.41)	FC5h	16.64	FFC5h	18.03	FC3	20.59
BA-45	R pars triangularis	14.26 (6.86 to 22.95)	FC6h	15.77	FFC6h	16.57	FC4	17.23
BA-46	L dorsolateral prefrontal ctx	17.54 (13.29 to 24.16)	FFC3	17.96	FFC5h	18.82	FC3	19.46
BA-46	R dorsolateral prefrontal ctx	16.75 (12.02 to 23.75)	FFC6h	17.15	FFC4	18.62	FC4	19.60
BA-47	L inferior prefrontal gyrus	30.20 (25.36 to 37.90)	FFC5	24.76	FC5	26.12	FFC5h	29.52
BA-47	R inferior prefrontal gyrus	29.08 (19.95 to 35.88)	FFC6h	26.87	FC6h	27.59	FC6	31.25

Note: This table provides the depth (median and range) of several Brodmann areas in female subjects. For each region, the nearest three 10-5 coordinate positions and the depth of the region to this position is provided as guidance for the placement of fNIRS sensors.

**Table 12:** Nearest 10-5 location for recording Brodmann areas (males).

BA	Region	Depth	Position 1		Position 2		Position 3	
		Med (min to max)	Name	Depth	Name	Depth	Name	Depth
BA-1	L prim. somatosensory ctx	17.74 (9.83 to 26.80)	C3h	18.83	C3	18.86	C1	21.91
BA-1	R prim. somatosensory ctx	16.66 (10.67 to 21.46)	C4	16.58	C4h	18.52	C2	21.39
BA-2	L prim. somatosensory ctx	19.51 (13.90 to 26.80)	C3	18.61	C3h	20.97	C1	22.91
BA-2	R prim. somatosensory ctx	18.68 (14.29 to 25.25)	C4	15.81	C4h	21.27	C2	22.61
BA-3	L prim. somatosensory ctx	21.04 (15.31 to 27.58)	FCC3h	22.31	C1	23.35	C3h	23.50
BA-3	R prim. somatosensory ctx	17.49 (14.38 to 24.37)	C4	18.17	C2	22.14	C2h	22.33
BA-4	L prim. motor ctx	17.23 (13.92 to 26.61)	FCC5h	19.14	C1h	19.40	FCC3	19.84
BA-4	R prim. motor ctx	18.42 (12.92 to 25.18)	C2h	20.02	FCC4h	23.31	C4h	23.65
BA-5	L somatosensory assoc. ctx	21.88 (17.56 to 28.54)	C1h	22.92	CCPz	25.65	Cz	28.59
BA-5	R somatosensory assoc. ctx	22.30 (17.25 to 29.13)	CCP2	22.58	CCP2h	22.69	C2h	26.14
BA-6	L motor and suppl. motor ctx	20.53 (16.11 to 25.98)	FCC1h	22.26	FCC1	22.58	FCCz	26.42
BA-6	R motor and suppl. motor ctx	18.57 (14.59 to 24.12)	FCC4	17.50	FCC2h	21.81	FCC2	22.36
BA-7	L somatosensory assoc. ctx	23.36 (19.07 to 29.61)	CP1	22.33	CP1h	23.50	CCP1h	24.14
BA-7	R somatosensory assoc. ctx	23.17 (19.08 to 30.78)	CP2	22.49	CP2h	22.60	CCP2h	24.08
BA-8	L includes frontal eye fields	17.65 (11.58 to 24.82)	F1h	18.42	FFC1h	19.25	FFC1	19.25
BA-8	R includes frontal eye fields	18.73 (12.52 to 23.75)	FC4h	19.60	FFC2h	20.49	FFC2	21.10
BA-9	L dorsolateral prefrontal ctx	20.25 (13.85 to 25.88)	F1	19.30	FCC3	23.50	FC3	24.01
BA-9	R dorsolateral prefrontal ctx	19.29 (14.34 to 25.82)	FC4	22.04	FCC4	22.51	FC4h	22.65
BA-10	L frontopolar area	19.11 (14.97 to 25.12)	F3	17.92	AFF1	23.95	AFF1h	24.01
BA-10	R frontopolar area	18.19 (15.34 to 23.34)	F4	17.58	AFF2	21.14	AFF2h	21.60
BA-11	L orbitofrontal area	33.41 (28.12 to 39.29)	AFp7	33.09	Fp1h	34.63	Fp1	36.82
BA-11	R orbitofrontal area	30.21 (25.89 to 38.14)	F6	25.38	Fp2	35.30	AFp8	37.84
BA-17	L prim. visual ctx (V1)	16.78 (11.62 to 26.78)	POO1	15.54	PO1	17.62	PO1h	18.74
BA-17	R prim. visual ctx (V1)	18.74 (14.12 to 25.58)	PO2	16.87	PO4h	17.11	PO2h	18.08
BA-18	L visual assoc. ctx (V2)	18.44 (15.39 to 24.39)	PPO3h	16.87	PPO1h	18.77	PO1h	20.92
BA-18	R visual assoc. ctx (V2)	18.54 (15.78 to 25.04)	PPO4h	16.71	PPO2	17.32	PPO2h	20.19
BA-19	L associative visual ctx (V3)	19.96 (16.78 to 24.85)	P1	20.43	CPP3	20.73	CPP5h	21.83
BA-19	R associative visual ctx (V3)	18.92 (16.35 to 24.77)	P4h	18.80	P2	20.44	P2h	22.73
BA-20	L inferior temporal gyrus	25.54 (21.70 to 34.18)	CCP5	27.47	T7h	31.55	C5	34.81
BA-20	R inferior temporal gyrus	25.99 (19.92 to 33.58)	CCP6	24.14	C6	27.86	T8h	30.48
BA-21	L middle temporal gyrus	21.60 (17.42 to 28.05)	CP5h	21.87	CCP5	22.68	C5	24.33
BA-21	R middle temporal gyrus	18.62 (13.02 to 25.89)	CCP6	17.52	C6	20.17	FCC6	23.26
BA-22	L superior temporal gyrus	24.08 (20.95 to 27.83)	CCP5h	23.16	C5h	27.15	FCC5h	30.81
BA-22	R superior temporal gyrus	23.02 (17.13 to 29.15)	CCP6h	21.63	FCC6h	26.27	C6h	27.08
BA-23	L ventral post. cingulate ctx	56.82 (46.48 to 63.40)	FCCz	46.02	Cz	46.94	FCC1h	47.40
BA-23	R ventral post. cingulate ctx	44.10 (35.34 to 51.29)	FCC2h	42.34	C2h	42.53	C2	43.82
BA-24	L ventral ant. cingulate ctx	47.76 (35.43 to 56.48)	FCC1h	40.09	FC1h	40.57	FCz	43.47
BA-24	R ventral ant. cingulate ctx	38.18 (28.41 to 45.94)	C6h	34.65	FC2h	38.13	FCC2h	38.81
BA-25	L subgenual ctx	57.24 (46.45 to 61.82)	F1	43.01	F1h	43.26	AFF1h	44.42
BA-25	R subgenual ctx	53.07 (27.08 to 61.68)	FFC4	33.41	FC6h	33.94	FC4	35.13
BA-28	L post. entorhinal ctx	44.83 (38.39 to 53.50)	FCC5	44.92	FTT7h	45.19	T7h	46.43
BA-28	R post. entorhinal ctx	42.91 (35.51 to 49.62)	FCC6	42.79	T8h	44.40	FTT8h	44.97
BA-29	L retrospleniendral cingular ctx	48.81 (39.72 to 55.55)	CPP1h	42.29	Pz	42.75	CPPz	43.08
BA-29	R retrosplenial cingular ctx	47.77 (40.83 to 53.92)	CPz	43.79	CPP2h	44.13	CP2h	44.69
BA-30	L part of cingular ctx	45.68 (40.97 to 51.42)	PPOz	39.63	P3h	41.55	P3	42.41
BA-30	R part of cingular ctx	44.24 (40.28 to 50.43)	P4h	40.28	P4	42.00	CPP6h	43.56
BA-33	L part of ant. cingulate gyrus	44.31 (33.02 to 53.70)	FC1h	38.46	FFC1	40.50	FFC1h	41.16
BA-33	R part of ant. cingulate gyrus	36.60 (22.79 to 41.50)	FC4	33.49	FC2h	38.00	FFC2h	38.33
BA-34	L ant. entorhinal ctx	59.50 (51.99 to 64.17)	T7h	47.81	FTT7h	47.88	FCC5	47.98
BA-34	R ant. entorhinal ctx	52.41 (45.77 to 58.64)	C6	45.97	FCC6	45.98	FTT8h	46.85
BA-35	L perirhinal ctx	50.42 (46.74 to 56.03)	CP5	47.13	CCP5	47.71	C5	48.48
BA-35	R perirhinal ctx	45.22 (38.15 to 54.14)	CCP6	44.12	CP6	44.86	C6	45.09
BA-36	L parahippocampal ctx	41.38 (38.26 to 46.72)	TP7h	39.01	CP5	41.41	CCP5	43.67
BA-36	R parahippocampal ctx	38.09 (31.44 to 44.12)	CP6	32.80	CCP6	39.48	C6	40.59
BA-37	L fusiform gyrus	17.22 (11.79 to 24.04)	CPP5h	17.98	CPP5	18.82	CP5	19.83
BA-37	R fusiform gyrus	16.62 (11.57 to 23.33)	CP6	16.59	CP6h	18.37	CPP6h	19.85
BA-38	L temporopolar area	32.44 (27.60 to 41.44)	FC5	31.97	FT7h	34.97	FTT7h	37.19
BA-38	R temporopolar area	29.50 (22.72 to 36.56)	FC6	29.13	FCC6	29.72	FTT8h	34.47
BA-39	L angular gyrus	18.91 (14.61 to 26.24)	CP3	19.53	CPP3	19.58	CP3h	21.81
BA-39	R angular gyrus	18.40 (12.94 to 25.00)	CP4h	18.94	CP4	19.63	CPP4	19.91
BA-40	L supramarginal gyrus	20.14 (16.16 to 25.98)	CCP3	19.49	CCP3h	22.03	C3h	23.20
BA-40	R supramarginal gyrus	20.10 (15.64 to 26.12)	CCP4	18.71	CCP4h	22.57	C4h	22.84
BA-42	L prim. and auditory assoc. ctx	24.85 (16.20 to 29.57)	C5h	24.54	CCP5h	25.97	FCC5h	28.06
BA-42	R prim. and auditory assoc. ctx	20.78 (12.53 to 27.26)	C6h	21.51	CCP6h	22.05	C4	23.10
BA-43	L subcentral area	14.98 (8.86 to 24.00)	C5h	15.33	FCC5h	16.68	FCC3	21.84
BA-43	R subcentral area	12.37 (5.01 to 20.88)	C6h	15.14	C4	17.10	FCC4	19.85
BA-44	L pars opercularis	18.53 (13.47 to 25.58)	FC5h	19.22	FCC5h	19.58	FC3	22.17
BA-44	R pars opercularis	17.85 (8.33 to 25.40)	FCC6h	18.83	FC6h	18.89	FC4	21.75
BA-45	L pars triangularis	16.27 (12.91 to 24.41)	FC5h	16.64	FFC5h	18.03	FC3	20.59
BA-45	R pars triangularis	14.26 (6.86 to 22.95)	FC6h	15.77	FFC6h	16.57	FC4	17.23
BA-46	L dorsolateral prefrontal ctx	17.54 (13.29 to 24.16)	FFC3	17.96	FFC5h	18.82	FC3	19.46
BA-46	R dorsolateral prefrontal ctx	16.75 (12.02 to 23.75)	FFC6h	17.15	FFC4	18.62	FC4	19.60
BA-47	L inferior prefrontal gyrus	30.20 (25.36 to 37.90)	FFC5	24.76	FC5	26.12	FFC5h	29.52
BA-47	R inferior prefrontal gyrus	29.08 (19.95 to 35.88)	FFC6h	26.87	FC6h	27.59	FC6	31.25

Note: This table provides the depth (median and range) of several Brodmann areas in male subjects. For each region, the nearest three 10-5 coordinate positions and the depth of the region to this position is provided as guidance for the placement of fNIRS sensors.

**Table 13:** Nearest 10-5 location for recording anatomical areas (females).

Region	Depth	Position 1		Position 2		Position 3	
	Med (min to max)	Name	Depth	Name	Depth	Name	Depth
L banks of the superior temporal sulcus	23.21 (16.11 to 29.05)	CP5h	24.15	CP5	24.65	CCP5	25.29
R banks of the superior temporal sulcus	23.56 (16.16 to 30.42)	CCP6h	23.27	CCP6	23.28	CP6h	24.67
L caudal anterior cingulate	39.28 (33.20 to 47.03)	FC1h	39.75	FFC1h	40.09	FFC1	40.42
R caudal anterior cingulate	39.95 (34.10 to 46.30)	FFC2h	39.84	FC2h	40.56	FCz	41.90
L caudal middle frontal	20.16 (14.04 to 26.27)	FC3h	19.78	FCC3h	21.65	FC3	25.11
R caudal middle frontal	19.82 (13.18 to 25.70)	FC2	20.11	FCC2	21.38	FC4h	22.56
L cuneus	28.23 (22.19 to 34.96)	PPO1h	26.70	PPOz	26.84	P1h	29.63
R cuneus	27.75 (14.83 to 34.30)	PPO2h	24.58	PPOz	27.04	Pz	28.87
L entorhinal	41.23 (33.56 to 44.60)	T7h	44.20	T7	44.97	FTT7h	45.15
R entorhinal	40.97 (32.97 to 45.95)	T8h	44.42	FTT8h	45.18	T8	46.69
L frontal pole	14.67 (10.80 to 19.21)	AFp1	14.31	AFp3h	15.06	Fp1h	16.14
R frontal pole	14.63 (12.08 to 20.75)	AFp4h	15.14	AFp2h	15.57	Fp2h	16.44
L fusiform	31.96 (28.22 to 39.27)	P5	29.54	CPP5	33.07	T7h	35.53
R fusiform	32.62 (27.68 to 40.28)	CPP6	31.97	T8h	35.69	CP6	36.44
L inferior parietal	18.27 (14.95 to 21.71)	CPP3h	18.04	CP3h	19.44	CPP3	20.81
R inferior parietal	17.98 (14.16 to 23.42)	CP4h	18.51	CP4	20.01	CPP4	20.12
L inferior temporal	19.80 (15.60 to 25.35)	CP5	18.76	CPP5	19.50	T7h	26.28
R inferior temporal	18.96 (14.27 to 25.30)	CP6	18.36	CPP6	18.78	T8h	26.15
L insula	35.28 (30.83 to 41.73)	FCC5	34.27	FCC5h	35.36	C5h	38.60
R insula	33.90 (26.95 to 40.54)	FCC6h	33.80	FCC6	33.84	C6h	36.07
L isthmus cingulate	53.73 (48.46 to 59.82)	CPP1h	45.46	CP1h	46.50	CPz	46.86
R isthmus cingulate	53.32 (45.89 to 59.62)	CPPz	46.69	CPz	46.70	CPP6h	47.21
L lateral occipital	14.29 (11.12 to 21.28)	PPO3h	15.64	P5h	18.67	CPP5h	22.28
R lateral occipital	14.16 (11.54 to 21.02)	PPO4	15.58	PPO4h	16.04	P6h	17.69
L lateral orbitofrontal	33.21 (28.82 to 42.25)	AFp7	34.60	FFC5	34.91	FC5	35.63
R lateral orbitofrontal	33.93 (29.23 to 41.80)	FFC6h	33.96	FFC6	36.12	FC6	37.89
L lingual	40.05 (33.31 to 47.05)	POO1h	31.42	POz	34.42	PPO5h	40.61
R lingual	40.64 (35.95 to 46.43)	PPO6h	39.09	P6h	42.05	CPP6h	43.89
L medial orbitofrontal	36.25 (31.39 to 43.95)	Fp1h	34.31	Fp1	39.01	AFp7	44.25
R medial orbitofrontal	37.52 (31.86 to 42.82)	AFp4h	32.85	Fp2h	34.98	Fp2	39.41
L middle temporal	15.91 (12.21 to 21.67)	CP5	15.80	CCP5	20.30	C5	20.61
R middle temporal	15.29 (9.52 to 21.80)	CP6h	14.71	CCP6	17.19	C6	18.61
L paracentral	31.27 (26.19 to 37.99)	Cz	29.01	CCP1h	30.74	C1h	33.38
R paracentral	31.32 (25.47 to 37.74)	CCPz	27.99	Cz	28.07	C2h	38.65
L parahippocampal	48.29 (44.53 to 52.38)	TTP7h	43.48	CP5	44.23	TP7h	45.30
R parahippocampal	47.74 (39.64 to 52.87)	T8h	43.22	CCP6	43.24	CP6	44.06
L pars opercularis	21.83 (17.86 to 28.37)	FC5h	22.75	FC3	23.87	FC5	24.53
R pars opercularis	22.30 (16.78 to 29.90)	FCC6h	23.33	FC6h	23.84	FC4	24.75
L pars orbitalis	18.73 (13.61 to 29.05)	F5	19.09	FFC5	20.11	FC5	20.90
R pars orbitalis	18.34 (13.32 to 28.56)	F6h	18.34	F6	19.33	FFC6	19.60
L pars triangularis	17.46 (14.39 to 24.87)	FFC5	17.98	FC5h	19.21	FFC5h	20.92
R pars triangularis	17.34 (12.67 to 26.09)	FC6h	18.01	FFC4	19.73	FFC6h	20.12
L pericalcarine	28.08 (23.56 to 38.01)	PO1	23.55	PO1h	25.51	PPO1h	31.08
R pericalcarine	32.05 (21.59 to 39.28)	PO2	24.32	PPO2	31.93	PPO2h	34.21
L postcentral	18.19 (14.12 to 26.28)	C3	18.81	C3h	19.92	C5h	20.55
R postcentral	17.72 (13.57 to 24.70)	C4	18.07	C4h	19.98	C2	21.34
L posterior cingulate	44.62 (39.13 to 50.33)	FCC1h	42.71	C1h	43.63	C1	44.69
R posterior cingulate	43.89 (35.48 to 50.37)	FCC2h	42.31	C2h	42.74	FCCz	43.52
L precentral	20.54 (15.71 to 26.01)	FCC5h	20.53	FCC3	20.84	FCC3h	22.12
R precentral	19.80 (15.05 to 26.21)	FCC4	19.33	FCC4h	21.07	FCC2	22.86
L precuneus	35.94 (29.59 to 42.34)	CP1h	33.50	CPP1h	33.92	P1h	34.74
R precuneus	36.48 (31.00 to 41.39)	CPz	32.01	CPPz	32.40	CPP2h	34.28
L rostral anterior cingulate	36.77 (31.08 to 44.50)	AFF1	35.23	AFF1h	35.68	AF3	37.73
R rostral anterior cingulate	40.84 (34.05 to 47.05)	AFF2h	37.10	AFF2	37.16	AFFz	37.63
L rostral middle frontal	16.65 (12.87 to 21.98)	FFC3	18.34	F3h	19.18	FFC3h	19.42
R rostral middle frontal	17.44 (13.68 to 22.37)	FFC4	18.91	FFC4h	19.49	F4h	19.83
L superior frontal	20.27 (15.51 to 27.77)	FFC1	20.91	FC1h	22.51	FCC1h	24.18
R superior frontal	21.65 (17.08 to 27.22)	FFC2h	21.40	FC2h	22.66	FCC2h	23.12
L superior parietal	21.50 (16.07 to 28.94)	CCP1	22.65	CP1	22.84	CCP3h	26.16
R superior parietal	21.26 (16.90 to 27.98)	CP2h	20.79	CCP2	23.82	CP2	23.85
L superior temporal	22.33 (18.26 to 27.88)	CCP5h	24.11	C5	24.33	FCC5	24.37
R superior temporal	21.82 (13.63 to 28.74)	C6h	19.10	FCC6	24.31	C6	24.56
L supramarginal	19.46 (16.28 to 24.64)	CCP3	19.85	C3	20.09	CCP5h	25.75
R supramarginal	18.97 (12.41 to 26.09)	C4	19.15	CCP4	19.43	CCP6h	25.53
L temporal pole	35.47 (27.47 to 42.46)	FFT7h	36.07	FT7h	36.68	FTT7h	38.98
R temporal pole	35.78 (25.70 to 43.27)	FT8h	36.88	FC6	38.00	FTT8h	40.29
L transverse temporal	32.22 (28.60 to 36.76)	C5h	32.05	C5	32.85	CCP5h	35.98
R transverse temporal	30.89 (23.11 to 39.98)	FCC6h	26.28	C6h	30.59	CCP6h	35.07

Note: This table provides the depth (median and range) of several anatomical regions in female subjects. For each region, the nearest three 10-5 coordinate positions and the depth of the region to this position is provided as guidance for the placement of fNIRS sensors.

**Table 14:** Nearest 10-5 location for recording anatomical regions (males).

Region	Depth	Position 1		Position 2		Position 3	
	Med (min to max)	Name	Depth	Name	Depth	Name	Depth
L banks of the superior temporal sulcus	21.14 (11.90 to 27.00)	CP5h	22.06	CCP5	22.16	CP5	23.25
R banks of the superior temporal sulcus	24.82 (14.22 to 30.44)	CCP6h	23.45	CCP6	25.09	CP6h	25.12
L caudal anterior cingulate	40.99 (28.19 to 46.86)	FC1h	40.37	FFC1	40.98	FFC1h	41.88
R caudal anterior cingulate	40.85 (30.59 to 46.40)	FFC2h	40.65	F2h	41.09	FC2h	41.50
L caudal middle frontal	20.58 (11.99 to 26.63)	FC3h	19.18	FCC3h	20.47	FC3	25.09
R caudal middle frontal	20.68 (11.36 to 24.36)	FCC2	19.75	FC4h	21.57	FCC4h	22.44
L cuneus	28.89 (23.94 to 36.05)	POz	27.02	PPOz	29.14	PPO1h	30.31
R cuneus	25.89 (17.16 to 34.01)	POz	23.80	PPOz	27.69	PPO2h	27.89
L entorhinal	39.86 (30.13 to 43.65)	FTT7h	44.17	T7	44.39	FTT7	45.12
R entorhinal	39.46 (25.51 to 45.07)	FTT8h	45.25	T8	45.94	FTT8	45.99
L frontal pole	14.79 (11.31 to 21.25)	AFp3h	14.50	AFp1	15.08	Fp1h	16.94
R frontal pole	15.58 (12.27 to 22.68)	AFp2	15.39	AFp4h	15.55	Fp2h	17.47
L fusiform	32.59 (28.25 to 36.92)	P5	27.23	CPP5	32.60	TP7h	37.36
R fusiform	34.15 (27.93 to 37.82)	P6	30.32	T8h	36.96	CP6	38.16
L inferior parietal	18.17 (13.25 to 22.86)	CPP3h	17.90	CPP3	19.44	CPP5h	23.46
R inferior parietal	17.88 (13.18 to 22.46)	CPP4	19.32	CP4	20.07	CPP6h	22.35
L inferior temporal	20.37 (16.54 to 28.07)	CPP5	20.29	TP7h	21.79	TTP7h	22.41
R inferior temporal	20.19 (13.89 to 27.26)	CPP6	18.61	CP6	19.28	T8h	26.15
L insula	35.89 (30.59 to 40.19)	FCC5	34.43	FCC5h	36.74	C5h	39.99
R insula	36.18 (29.30 to 40.25)	FCC6	34.77	FC6	35.31	C6h	39.65
L isthmus cingulate	54.38 (47.55 to 59.31)	CPP1h	46.37	P1h	46.79	CP1h	47.23
R isthmus cingulate	54.63 (47.31 to 60.58)	CPP2h	46.44	CPPz	47.31	Pz	47.63
L lateral occipital	14.08 (11.73 to 19.74)	PPO5h	15.27	P5h	18.50	P5	18.72
R lateral occipital	14.07 (11.69 to 18.85)	PPO4h	16.01	PPO4	16.63	P6h	18.02
L lateral orbitofrontal	33.40 (30.05 to 37.97)	F5	32.91	FFC5	34.98	AFp7	35.56
R lateral orbitofrontal	34.17 (29.27 to 39.09)	FFC6h	34.25	F6	34.41	FFC6	35.79
L lingual	41.60 (35.07 to 46.59)	P5h	43.98	P5	44.78	CPP5	45.74
R lingual	41.40 (37.28 to 46.82)	POz	38.42	P6h	43.35	CPP6h	43.91
L medial orbitofrontal	37.24 (30.88 to 43.72)	Fpz	33.39	Fp1h	36.19	Fp1	40.68
R medial orbitofrontal	38.62 (31.32 to 42.93)	Fpz	32.72	Fp2h	35.50	Fp2	39.94
L middle temporal	16.32 (12.36 to 20.13)	CP5	15.58	FTT7h	20.74	CCP5	21.14
R middle temporal	17.05 (9.74 to 20.93)	CCP6	19.13	C6	19.72	FTT8h	22.50
L paracentral	31.12 (20.90 to 35.62)	Cz	28.27	CCP1h	30.65	C1h	32.18
R paracentral	32.06 (22.04 to 41.07)	CCPz	27.17	Cz	28.14	C2h	38.24
L parahippocampal	48.31 (43.27 to 53.86)	CP5	44.20	TTP7h	44.84	TP7h	45.61
R parahippocampal	49.41 (36.90 to 53.63)	T8h	44.15	CP6	44.80	TTP8h	45.03
L pars opercularis	22.15 (15.67 to 27.38)	FC3	22.65	FC5h	23.34	FC5	24.43
R pars opercularis	24.02 (16.57 to 28.39)	FCC4	24.70	FC6h	24.70	FC4	25.26
L pars orbitalis	17.71 (13.52 to 23.96)	F5	17.57	FFC5	19.26	F7h	19.48
R pars orbitalis	17.38 (13.36 to 23.16)	F6h	17.51	F6	18.16	FFC6	18.80
L pars triangularis	18.19 (13.52 to 24.03)	FC5h	17.91	FFC5	19.05	FFC5h	21.84
R pars triangularis	17.05 (10.76 to 21.18)	FC6h	17.62	FFC6	18.68	FFC6h	20.30
L pericalcarine	31.03 (24.03 to 37.99)	POO1	24.55	PO1	27.94	PO1h	28.46
R pericalcarine	31.31 (23.03 to 38.29)	PO2h	26.53	PO2	27.66	PPO2	35.70
L postcentral	18.34 (10.85 to 21.43)	C3	18.69	C3h	18.83	C5h	20.26
R postcentral	18.67 (11.33 to 22.23)	C4	17.89	C4h	19.57	CCP2	21.78
L posterior cingulate	45.87 (33.51 to 51.01)	FCC1h	42.41	C1h	42.59	C1	44.66
R posterior cingulate	45.78 (33.09 to 50.59)	FCCz	43.05	C2h	43.55	FCC2h	43.67
L precentral	20.42 (11.63 to 23.83)	FCC3	19.95	FCC5h	19.95	C1	21.99
R precentral	20.83 (12.58 to 24.50)	FCC4	19.43	C2h	21.33	C2	22.50
L precuneus	36.38 (31.31 to 41.92)	CP1h	32.28	P1h	33.16	CPP1h	34.28
R precuneus	37.06 (31.98 to 40.83)	CPPz	31.99	Pz	33.27	P2h	34.94
L rostral anterior cingulate	39.17 (34.16 to 44.98)	AFF1	37.25	AFF1h	38.16	AF3	39.13
R rostral anterior cingulate	41.57 (34.99 to 48.21)	AFF2h	38.32	AFF2	38.99	AFFz	39.26
L rostral middle frontal	16.12 (11.35 to 20.22)	F3	17.77	FFC3	18.04	FFC3h	18.23
R rostral middle frontal	17.12 (11.97 to 21.71)	FFC4	19.33	FC4	19.48	FFC4h	19.65
L superior frontal	21.12 (10.86 to 24.22)	FFC1h	19.38	FC1h	21.49	FCC1h	22.69
R superior frontal	21.73 (12.48 to 26.70)	FFC2h	21.41	FC2h	22.26	FCC2h	22.55
L superior parietal	20.93 (16.82 to 25.99)	CP1	21.13	CPP1	22.33	CCP1	22.53
R superior parietal	20.75 (16.19 to 25.34)	CPP2h	20.54	CP2h	21.07	CP2	23.20
L superior temporal	22.57 (18.86 to 26.22)	FCC5	23.19	C5	23.55	CCP5h	24.95
R superior temporal	22.91 (16.21 to 27.73)	FCC6	23.18	CCP6h	23.36	C6	25.65
L supramarginal	19.61 (15.15 to 24.27)	CCP3	19.70	C5h	20.75	CCP5h	25.09
R supramarginal	20.28 (13.41 to 24.47)	C4	19.41	CCP4	19.68	CCP6h	27.32
L temporal pole	35.35 (25.47 to 41.75)	FT7	37.58	FT7h	38.97	FTT7	40.60
R temporal pole	36.35 (27.70 to 41.17)	FFT8h	37.00	FT8h	39.84	FTT8h	41.67
L transverse temporal	33.52 (23.75 to 37.96)	C5	32.11	C5h	33.11	CCP5h	37.45
R transverse temporal	34.22 (24.65 to 38.81)	C6	32.98	C6h	33.67	CCP6h	38.18

Note: This table provides the depth (median and range) of several anatomical regions in male subjects. For each region, the nearest three 10-5 coordinate positions and the depth of the region to this position is provided as guidance for the placement of fNIRS sensors.

**Table 15:** Generalized equation for DPF for 10-5 locations.

Name	Beta values							
	$\beta_{0}$	$\beta_{1}$	$\beta_{2}$	$\beta_{3}$	$\beta_{4}$	$\beta_{5}$	$\beta_{6}$	$R^{2}$
Fp1	$7.7\times 10^{-4}$	$-9.3\times 10^{-2}$	$-3.5\times 10^{-4}$	$-1.5\times 10^{-3}$	$1.6\times 10^{-1}$	$2.7\times 10^{-7}$	$-1.6\times 10^{1}$	0.24
Fpz	$4.9\times 10^{-03}$	$-1.9\times 10^{-1}$	$8.5\times 10^{-4}$	$-1.5\times 10^{-3}$	$-3.6\times 10^{-1}$	$-6.7\times 10^{-7}$	$5.6\times 10^{1}$	0.16
Fp2	$1.5\times 10^{-3}$	$-8.7\times 10^{-2}$	$1.3\times 10^{-4}$	$-1.5\times 10^{-3}$	$-4.0\times 10^{-2}$	$-1.5\times 10^{-7}$	$1.2\times 10^{1}$	0.19
AF9	$4.7\times 10^{-3}$	$-4.6\times 10^{-2}$	$-2.3\times 10^{-3}$	$-2.8\times 10^{-3}$	$9.2\times 10^{-1}$	$1.9\times 10^{-6}$	$-1.2\times 10^{2}$	0.91
AF7	$2.8\times 10^{-3}$	$-3.5\times 10^{-1}$	$-4.0\times 10^{-3}$	$-2.1\times 10^{-3}$	$1.5\times 10^{0}$	$3.5\times 10^{-6}$	$-1.9\times 10^{2}$	0.24
AF5	$7.0\times 10^{-4}$	$-1.1\times 10^{-1}$	$8.3\times 10^{-5}$	$-9.8\times 10^{-4}$	$-2.0\times 10^{-2}$	$-9.4\times 10^{-8}$	$7.4\times 10^{0}$	0.17
AF3	$1.0\times 10^{-3}$	$-9.8\times 10^{-2}$	$1.9\times 10^{-4}$	$-1.1\times 10^{-3}$	$-6.0\times 10^{-2}$	$-1.8\times 10^{-7}$	$1.3\times 10^{1}$	0.18
AF1	$1.3\times 10^{-3}$	$-1.1\times 10^{-1}$	$-2.3\times 10^{-5}$	$-1.2\times 10^{-3}$	$1.4\times 10^{-2}$	$1.2\times 10^{-8}$	$3.8\times 10^{0}$	0.17
AFz	$1.5\times 10^{-3}$	$-1.2\times 10^{-1}$	$-6.5\times 10^{-4}$	$-1.4\times 10^{-3}$	$2.6\times 10^{-1}$	$5.5\times 10^{-7}$	$-2.7\times 10^{1}$	0.19
AF2	$1.1\times 10^{-3}$	$-1.2\times 10^{-1}$	$-3.9\times 10^{-4}$	$-1.3\times 10^{-3}$	$1.6\times 10^{-1}$	$3.2\times 10^{-7}$	$-1.5\times 10^{1}$	0.20
AF4	$9.8\times 10^{-4}$	$-9.4\times 10^{-2}$	$-6.1\times 10^{-5}$	$-1.2\times 10^{-3}$	$3.4\times 10^{-2}$	$3.6\times 10^{-8}$	$7.9\times 10^{-1}$	0.21
AF6	$2.0\times 10^{-4}$	$-7.9\times 10^{-2}$	$-2.7\times 10^{-4}$	$-1.1\times 10^{-3}$	$1.1\times 10^{-1}$	$2.3\times 10^{-7}$	$-8.7\times 10^{0}$	0.19
AF8	$4.4\times 10^{-4}$	$-2.7\times 10^{-2}$	$-1.7\times 10^{-3}$	$-1.8\times 10^{-3}$	$6.8\times 10^{-1}$	$1.4\times 10^{-6}$	$-8.3\times 10^{1}$	0.24
AF10	$1.9\times 10^{-3}$	$-8.1\times 10^{-2}$	$-3.8\times 10^{-3}$	$-2.9\times 10^{-3}$	$1.5\times 10^{0}$	$3.3\times 10^{-6}$	$-1.9\times 10^{2}$	0.66
F9	$3.5\times 10^{-3}$	$-3.3\times 10^{-2}$	$-2.8\times 10^{-3}$	$-2.7\times 10^{-3}$	$1.1\times 10^{0}$	$2.3\times 10^{-6}$	$-1.3\times 10^{2}$	0.91
F7	$-9.0\times 10^{-3}$	$-6.9\times 10^{-2}$	$5.9\times 10^{-4}$	$-1.8\times 10^{-3}$	$-2.1\times 10^{-1}$	$-5.2\times 10^{-7}$	$3.3\times 10^{1}$	0.21
F5	$-9.0\times 10^{-4}$	$-6.4\times 10^{-2}$	$-1.2\times 10^{-4}$	$-9.0\times 10^{-4}$	$5.0\times 10^{-2}$	$8.5\times 10^{-8}$	$-3.4\times 10^{-1}$	0.09
F3	$5.0\times 10^{-4}$	$-1.0\times 10^{-1}$	$-7.6\times 10^{-4}$	$-1.1\times 10^{-3}$	$2.9\times 10^{-1}$	$6.5\times 10^{-7}$	$-3.0\times 10^{1}$	0.13
F1	$6.9\times 10^{-4}$	$-1.1\times 10^{-1}$	$-6.7\times 10^{-4}$	$-1.1\times 10^{-3}$	$2.5\times 10^{-1}$	$6.0\times 10^{-7}$	$-2.3\times 10^{1}$	0.13
Fz	$1.5\times 10^{-3}$	$-1.2\times 10^{-1}$	$-2.0\times 10^{-3}$	$-1.2\times 10^{-3}$	$7.9\times 10^{-1}$	$1.7\times 10^{-6}$	$-9.4\times 10^{1}$	0.14
F2	$1.4\times 10^{-3}$	$-9.7\times 10^{-2}$	$-2.0\times 10^{-3}$	$-1.2\times 10^{-3}$	$8.2\times 10^{-1}$	$1.7\times 10^{-6}$	$-1.0\times 10^{2}$	0.16
F4	$4.7\times 10^{-4}$	$-7.8\times 10^{-2}$	$-6.3\times 10^{-4}$	$-1.1\times 10^{-3}$	$2.6\times 10^{-1}$	$5.2\times 10^{-7}$	$-2.8\times 10^{1}$	0.14
F6	$-8.8\times 10^{-4}$	$-8.9\times 10^{-2}$	$-6.4\times 10^{-4}$	$-9.7\times 10^{-4}$	$2.6\times 10^{-1}$	$5.3\times 10^{-7}$	$-2.7\times 10^{1}$	0.12
F8	$-5.3\times 10^{-4}$	$-1.3\times 10^{-1}$	$1.2\times 10^{-3}$	$-1.8\times 10^{-3}$	$-4.8\times 10^{-1}$	$-1.1\times 10^{-6}$	$6.9\times 10^{1}$	0.20
F10	$3.3\times 10^{-3}$	$-3.9\times 10^{-2}$	$-1.5\times 10^{-3}$	$-2.6\times 10^{-3}$	$5.9\times 10^{-1}$	$1.2\times 10^{-6}$	$-7.0\times 10^{1}$	0.82
FT9	$2.2\times 10^{-3}$	$6.4\times 10^{-2}$	$-2.7\times 10^{-3}$	$-2.2\times 10^{-3}$	$1.1\times 10^{0}$	$2.2\times 10^{-6}$	$-1.4\times 10^{2}$	0.76
FT7	$9.6\times 10^{-4}$	$5.0\times 10^{-3}$	$-1.7\times 10^{-4}$	$-2.1\times 10^{-3}$	$8.6\times 10^{-2}$	$1.1\times 10^{-7}$	$-6.8\times 10^{0}$	0.26
FC5	$-8.9\times 10^{-4}$	$-5.8\times 10^{-2}$	$5.2\times 10^{-4}$	$-1.0\times 10^{-3}$	$-2.1\times 10^{-1}$	$-4.3\times 10^{-7}$	$3.5\times 10^{1}$	0.09
FC3	$-2.6\times 10^{-4}$	$-1.1\times 10^{-1}$	$-4.0\times 10^{-4}$	$-1.1\times 10^{-3}$	$1.5\times 10^{-1}$	$3.4\times 10^{-7}$	$-1.3\times 10^{1}$	0.12
FC1	$2.0\times 10^{-3}$	$-1.2\times 10^{-1}$	$-1.3\times 10^{-3}$	$-1.3\times 10^{-3}$	$5.1\times 10^{-1}$	$1.1\times 10^{-6}$	$-5.8\times 10^{1}$	0.17
FCz	$-6.6\times 10^{-5}$	$-1.5\times 10^{-1}$	$-2.1\times 10^{-3}$	$-1.4\times 10^{-3}$	$8.4\times 10^{-1}$	$1.8\times 10^{-6}$	$-1.0\times 10^{2}$	0.21
FC2	$2.2\times 10^{-4}$	$-1.2\times 10^{-1}$	$-4.2\times 10^{-3}$	$-1.4\times 10^{-3}$	$1.6\times 10^{0}$	$3.5\times 10^{-6}$	$-2.0\times 10^{2}$	0.25
FC4	$-3.6\times 10^{-4}$	$-1.1\times 10^{-1}$	$-2.4\times 10^{-3}$	$-1.1\times 10^{-3}$	$9.5\times 10^{-1}$	$2.0\times 10^{-6}$	$-1.2\times 10^{2}$	0.18
FC6	$-1.7\times 10^{-3}$	$-9.1\times 10^{-2}$	$-1.0\times 10^{-3}$	$-9.7\times 10^{-4}$	$4.3\times 10^{-1}$	$8.3\times 10^{-7}$	$-5.2\times 10^{1}$	0.15
FT8	$2.9\times 10^{-3}$	$2.1\times 10^{-1}$	$-4.0\times 10^{-3}$	$-1.6\times 10^{-3}$	$1.6\times 10^{0}$	$3.3\times 10^{-6}$	$-2.1\times 10^{2}$	0.16
FT10	$1.8\times 10^{-3}$	$9.6\times 10^{-4}$	$-2.3\times 10^{-3}$	$-2.3\times 10^{-3}$	$9.0\times 10^{-1}$	$2.0\times 10^{-6}$	$-1.1\times 10^{2}$	0.96
T9	$1.3\times 10^{-3}$	$-2.0\times 10^{-2}$	$-3.1\times 10^{-3}$	$-2.0\times 10^{-3}$	$1.2\times 10^{0}$	$2.6\times 10^{-6}$	$-1.5\times 10^{2}$	0.97
T7	$8.9\times 10^{-4}$	$9.6\times 10^{-3}$	$-5.4\times 10^{-4}$	$-2.1\times 10^{-3}$	$2.6\times 10^{-1}$	$3.8\times 10^{-7}$	$-3.3\times 10^{1}$	0.25
C5	$-1.0\times 10^{-3}$	$-6.1\times 10^{-2}$	$5.4\times 10^{-5}$	$-1.2\times 10^{-3}$	$-1.2\times 10^{-2}$	$-6.3\times 10^{-8}$	$6.9\times 10^{0}$	0.13
C3	$-7.2\times 10^{-4}$	$-4.6\times 10^{-2}$	$-4.8\times 10^{-4}$	$-1.4\times 10^{-3}$	$2.0\times 10^{-1}$	$3.8\times 10^{-7}$	$-2.2\times 10^{1}$	0.20
C1	$1.4\times 10^{-3}$	$-3.5\times 10^{-2}$	$-1.3\times 10^{-3}$	$-1.5\times 10^{-3}$	$5.1\times 10^{-1}$	$1.0\times 10^{-6}$	$-6.1\times 10^{1}$	0.24
Cz	$2.6\times 10^{-3}$	$-1.9\times 10^{-1}$	$-2.8\times 10^{-3}$	$-1.6\times 10^{-3}$	$1.1\times 10^{0}$	$2.4\times 10^{-6}$	$-1.3\times 10^{2}$	0.18
C2	$1.2\times 10^{-3}$	$-1.6\times 10^{-1}$	$-3.1\times 10^{-3}$	$-1.5\times 10^{-3}$	$1.2\times 10^{0}$	$2.7\times 10^{-6}$	$-1.5\times 10^{2}$	0.16
C4	$-1.4\times 10^{-3}$	$-7.8\times 10^{-2}$	$-1.3\times 10^{-3}$	$-1.3\times 10^{-3}$	$5.2\times 10^{-1}$	$1.1\times 10^{-6}$	$-6.2\times 10^{1}$	0.18
C6	$-1.7\times 10^{-3}$	$-6.6\times 10^{-2}$	$-6.9\times 10^{-4}$	$-1.1\times 10^{-3}$	$2.9\times 10^{-1}$	$5.5\times 10^{-7}$	$-3.3\times 10^{1}$	0.15
T8	$-4.1\times 10^{-3}$	$-1.6\times 10^{-1}$	$-2.0\times 10^{-3}$	$-1.7\times 10^{-3}$	$7.9\times 10^{-1}$	$1.7\times 10^{-6}$	$-9.6\times 10^{1}$	0.18
T10	$2.5\times 10^{-3}$	$-2.7\times 10^{-2}$	$-4.0\times 10^{-3}$	$-1.8\times 10^{-3}$	$1.6\times 10^{0}$	$3.4\times 10^{-6}$	$-2.0\times 10^{2}$	0.21
TP9	$-7.0\times 10^{-5}$	$-5.2\times 10^{-2}$	$-1.8\times 10^{-3}$	$-1.7\times 10^{-3}$	$7.5\times 10^{-1}$	$1.4\times 10^{-6}$	$-9.5\times 10^{1}$	0.96
TP7	$-1.3\times 10^{-3}$	$-3.0\times 10^{-2}$	$5.4\times 10^{-6}$	$-2.3\times 10^{-3}$	$2.9\times 10^{-2}$	$-7.3\times 10^{-8}$	$-4.1\times 10^{-1}$	0.25
CP5	$-8.9\times 10^{-4}$	$-8.3\times 10^{-2}$	$8.1\times 10^{-4}$	$-1.5\times 10^{-3}$	$-2.9\times 10^{-1}$	$-7.4\times 10^{-7}$	$4.1\times 10^{1}$	0.23
CP3	$-2.6\times 10^{-4}$	$-4.0\times 10^{-2}$	$-2.7\times 10^{-5}$	$-1.6\times 10^{-3}$	$4.3\times 10^{-2}$	$-4.5\times 10^{-8}$	$-2.9\times 10^{0}$	0.28
CP1	$4.6\times 10^{-4}$	$-1.2\times 10^{-3}$	$-4.6\times 10^{-4}$	$-1.7\times 10^{-3}$	$2.1\times 10^{-1}$	$3.3\times 10^{-7}$	$-2.4\times 10^{1}$	0.30
CPz	$6.3\times 10^{-4}$	$-1.4\times 10^{-2}$	$-8.1\times 10^{-4}$	$-1.8\times 10^{-3}$	$3.4\times 10^{-1}$	$6.4\times 10^{-7}$	$-4.0\times 10^{1}$	0.32
CP2	$1.8\times 10^{-3}$	$-7.9\times 10^{-2}$	$-1.4\times 10^{-3}$	$-1.8\times 10^{-3}$	$5.5\times 10^{-1}$	$1.2\times 10^{-6}$	$-6.5\times 10^{1}$	0.31
CP4	$-7.7\times 10^{-4}$	$-5.4\times 10^{-2}$	$1.5\times 10^{-4}$	$-1.6\times 10^{-3}$	$-4.1\times 10^{-2}$	$-1.6\times 10^{-7}$	$9.9\times 10^{0}$	0.28
CP6	$-1.5\times 10^{-3}$	$-5.7\times 10^{-2}$	$5.6\times 10^{-4}$	$-1.4\times 10^{-3}$	$-2.0\times 10^{-1}$	$-5.0\times 10^{-7}$	$3.0\times 10^{1}$	0.17
TP8	$3.4\times 10^{-3}$	$-1.3\times 10^{-2}$	$3.8\times 10^{-4}$	$-1.9\times 10^{-3}$	$-1.2\times 10^{-1}$	$-3.6\times 10^{-7}$	$2.0\times 10^{1}$	0.26
TP10	$-9.5\times 10^{-4}$	$-7.8\times 10^{-2}$	$-3.5\times 10^{-3}$	$-1.5\times 10^{-3}$	$1.4\times 10^{0}$	$2.9\times 10^{-6}$	$-1.8\times 10^{2}$	0.54
P9	$8.0\times 10^{-4}$	$2.6\times 10^{-2}$	$-2.0\times 10^{-3}$	$-1.8\times 10^{-3}$	$8.1\times 10^{-1}$	$1.6\times 10^{-6}$	$-1.0\times 10^{2}$	0.74
P7	$2.3\times 10^{-4}$	$2.4\times 10^{-2}$	$-1.7\times 10^{-3}$	$-2.1\times 10^{-3}$	$6.4\times 10^{-1}$	$1.5\times 10^{-6}$	$-7.4\times 10^{1}$	0.33
P5	$1.3\times 10^{-3}$	$-7.4\times 10^{-2}$	$9.5\times 10^{-4}$	$-1.7\times 10^{-3}$	$-3.5\times 10^{-1}$	$-8.5\times 10^{-7}$	$5.0\times 10^{1}$	0.39
P3	$1.6\times 10^{-4}$	$-7.0\times 10^{-2}$	$-6.1\times 10^{-4}$	$-1.7\times 10^{-3}$	$2.7\times 10^{-1}$	$4.7\times 10^{-7}$	$-3.1\times 10^{1}$	0.31
P1	$9.0\times 10^{-4}$	$-3.5\times 10^{-2}$	$-1.2\times 10^{-3}$	$-1.8\times 10^{-3}$	$5.1\times 10^{-1}$	$1.0\times 10^{-6}$	$-6.2\times 10^{1}$	0.35
Pz	$5.5\times 10^{-4}$	$-2.0\times 10^{-2}$	$-7.6\times 10^{-4}$	$-1.9\times 10^{-3}$	$3.3\times 10^{-1}$	$5.8\times 10^{-7}$	$-4.0\times 10^{1}$	0.33
P2	$1.0\times 10^{-3}$	$-3.7\times 10^{-2}$	$-1.1\times 10^{-3}$	$-1.9\times 10^{-3}$	$4.6\times 10^{-1}$	$8.9\times 10^{-7}$	$-5.6\times 10^{1}$	0.33
P4	$6.6\times 10^{-4}$	$-5.9\times 10^{-2}$	$-7.5\times 10^{-4}$	$-1.7\times 10^{-3}$	$3.1\times 10^{-1}$	$6.1\times 10^{-7}$	$-3.6\times 10^{1}$	0.29
P6	$5.2\times 10^{-4}$	$-6.9\times 10^{-2}$	$9.7\times 10^{-4}$	$-1.6\times 10^{-3}$	$-3.7\times 10^{-1}$	$-8.3\times 10^{-7}$	$5.4\times 10^{1}$	0.30
P8	$1.2\times 10^{-3}$	$6.5\times 10^{-3}$	$-1.7\times 10^{-4}$	$-2.1\times 10^{-3}$	$6.9\times 10^{-2}$	$1.6\times 10^{-7}$	$-2.9\times 10^{0}$	0.30
P10	$-1.0\times 10^{-3}$	$-1.1\times 10^{-1}$	$-3.8\times 10^{-4}$	$-1.7\times 10^{-3}$	$1.9\times 10^{-1}$	$2.4\times 10^{-7}$	$-2.3\times 10^{1}$	0.86
PO9	$6.1\times 10^{-4}$	$-7.0\times 10^{-2}$	$-1.8\times 10^{-3}$	$-1.7\times 10^{-3}$	$7.1\times 10^{-1}$	$1.5\times 10^{-6}$	$-8.8\times 10^{1}$	0.58
PO7	$-4.5\times 10^{-3}$	$1.2\times 10^{-1}$	$-2.1\times 10^{-3}$	$-2.3\times 10^{-3}$	$8.3\times 10^{-1}$	$1.8\times 10^{-6}$	$-9.9\times 10^{1}$	0.31
PO5	$-1.1\times 10^{-3}$	$-8.5\times 10^{-2}$	$1.0\times 10^{-4}$	$-1.8\times 10^{-3}$	$-2.1\times 10^{-2}$	$-1.2\times 10^{-7}$	$6.7\times 10^{0}$	0.37
PO3	$6.7\times 10^{-4}$	$-6.8\times 10^{-2}$	$-1.1\times 10^{-3}$	$-1.8\times 10^{-3}$	$4.6\times 10^{-1}$	$9.4\times 10^{-7}$	$-5.5\times 10^{1}$	0.34
PO1	$9.6\times 10^{-4}$	$-4.9\times 10^{-2}$	$-1.6\times 10^{-3}$	$-1.8\times 10^{-3}$	$6.4\times 10^{-1}$	$1.3\times 10^{-6}$	$-7.7\times 10^{1}$	0.33
POz	$8.4\times 10^{-4}$	$-3.0\times 10^{-2}$	$-1.1\times 10^{-3}$	$-1.8\times 10^{-3}$	$4.7\times 10^{-1}$	$9.4\times 10^{-7}$	$-5.6\times 10^{1}$	0.35
PO2	$7.8\times 10^{-4}$	$-6.3\times 10^{-2}$	$-1.2\times 10^{-3}$	$-1.8\times 10^{-3}$	$4.9\times 10^{-1}$	$9.5\times 10^{-7}$	$-6.0\times 10^{1}$	0.36
PO4	$3.2\times 10^{-4}$	$-8.4\times 10^{-2}$	$-1.4\times 10^{-3}$	$-1.8\times 10^{-3}$	$5.6\times 10^{-1}$	$1.1\times 10^{-6}$	$-6.8\times 10^{1}$	0.34
PO6	$4.6\times 10^{-4}$	$-7.6\times 10^{-2}$	$1.0\times 10^{-3}$	$-1.9\times 10^{-3}$	$-3.9\times 10^{-1}$	$-8.7\times 10^{-7}$	$5.6\times 10^{1}$	0.53
PO8	$-1.5\times 10^{-4}$	$-1.2\times 10^{-1}$	$-2.4\times 10^{-4}$	$-2.3\times 10^{-3}$	$7.4\times 10^{-2}$	$2.5\times 10^{-7}$	$4.7\times 10^{-2}$	0.53
PO10	$1.2\times 10^{-3}$	$-5.1\times 10^{-2}$	$-2.1\times 10^{-3}$	$-1.9\times 10^{-3}$	$8.3\times 10^{-1}$	$1.8\times 10^{-6}$	$-1.0\times 10^{2}$	0.98
O1	$-1.9\times 10^{-3}$	$4.2\times 10^{-3}$	$-5.0\times 10^{-4}$	$-2.4\times 10^{-3}$	$1.7\times 10^{-1}$	$4.6\times 10^{-7}$	$-1.2\times 10^{1}$	0.39
Oz	$1.2\times 10^{-3}$	$-4.7\times 10^{-2}$	$1.7\times 10^{-4}$	$-1.9\times 10^{-3}$	$-5.1\times 10^{-2}$	$-1.7\times 10^{-7}$	$1.1\times 10^{1}$	0.49
O2	$-1.5\times 10^{-3}$	$7.4\times 10^{-2}$	$6.7\times 10^{-4}$	$-2.4\times 10^{-3}$	$-2.7\times 10^{-1}$	$-5.7\times 10^{-7}$	$4.0\times 10^{1}$	0.36
I1	$1.2\times 10^{-3}$	$-7.8\times 10^{-2}$	$-3.0\times 10^{-3}$	$-2.0\times 10^{-3}$	$1.2\times 10^{0}$	$2.5\times 10^{-6}$	$-1.5\times 10^{2}$	0.89
Iz	$6.9\times 10^{-4}$	$-5.7\times 10^{-2}$	$-3.1\times 10^{-3}$	$-2.0\times 10^{-3}$	$1.2\times 10^{0}$	$2.5\times 10^{-6}$	$-1.6\times 10^{2}$	0.39
I2	$1.3\times 10^{-3}$	$-5.4\times 10^{-2}$	$-2.8\times 10^{-3}$	$-2.0\times 10^{-3}$	$1.1\times 10^{0}$	$2.3\times 10^{-6}$	$-1.4\times 10^{2}$	0.61
AFp9h	$5.5\times 10^{-3}$	$-1.0\times 10^{-1}$	$-6.7\times 10^{-3}$	$-2.7\times 10^{-3}$	$2.6\times 10^{0}$	$5.7\times 10^{-6}$	$-3.3\times 10^{2}$	0.44
AFp7h	$-1.7\times 10^{-4}$	$-9.4\times 10^{-2}$	$-7.2\times 10^{-4}$	$-1.4\times 10^{-3}$	$3.1\times 10^{-1}$	$5.7\times 10^{-7}$	$-3.6\times 10^{1}$	0.19
AFp5h	$3.1\times 10^{-4}$	$-8.0\times 10^{-2}$	$2.6\times 10^{-4}$	$-1.1\times 10^{-3}$	$-8.1\times 10^{-2}$	$-2.5\times 10^{-7}$	$1.4\times 10^{1}$	0.18
AFp3h	$1.2\times 10^{-3}$	$-9.4\times 10^{-2}$	$3.7\times 10^{-4}$	$-1.2\times 10^{-3}$	$-1.4\times 10^{-1}$	$-3.3\times 10^{-7}$	$2.3\times 10^{1}$	0.18
AFp1h	$2.1\times 10^{-3}$	$-1.2\times 10^{-1}$	$1.6\times 10^{-5}$	$-1.4\times 10^{-3}$	$-5.6\times 10^{-3}$	$-1.2\times 10^{-8}$	$7.5\times 10^{0}$	0.18
AFp2h	$1.6\times 10^{-3}$	$-1.1\times 10^{-1}$	$8.9\times 10^{-5}$	$-1.5\times 10^{-3}$	$-3.6\times 10^{-2}$	$-7.1\times 10^{-8}$	$1.2\times 10^{1}$	0.18
AFp4h	$6.9\times 10^{-4}$	$-9.1\times 10^{-2}$	$3.6\times 10^{-4}$	$-1.4\times 10^{-3}$	$-1.3\times 10^{-1}$	$-3.2\times 10^{-7}$	$2.2\times 10^{1}$	0.19
AFp6h	$3.7\times 10^{-4}$	$-7.2\times 10^{-2}$	$4.7\times 10^{-4}$	$-1.4\times 10^{-3}$	$-1.7\times 10^{-1}$	$-4.2\times 10^{-7}$	$2.7\times 10^{1}$	0.21
AFp8h	$4.4\times 10^{-3}$	$-5.7\times 10^{-2}$	$6.6\times 10^{-4}$	$-1.6\times 10^{-3}$	$-2.7\times 10^{-1}$	$-5.3\times 10^{-7}$	$4.4\times 10^{1}$	0.20
AFp10h	$1.4\times 10^{-3}$	$3.5\times 10^{-2}$	$-8.3\times 10^{-4}$	$-2.8\times 10^{-3}$	$3.3\times 10^{-1}$	$6.6\times 10^{-7}$	$-3.1\times 10^{1}$	0.39
AFF9h	$3.3\times 10^{-3}$	$-2.3\times 10^{-2}$	$-5.9\times 10^{-3}$	$-3.2\times 10^{-3}$	$2.3\times 10^{0}$	$4.9\times 10^{-6}$	$-3.0\times 10^{2}$	0.26
AFF7h	$3.6\times 10^{-5}$	$-1.2\times 10^{-1}$	$-8.9\times 10^{-4}$	$-9.2\times 10^{-4}$	$3.7\times 10^{-1}$	$7.1\times 10^{-7}$	$-4.5\times 10^{1}$	0.21
AFF5h	$7.0\times 10^{-4}$	$-1.0\times 10^{-1}$	$-9.9\times 10^{-5}$	$-9.9\times 10^{-4}$	$4.9\times 10^{-2}$	$6.3\times 10^{-8}$	$-1.0\times 10^{0}$	0.15
AFF3h	$6.1\times 10^{-4}$	$-1.0\times 10^{-1}$	$-1.7\times 10^{-4}$	$-1.1\times 10^{-3}$	$7.1\times 10^{-2}$	$1.4\times 10^{-7}$	$-2.7\times 10^{0}$	0.15
AFF1h	$1.2\times 10^{-3}$	$-1.3\times 10^{-1}$	$-4.0\times 10^{-4}$	$-1.2\times 10^{-3}$	$1.6\times 10^{-1}$	$3.4\times 10^{-7}$	$-1.4\times 10^{1}$	0.17
AFF2h	$1.6\times 10^{-3}$	$-1.3\times 10^{-1}$	$-1.3\times 10^{-3}$	$-1.3\times 10^{-3}$	$5.2\times 10^{-1}$	$1.1\times 10^{-6}$	$-6.1\times 10^{1}$	0.19
AFF4h	$1.1\times 10^{-3}$	$-1.1\times 10^{-1}$	$-6.1\times 10^{-4}$	$-1.2\times 10^{-3}$	$2.5\times 10^{-1}$	$4.9\times 10^{-7}$	$-2.8\times 10^{1}$	0.19
AFF6h	$-6.5\times 10^{-4}$	$-1.2\times 10^{-1}$	$-1.1\times 10^{-3}$	$-1.0\times 10^{-3}$	$4.4\times 10^{-1}$	$9.6\times 10^{-7}$	$-5.1\times 10^{1}$	0.15
AFF8h	$-1.4\times 10^{-3}$	$-1.4\times 10^{-1}$	$-5.5\times 10^{-4}$	$-9.8\times 10^{-4}$	$2.3\times 10^{-1}$	$4.5\times 10^{-7}$	$-2.4\times 10^{1}$	0.15
AFF10h	$4.7\times 10^{-3}$	$-1.8\times 10^{-1}$	$-4.7\times 10^{-3}$	$-3.0\times 10^{-3}$	$1.8\times 10^{0}$	$3.9\times 10^{-6}$	$-2.3\times 10^{2}$	0.30
FFT9h	$1.0\times 10^{-3}$	$1.2\times 10^{-2}$	$-1.8\times 10^{-3}$	$-2.6\times 10^{-3}$	$7.0\times 10^{-1}$	$1.5\times 10^{-6}$	$-8.5\times 10^{1}$	0.28
FFT7h	$-2.3\times 10^{-3}$	$-2.5\times 10^{-2}$	$7.0\times 10^{-4}$	$-1.1\times 10^{-3}$	$-2.6\times 10^{-1}$	$-6.1\times 10^{-7}$	$4.0\times 10^{1}$	0.19
FFC5h	$-3.3\times 10^{-4}$	$-8.3\times 10^{-2}$	$-3.6\times 10^{-4}$	$-9.8\times 10^{-4}$	$1.3\times 10^{-1}$	$3.2\times 10^{-7}$	$-9.5\times 10^{0}$	0.10
FFC3h	$8.8\times 10^{-4}$	$-1.1\times 10^{-1}$	$-7.7\times 10^{-4}$	$-1.2\times 10^{-3}$	$2.8\times 10^{-1}$	$6.8\times 10^{-7}$	$-2.8\times 10^{1}$	0.14
FFC1h	$1.2\times 10^{-3}$	$-1.1\times 10^{-1}$	$-1.0\times 10^{-3}$	$-1.2\times 10^{-3}$	$3.8\times 10^{-1}$	$9.3\times 10^{-7}$	$-4.0\times 10^{1}$	0.13
FFC2h	$9.4\times 10^{-4}$	$-1.3\times 10^{-1}$	$-3.1\times 10^{-3}$	$-1.3\times 10^{-3}$	$1.2\times 10^{0}$	$2.7\times 10^{-6}$	$-1.5\times 10^{2}$	0.24
FFC4h	$7.8\times 10^{-4}$	$-8.5\times 10^{-2}$	$-2.2\times 10^{-3}$	$-1.1\times 10^{-3}$	$8.6\times 10^{-1}$	$1.8\times 10^{-6}$	$-1.1\times 10^{2}$	0.24
FFC6h	$-2.6\times 10^{-4}$	$-7.9\times 10^{-2}$	$-1.2\times 10^{-3}$	$-1.0\times 10^{-3}$	$4.7\times 10^{-1}$	$9.6\times 10^{-7}$	$-5.7\times 10^{1}$	0.14
FFT8h	$-2.6\times 10^{-3}$	$3.3\times 10^{-3}$	$2.6\times 10^{-4}$	$-1.1\times 10^{-3}$	$-6.8\times 10^{-2}$	$-2.7\times 10^{-7}$	$1.1\times 10^{1}$	0.15
FFT10h	$3.1\times 10^{-3}$	$9.7\times 10^{-2}$	$-4.0\times 10^{-3}$	$-2.5\times 10^{-3}$	$1.6\times 10^{0}$	$3.4\times 10^{-6}$	$-2.0\times 10^{2}$	0.24
FTT9h	$4.4\times 10^{-3}$	$3.6\times 10^{-2}$	$-3.9\times 10^{-3}$	$-2.6\times 10^{-3}$	$1.5\times 10^{0}$	$3.3\times 10^{-6}$	$-1.9\times 10^{2}$	0.29
FTT7h	$-1.6\times 10^{-4}$	$-4.1\times 10^{-2}$	$3.2\times 10^{-4}$	$-1.5\times 10^{-3}$	$-1.3\times 10^{-1}$	$-2.6\times 10^{-7}$	$2.4\times 10^{1}$	0.16
FCC5h	$-1.0\times 10^{-3}$	$-6.3\times 10^{-2}$	$1.9\times 10^{-4}$	$-1.1\times 10^{-3}$	$-7.7\times 10^{-2}$	$-1.6\times 10^{-7}$	$1.7\times 10^{1}$	0.10
FCC3h	$2.7\times 10^{-5}$	$-8.1\times 10^{-2}$	$-6.6\times 10^{-4}$	$-1.4\times 10^{-3}$	$2.7\times 10^{-1}$	$5.4\times 10^{-7}$	$-3.0\times 10^{1}$	0.21
FCC1h	$2.1\times 10^{-3}$	$-1.4\times 10^{-1}$	$-2.2\times 10^{-3}$	$-1.4\times 10^{-3}$	$8.8\times 10^{-1}$	$1.9\times 10^{-6}$	$-1.1\times 10^{2}$	0.21
FCC2h	$5.3\times 10^{-4}$	$-2.4\times 10^{-1}$	$-4.4\times 10^{-3}$	$-1.5\times 10^{-3}$	$1.7\times 10^{0}$	$3.8\times 10^{-6}$	$-2.1\times 10^{2}$	0.18
FCC4h	$-1.5\times 10^{-3}$	$-1.1\times 10^{-1}$	$-3.3\times 10^{-3}$	$-1.3\times 10^{-3}$	$1.3\times 10^{0}$	$2.8\times 10^{-6}$	$-1.6\times 10^{2}$	0.17
FCC6h	$-1.2\times 10^{-3}$	$-8.3\times 10^{-2}$	$-1.3\times 10^{-3}$	$-1.0\times 10^{-3}$	$5.3\times 10^{-1}$	$1.1\times 10^{-6}$	$-6.4\times 10^{1}$	0.14
FTT8h	$-2.5\times 10^{-3}$	$-8.4\times 10^{-2}$	$-2.0\times 10^{-3}$	$-1.1\times 10^{-3}$	$8.1\times 10^{-1}$	$1.6\times 10^{-6}$	$-1.0\times 10^{2}$	0.12
FTT10h	$-1.8\times 10^{-3}$	$-4.1\times 10^{-2}$	$-2.4\times 10^{-3}$	$-1.9\times 10^{-3}$	$9.9\times 10^{-1}$	$2.0\times 10^{-6}$	$-1.3\times 10^{2}$	0.18
TTP9h	$2.0\times 10^{-3}$	$-6.7\times 10^{-3}$	$-1.5\times 10^{-3}$	$-2.1\times 10^{-3}$	$6.0\times 10^{-1}$	$1.3\times 10^{-6}$	$-7.0\times 10^{1}$	0.15
TTP7h	$-5.3\times 10^{-4}$	$-1.1\times 10^{-1}$	$2.1\times 10^{-3}$	$-1.5\times 10^{-3}$	$-8.1\times 10^{-1}$	$-1.9\times 10^{-6}$	$1.1\times 10^{2}$	0.26
CCP5h	$-9.6\times 10^{-4}$	$-5.1\times 10^{-2}$	$-2.3\times 10^{-4}$	$-1.3\times 10^{-3}$	$1.1\times 10^{-1}$	$1.5\times 10^{-7}$	$-1.1\times 10^{1}$	0.17
CCP3h	$2.7\times 10^{-4}$	$-1.8\times 10^{-2}$	$-4.3\times 10^{-4}$	$-1.7\times 10^{-3}$	$1.9\times 10^{-1}$	$3.1\times 10^{-7}$	$-2.1\times 10^{1}$	0.26
CCP1h	$1.3\times 10^{-3}$	$-5.0\times 10^{-4}$	$-1.1\times 10^{-3}$	$-1.6\times 10^{-3}$	$4.4\times 10^{-1}$	$9.1\times 10^{-7}$	$-5.2\times 10^{1}$	0.24
CCP2h	$3.4\times 10^{-3}$	$-1.7\times 10^{-1}$	$-1.9\times 10^{-3}$	$-1.7\times 10^{-3}$	$7.3\times 10^{-1}$	$1.7\times 10^{-6}$	$-8.5\times 10^{1}$	0.18
CCP4h	$-4.2\times 10^{-4}$	$-6.6\times 10^{-2}$	$-1.2\times 10^{-3}$	$-1.5\times 10^{-3}$	$4.9\times 10^{-1}$	$1.0\times 10^{-6}$	$-5.9\times 10^{1}$	0.26
CCP6h	$-2.1\times 10^{-3}$	$-6.1\times 10^{-2}$	$-2.6\times 10^{-4}$	$-1.3\times 10^{-3}$	$1.2\times 10^{-1}$	$1.9\times 10^{-7}$	$-1.1\times 10^{1}$	0.20
TTP8h	$8.8\times 10^{-4}$	$1.1\times 10^{-2}$	$8.0\times 10^{-4}$	$-1.4\times 10^{-3}$	$-2.8\times 10^{-1}$	$-7.4\times 10^{-7}$	$3.8\times 10^{1}$	0.19
TTP10h	$1.6\times 10^{-3}$	$-4.6\times 10^{-2}$	$-2.7\times 10^{-3}$	$-2.3\times 10^{-3}$	$1.1\times 10^{0}$	$2.3\times 10^{-6}$	$-1.4\times 10^{2}$	0.24
TPP9h	$-1.2\times 10^{-4}$	$-4.0\times 10^{-4}$	$-8.9\times 10^{-4}$	$-1.9\times 10^{-3}$	$3.7\times 10^{-1}$	$7.1\times 10^{-7}$	$-4.4\times 10^{1}$	0.20
TPP7h	$4.4\times 10^{-4}$	$-1.1\times 10^{-1}$	$6.5\times 10^{-4}$	$-1.8\times 10^{-3}$	$-2.3\times 10^{-1}$	$-5.8\times 10^{-7}$	$3.4\times 10^{1}$	0.30
CPP5h	$-3.8\times 10^{-4}$	$-6.6\times 10^{-2}$	$1.6\times 10^{-4}$	$-1.6\times 10^{-3}$	$-3.0\times 10^{-2}$	$-2.0\times 10^{-7}$	$6.5\times 10^{0}$	0.27
CPP3h	$1.8\times 10^{-4}$	$-4.4\times 10^{-2}$	$-4.8\times 10^{-4}$	$-1.7\times 10^{-3}$	$2.2\times 10^{-1}$	$3.6\times 10^{-7}$	$-2.5\times 10^{1}$	0.33
CPP1h	$7.6\times 10^{-4}$	$-4.4\times 10^{-3}$	$-6.0\times 10^{-4}$	$-1.8\times 10^{-3}$	$2.7\times 10^{-1}$	$4.5\times 10^{-7}$	$-3.2\times 10^{1}$	0.34
CPP2h	$6.3\times 10^{-4}$	$-2.4\times 10^{-2}$	$-9.8\times 10^{-4}$	$-1.9\times 10^{-3}$	$4.1\times 10^{-1}$	$7.8\times 10^{-7}$	$-5.0\times 10^{1}$	0.32
CPP4h	$1.8\times 10^{-4}$	$-4.7\times 10^{-2}$	$-7.4\times 10^{-4}$	$-1.8\times 10^{-3}$	$3.1\times 10^{-1}$	$5.9\times 10^{-7}$	$-3.6\times 10^{1}$	0.33
CPP6h	$-2.3\times 10^{-4}$	$-6.6\times 10^{-2}$	$8.5\times 10^{-4}$	$-1.6\times 10^{-3}$	$-3.2\times 10^{-1}$	$-7.4\times 10^{-7}$	$4.7\times 10^{1}$	0.23
TPP8h	$-4.6\times 10^{-4}$	$-4.1\times 10^{-2}$	$1.2\times 10^{-3}$	$-1.6\times 10^{-3}$	$-4.5\times 10^{-1}$	$-1.0\times 10^{-6}$	$6.4\times 10^{1}$	0.25
TPP10h	$-2.1\times 10^{-4}$	$1.1\times 10^{-2}$	$-1.5\times 10^{-4}$	$-1.8\times 10^{-3}$	$7.0\times 10^{-2}$	$8.7\times 10^{-8}$	$-4.1\times 10^{0}$	0.21
PPO9h	$9.3\times 10^{-4}$	$-5.7\times 10^{-2}$	$-1.5\times 10^{-3}$	$-2.2\times 10^{-3}$	$6.0\times 10^{-1}$	$1.2\times 10^{-6}$	$-7.1\times 10^{1}$	0.37
PPO7h	$-4.2\times 10^{-4}$	$-1.7\times 10^{-2}$	$1.9\times 10^{-3}$	$-2.1\times 10^{-3}$	$-7.4\times 10^{-1}$	$-1.6\times 10^{-6}$	$1.0\times 10^{2}$	0.41
PPO5h	$2.0\times 10^{-4}$	$-9.3\times 10^{-2}$	$-6.5\times 10^{-4}$	$-1.7\times 10^{-3}$	$2.8\times 10^{-1}$	$5.0\times 10^{-7}$	$-3.3\times 10^{1}$	0.32
PPO3h	$8.2\times 10^{-4}$	$-5.9\times 10^{-2}$	$-1.8\times 10^{-3}$	$-1.8\times 10^{-3}$	$7.2\times 10^{-1}$	$1.5\times 10^{-6}$	$-8.9\times 10^{1}$	0.32
PPO1h	$6.6\times 10^{-4}$	$-3.6\times 10^{-2}$	$-1.6\times 10^{-3}$	$-1.8\times 10^{-3}$	$6.5\times 10^{-1}$	$1.3\times 10^{-6}$	$-8.0\times 10^{1}$	0.33
PPO2h	$7.2\times 10^{-4}$	$-3.4\times 10^{-2}$	$-9.6\times 10^{-4}$	$-1.8\times 10^{-3}$	$4.1\times 10^{-1}$	$7.6\times 10^{-7}$	$-5.0\times 10^{1}$	0.33
PPO4h	$9.0\times 10^{-4}$	$-6.8\times 10^{-2}$	$-1.2\times 10^{-3}$	$-1.9\times 10^{-3}$	$4.7\times 10^{-1}$	$9.4\times 10^{-7}$	$-5.7\times 10^{1}$	0.32
PPO6h	$7.7\times 10^{-4}$	$-6.9\times 10^{-2}$	$-2.6\times 10^{-4}$	$-1.8\times 10^{-3}$	$1.2\times 10^{-1}$	$2.0\times 10^{-7}$	$-1.0\times 10^{1}$	0.33
PPO8h	$1.3\times 10^{-3}$	$-9.1\times 10^{-2}$	$9.1\times 10^{-4}$	$-1.7\times 10^{-3}$	$-3.6\times 10^{-1}$	$-7.7\times 10^{-7}$	$5.5\times 10^{1}$	0.37
PPO10h	$-2.6\times 10^{-3}$	$-6.2\times 10^{-2}$	$-1.0\times 10^{-3}$	$-2.3\times 10^{-3}$	$4.2\times 10^{-1}$	$8.6\times 10^{-7}$	$-4.8\times 10^{1}$	0.25
POO9h	$8.5\times 10^{-4}$	$6.7\times 10^{-3}$	$-1.5\times 10^{-3}$	$-2.1\times 10^{-3}$	$6.1\times 10^{-1}$	$1.2\times 10^{-6}$	$-7.7\times 10^{1}$	0.37
POO7h	$-4.8\times 10^{-6}$	$-5.2\times 10^{-2}$	$1.2\times 10^{-3}$	$-2.2\times 10^{-3}$	$-4.8\times 10^{-1}$	$-1.0\times 10^{-6}$	$7.0\times 10^{1}$	0.45
POO5h	$-1.0\times 10^{-3}$	$-1.1\times 10^{-2}$	$-9.4\times 10^{-4}$	$-2.0\times 10^{-3}$	$3.8\times 10^{-1}$	$7.8\times 10^{-7}$	$-4.5\times 10^{1}$	0.34
POO3h	$-2.7\times 10^{-4}$	$-9.4\times 10^{-2}$	$-1.6\times 10^{-4}$	$-1.8\times 10^{-3}$	$6.7\times 10^{-2}$	$1.4\times 10^{-7}$	$-2.1\times 10^{0}$	0.34
POO1h	$1.4\times 10^{-3}$	$-8.7\times 10^{-2}$	$-1.6\times 10^{-3}$	$-1.9\times 10^{-3}$	$6.4\times 10^{-1}$	$1.3\times 10^{-6}$	$-7.8\times 10^{1}$	0.47
POO2h	$1.6\times 10^{-3}$	$-6.2\times 10^{-2}$	$-5.8\times 10^{-4}$	$-1.8\times 10^{-3}$	$2.4\times 10^{-1}$	$4.7\times 10^{-7}$	$-2.7\times 10^{1}$	0.38
POO4h	$7.6\times 10^{-4}$	$-8.1\times 10^{-2}$	$-8.9\times 10^{-4}$	$-1.8\times 10^{-3}$	$3.7\times 10^{-1}$	$7.1\times 10^{-7}$	$-4.4\times 10^{1}$	0.39
POO6h	$-3.5\times 10^{-4}$	$-9.6\times 10^{-2}$	$3.4\times 10^{-4}$	$-1.9\times 10^{-3}$	$-1.2\times 10^{-1}$	$-3.1\times 10^{-7}$	$2.0\times 10^{1}$	0.42
POO8h	$-1.4\times 10^{-3}$	$-3.6\times 10^{-2}$	$-8.9\times 10^{-4}$	$-2.0\times 10^{-3}$	$3.5\times 10^{-1}$	$7.5\times 10^{-7}$	$-3.9\times 10^{1}$	0.31
POO10h	$2.5\times 10^{-3}$	$-7.8\times 10^{-2}$	$-2.7\times 10^{-3}$	$-2.2\times 10^{-3}$	$1.1\times 10^{0}$	$2.3\times 10^{-6}$	$-1.3\times 10^{2}$	0.38
OI1h	$7.2\times 10^{-4}$	$-1.1\times 10^{-1}$	$-3.8\times 10^{-3}$	$-2.3\times 10^{-3}$	$1.5\times 10^{0}$	$3.2\times 10^{-6}$	$-1.9\times 10^{2}$	0.40
OI2h	$7.6\times 10^{-4}$	$-1.2\times 10^{-1}$	$-4.0\times 10^{-3}$	$-2.1\times 10^{-3}$	$1.6\times 10^{0}$	$3.4\times 10^{-6}$	$-1.9\times 10^{2}$	0.36
Fp1h	$3.1\times 10^{-3}$	$-1.6\times 10^{-1}$	$8.1\times 10^{-4}$	$-1.4\times 10^{-3}$	$-3.3\times 10^{-1}$	$-6.6\times 10^{-7}$	$5.2\times 10^{1}$	0.14
Fp2h	$3.1\times 10^{-3}$	$-1.7\times 10^{-1}$	$3.1\times 10^{-4}$	$-1.4\times 10^{-3}$	$-1.3\times 10^{-1}$	$-2.6\times 10^{-7}$	$2.3\times 10^{1}$	0.16
AF9h	$2.2\times 10^{-3}$	$-6.2\times 10^{-2}$	$-3.4\times 10^{-3}$	$-3.2\times 10^{-3}$	$1.4\times 10^{0}$	$2.9\times 10^{-6}$	$-1.7\times 10^{2}$	0.37
AF7h	$1.6\times 10^{-3}$	$-1.8\times 10^{-1}$	$-2.2\times 10^{-4}$	$-9.8\times 10^{-4}$	$1.1\times 10^{-1}$	$1.6\times 10^{-7}$	$-9.5\times 10^{0}$	0.21
AF5h	$1.0\times 10^{-3}$	$-1.0\times 10^{-1}$	$1.1\times 10^{-4}$	$-1.1\times 10^{-3}$	$-3.2\times 10^{-2}$	$-1.2\times 10^{-7}$	$9.2\times 10^{0}$	0.17
AF3h	$1.1\times 10^{-3}$	$-1.0\times 10^{-1}$	$1.5\times 10^{-4}$	$-1.1\times 10^{-3}$	$-4.8\times 10^{-2}$	$-1.5\times 10^{-7}$	$1.1\times 10^{1}$	0.18
AF1h	$1.7\times 10^{-3}$	$-1.2\times 10^{-1}$	$-3.5\times 10^{-4}$	$-1.3\times 10^{-3}$	$1.4\times 10^{-1}$	$2.9\times 10^{-7}$	$-1.1\times 10^{1}$	0.18
AF2h	$1.3\times 10^{-3}$	$-1.1\times 10^{-1}$	$-6.2\times 10^{-4}$	$-1.4\times 10^{-3}$	$2.5\times 10^{-1}$	$5.2\times 10^{-7}$	$-2.6\times 10^{1}$	0.19
AF4h	$1.1\times 10^{-3}$	$-1.1\times 10^{-1}$	$-1.5\times 10^{-4}$	$-1.3\times 10^{-3}$	$6.8\times 10^{-2}$	$1.1\times 10^{-7}$	$-3.6\times 10^{0}$	0.21
AF6h	$7.0\times 10^{-4}$	$-7.7\times 10^{-2}$	$1.6\times 10^{-5}$	$-1.1\times 10^{-3}$	$2.8\times 10^{-3}$	$-2.7\times 10^{-8}$	$4.9\times 10^{0}$	0.21
AF8h	$4.5\times 10^{-5}$	$-1.1\times 10^{-1}$	$-1.3\times 10^{-3}$	$-1.1\times 10^{-3}$	$5.0\times 10^{-1}$	$1.1\times 10^{-6}$	$-5.9\times 10^{1}$	0.17
AF10h	$2.5\times 10^{-3}$	$-4.2\times 10^{-2}$	$-2.5\times 10^{-3}$	$-3.3\times 10^{-3}$	$9.7\times 10^{-1}$	$2.1\times 10^{-6}$	$-1.2\times 10^{2}$	0.41
F9h	$1.7\times 10^{-3}$	$-7.1\times 10^{-2}$	$-3.8\times 10^{-3}$	$-2.9\times 10^{-3}$	$1.5\times 10^{0}$	$3.2\times 10^{-6}$	$-1.9\times 10^{2}$	0.32
F7h	$-4.2\times 10^{-3}$	$-2.9\times 10^{-2}$	$-2.0\times 10^{-4}$	$-1.1\times 10^{-3}$	$9.5\times 10^{-2}$	$1.4\times 10^{-7}$	$-7.7\times 10^{0}$	0.15
F5h	$3.1\times 10^{-5}$	$-9.1\times 10^{-2}$	$-5.7\times 10^{-4}$	$-9.8\times 10^{-4}$	$2.2\times 10^{-1}$	$4.8\times 10^{-7}$	$-2.2\times 10^{1}$	0.11
F3h	$5.9\times 10^{-4}$	$-1.1\times 10^{-1}$	$-6.4\times 10^{-4}$	$-1.2\times 10^{-3}$	$2.4\times 10^{-1}$	$5.7\times 10^{-7}$	$-2.3\times 10^{1}$	0.13
F1h	$1.1\times 10^{-3}$	$-1.2\times 10^{-1}$	$-1.1\times 10^{-3}$	$-1.1\times 10^{-3}$	$4.0\times 10^{-1}$	$9.4\times 10^{-7}$	$-4.2\times 10^{1}$	0.14
F2h	$1.7\times 10^{-3}$	$-1.1\times 10^{-1}$	$-2.5\times 10^{-3}$	$-1.2\times 10^{-3}$	$9.8\times 10^{-1}$	$2.1\times 10^{-6}$	$-1.2\times 10^{2}$	0.16
F4h	$1.1\times 10^{-3}$	$-8.0\times 10^{-2}$	$-1.2\times 10^{-3}$	$-1.1\times 10^{-3}$	$4.9\times 10^{-1}$	$9.8\times 10^{-7}$	$-5.9\times 10^{1}$	0.16
F6h	$-3.9\times 10^{-4}$	$-7.2\times 10^{-2}$	$-5.5\times 10^{-4}$	$-1.0\times 10^{-3}$	$2.3\times 10^{-1}$	$4.5\times 10^{-7}$	$-2.4\times 10^{1}$	0.11
F8h	$-1.6\times 10^{-3}$	$-7.4\times 10^{-2}$	$8.0\times 10^{-4}$	$-1.1\times 10^{-3}$	$-3.0\times 10^{-1}$	$-7.0\times 10^{-7}$	$4.5\times 10^{1}$	0.16
F10h	$5.4\times 10^{-3}$	$-2.0\times 10^{-2}$	$-1.2\times 10^{-3}$	$-2.9\times 10^{-3}$	$5.2\times 10^{-1}$	$9.5\times 10^{-7}$	$-6.2\times 10^{1}$	0.29
FT9h	$-1.4\times 10^{-4}$	$1.6\times 10^{-1}$	$-2.1\times 10^{-3}$	$-2.5\times 10^{-3}$	$8.7\times 10^{-1}$	$1.8\times 10^{-6}$	$-1.1\times 10^{2}$	0.19
FT7h	$-1.1\times 10^{-4}$	$-6.6\times 10^{-2}$	$7.4\times 10^{-5}$	$-1.4\times 10^{-3}$	$-2.4\times 10^{-2}$	$-6.4\times 10^{-8}$	$1.0\times 10^{1}$	0.16
FC5h	$-7.0\times 10^{-4}$	$-8.1\times 10^{-2}$	$1.1\times 10^{-4}$	$-1.0\times 10^{-3}$	$-4.7\times 10^{-2}$	$-7.6\times 10^{-8}$	$1.4\times 10^{1}$	0.09
FC3h	$4.2\times 10^{-4}$	$-1.1\times 10^{-1}$	$-6.2\times 10^{-4}$	$-1.2\times 10^{-3}$	$2.4\times 10^{-1}$	$5.3\times 10^{-7}$	$-2.4\times 10^{1}$	0.16
FC1h	$1.9\times 10^{-3}$	$-1.2\times 10^{-1}$	$-1.6\times 10^{-3}$	$-1.3\times 10^{-3}$	$6.1\times 10^{-1}$	$1.3\times 10^{-6}$	$-7.1\times 10^{1}$	0.20
FC2h	$-9.3\times 10^{-5}$	$-1.7\times 10^{-1}$	$-3.9\times 10^{-3}$	$-1.4\times 10^{-3}$	$1.5\times 10^{0}$	$3.3\times 10^{-6}$	$-1.9\times 10^{2}$	0.24
FC4h	$-1.5\times 10^{-5}$	$-1.1\times 10^{-1}$	$-3.4\times 10^{-3}$	$-1.2\times 10^{-3}$	$1.3\times 10^{0}$	$2.9\times 10^{-6}$	$-1.7\times 10^{2}$	0.24
FC6h	$-1.0\times 10^{-3}$	$-9.9\times 10^{-2}$	$-1.8\times 10^{-3}$	$-1.0\times 10^{-3}$	$7.1\times 10^{-1}$	$1.5\times 10^{-6}$	$-8.8\times 10^{1}$	0.15
FT8h	$-1.4\times 10^{-3}$	$-2.2\times 10^{-2}$	$-9.6\times 10^{-4}$	$-1.1\times 10^{-3}$	$4.1\times 10^{-1}$	$7.6\times 10^{-7}$	$-5.1\times 10^{1}$	0.16
FT10h	$7.7\times 10^{-3}$	$1.7\times 10^{-1}$	$-4.3\times 10^{-3}$	$-2.5\times 10^{-3}$	$1.7\times 10^{0}$	$3.7\times 10^{-6}$	$-2.1\times 10^{2}$	0.24
T9h	$4.2\times 10^{-3}$	$1.4\times 10^{-1}$	$-1.1\times 10^{-3}$	$-2.4\times 10^{-3}$	$4.8\times 10^{-1}$	$8.6\times 10^{-7}$	$-6.1\times 10^{1}$	0.24
T7h	$1.4\times 10^{-4}$	$-8.8\times 10^{-2}$	$4.8\times 10^{-4}$	$-1.4\times 10^{-3}$	$-1.7\times 10^{-1}$	$-4.4\times 10^{-7}$	$2.6\times 10^{1}$	0.24
C5h	$-1.4\times 10^{-3}$	$-5.6\times 10^{-2}$	$-3.7\times 10^{-4}$	$-1.2\times 10^{-3}$	$1.6\times 10^{-1}$	$2.8\times 10^{-7}$	$-1.5\times 10^{1}$	0.12
C3h	$5.0\times 10^{-4}$	$-4.5\times 10^{-2}$	$-8.2\times 10^{-4}$	$-1.6\times 10^{-3}$	$3.3\times 10^{-1}$	$6.6\times 10^{-7}$	$-3.9\times 10^{1}$	0.24
C1h	$2.3\times 10^{-3}$	$-6.4\times 10^{-2}$	$-1.5\times 10^{-3}$	$-1.5\times 10^{-3}$	$6.0\times 10^{-1}$	$1.3\times 10^{-6}$	$-7.2\times 10^{1}$	0.21
C2h	$2.9\times 10^{-3}$	$-2.5\times 10^{-1}$	$-3.7\times 10^{-3}$	$-1.5\times 10^{-3}$	$1.4\times 10^{0}$	$3.2\times 10^{-6}$	$-1.7\times 10^{2}$	0.16
C4h	$-1.3\times 10^{-3}$	$-9.0\times 10^{-2}$	$-2.2\times 10^{-3}$	$-1.4\times 10^{-3}$	$8.5\times 10^{-1}$	$1.8\times 10^{-6}$	$-1.0\times 10^{2}$	0.18
C6h	$-1.6\times 10^{-3}$	$-6.9\times 10^{-2}$	$-7.4\times 10^{-4}$	$-1.2\times 10^{-3}$	$3.1\times 10^{-1}$	$6.1\times 10^{-7}$	$-3.5\times 10^{1}$	0.18
T8h	$-2.5\times 10^{-3}$	$-9.5\times 10^{-2}$	$-1.2\times 10^{-3}$	$-1.2\times 10^{-3}$	$5.0\times 10^{-1}$	$9.9\times 10^{-7}$	$-6.1\times 10^{1}$	0.14
T10h	$-3.8\times 10^{-3}$	$-1.2\times 10^{-1}$	$-4.7\times 10^{-3}$	$-2.1\times 10^{-3}$	$1.8\times 10^{0}$	$4.0\times 10^{-6}$	$-2.3\times 10^{2}$	0.19
TP9h	$-5.8\times 10^{-4}$	$4.6\times 10^{-2}$	$-9.3\times 10^{-4}$	$-2.2\times 10^{-3}$	$4.1\times 10^{-1}$	$6.9\times 10^{-7}$	$-5.1\times 10^{1}$	0.20
TP7h	$-1.1\times 10^{-3}$	$-9.4\times 10^{-2}$	$1.4\times 10^{-3}$	$-1.7\times 10^{-3}$	$-5.0\times 10^{-1}$	$-1.2\times 10^{-6}$	$6.7\times 10^{1}$	0.27
CP5h	$-7.7\times 10^{-4}$	$-5.2\times 10^{-2}$	$1.6\times 10^{-4}$	$-1.5\times 10^{-3}$	$-3.2\times 10^{-2}$	$-2.0\times 10^{-7}$	$7.0\times 10^{0}$	0.21
CP3h	$1.3\times 10^{-4}$	$-2.4\times 10^{-2}$	$-4.4\times 10^{-5}$	$-1.7\times 10^{-3}$	$4.9\times 10^{-2}$	$-3.0\times 10^{-8}$	$-3.7\times 10^{0}$	0.30
CP1h	$7.1\times 10^{-4}$	$9.5\times 10^{-3}$	$-7.4\times 10^{-4}$	$-1.7\times 10^{-3}$	$3.2\times 10^{-1}$	$5.7\times 10^{-7}$	$-3.8\times 10^{1}$	0.30
CP2h	$2.1\times 10^{-3}$	$-7.8\times 10^{-2}$	$-1.2\times 10^{-3}$	$-1.9\times 10^{-3}$	$4.9\times 10^{-1}$	$1.0\times 10^{-6}$	$-5.7\times 10^{1}$	0.31
CP4h	$-5.2\times 10^{-5}$	$-4.5\times 10^{-2}$	$-5.6\times 10^{-4}$	$-1.7\times 10^{-3}$	$2.4\times 10^{-1}$	$4.4\times 10^{-7}$	$-2.7\times 10^{1}$	0.31
CP6h	$-1.7\times 10^{-3}$	$-6.1\times 10^{-2}$	$4.4\times 10^{-4}$	$-1.5\times 10^{-3}$	$-1.6\times 10^{-1}$	$-4.0\times 10^{-7}$	$2.5\times 10^{1}$	0.22
TP8h	$1.6\times 10^{-3}$	$-7.0\times 10^{-3}$	$4.7\times 10^{-4}$	$-1.6\times 10^{-3}$	$-1.7\times 10^{-1}$	$-4.4\times 10^{-7}$	$2.6\times 10^{1}$	0.23
TP10h	$-8.7\times 10^{-4}$	$-1.0\times 10^{-2}$	$-1.8\times 10^{-3}$	$-2.1\times 10^{-3}$	$7.0\times 10^{-1}$	$1.5\times 10^{-6}$	$-8.4\times 10^{1}$	0.26
P9h	$-9.6\times 10^{-4}$	$6.0\times 10^{-2}$	$-2.0\times 10^{-3}$	$-2.0\times 10^{-3}$	$8.1\times 10^{-1}$	$1.7\times 10^{-6}$	$-1.0\times 10^{2}$	0.39
P7h	$1.2\times 10^{-3}$	$-1.1\times 10^{-1}$	$1.7\times 10^{-3}$	$-2.0\times 10^{-3}$	$-6.7\times 10^{-1}$	$-1.4\times 10^{-6}$	$9.3\times 10^{1}$	0.40
P5h	$1.8\times 10^{-4}$	$-6.5\times 10^{-2}$	$1.5\times 10^{-4}$	$-1.7\times 10^{-3}$	$-2.8\times 10^{-2}$	$-2.0\times 10^{-7}$	$6.1\times 10^{0}$	0.28
P3h	$6.8\times 10^{-4}$	$-5.6\times 10^{-2}$	$-1.3\times 10^{-3}$	$-1.8\times 10^{-3}$	$5.1\times 10^{-1}$	$1.0\times 10^{-6}$	$-6.2\times 10^{1}$	0.34
P1h	$6.5\times 10^{-4}$	$-2.4\times 10^{-2}$	$-7.8\times 10^{-4}$	$-1.9\times 10^{-3}$	$3.4\times 10^{-1}$	$6.1\times 10^{-7}$	$-4.1\times 10^{1}$	0.34
P2h	$6.8\times 10^{-4}$	$-2.7\times 10^{-2}$	$-8.6\times 10^{-4}$	$-1.9\times 10^{-3}$	$3.7\times 10^{-1}$	$6.7\times 10^{-7}$	$-4.5\times 10^{1}$	0.33
P4h	$7.7\times 10^{-4}$	$-5.1\times 10^{-2}$	$-1.0\times 10^{-3}$	$-1.9\times 10^{-3}$	$4.1\times 10^{-1}$	$8.2\times 10^{-7}$	$-4.9\times 10^{1}$	0.32
P6h	$7.3\times 10^{-4}$	$-6.4\times 10^{-2}$	$-1.7\times 10^{-4}$	$-1.6\times 10^{-3}$	$7.7\times 10^{-2}$	$1.2\times 10^{-7}$	$-4.6\times 10^{0}$	0.27
P8h	$1.8\times 10^{-3}$	$-2.2\times 10^{-2}$	$1.5\times 10^{-3}$	$-1.6\times 10^{-3}$	$-5.9\times 10^{-1}$	$-1.3\times 10^{-6}$	$8.3\times 10^{1}$	0.34
P10h	$-1.9\times 10^{-4}$	$-7.9\times 10^{-2}$	$-1.6\times 10^{-3}$	$-2.1\times 10^{-3}$	$6.7\times 10^{-1}$	$1.3\times 10^{-6}$	$-8.3\times 10^{1}$	0.28
PO9h	$6.0\times 10^{-4}$	$9.1\times 10^{-3}$	$-3.0\times 10^{-3}$	$-2.1\times 10^{-3}$	$1.2\times 10^{0}$	$2.5\times 10^{-6}$	$-1.5\times 10^{2}$	0.32
PO7h	$-11.3\times 10^{-3}$	$-5.0\times 10^{-3}$	$3.7\times 10^{-4}$	$-2.1\times 10^{-3}$	$-1.4\times 10^{-1}$	$-3.1\times 10^{-7}$	$2.8\times 10^{1}$	0.39
PO5h	$-3.5\times 10^{-4}$	$-8.4\times 10^{-2}$	$-3.4\times 10^{-4}$	$-1.8\times 10^{-3}$	$1.5\times 10^{-1}$	$2.6\times 10^{-7}$	$-1.5\times 10^{1}$	0.36
PO3h	$8.5\times 10^{-4}$	$-6.5\times 10^{-2}$	$-1.6\times 10^{-3}$	$-1.8\times 10^{-3}$	$6.5\times 10^{-1}$	$1.4\times 10^{-6}$	$-7.9\times 10^{1}$	0.31
PO1h	$8.5\times 10^{-4}$	$-3.9\times 10^{-2}$	$-1.7\times 10^{-3}$	$-1.8\times 10^{-3}$	$6.9\times 10^{-1}$	$1.4\times 10^{-6}$	$-8.4\times 10^{1}$	0.37
PO2h	$1.0\times 10^{-3}$	$-4.0\times 10^{-2}$	$-9.1\times 10^{-4}$	$-1.8\times 10^{-3}$	$3.8\times 10^{-1}$	$7.3\times 10^{-7}$	$-4.5\times 10^{1}$	0.36
PO4h	$6.6\times 10^{-4}$	$-7.8\times 10^{-2}$	$-1.4\times 10^{-3}$	$-1.8\times 10^{-3}$	$5.8\times 10^{-1}$	$1.2\times 10^{-6}$	$-7.1\times 10^{1}$	0.35
PO6h	$2.3\times 10^{-4}$	$-9.2\times 10^{-2}$	$-4.9\times 10^{-4}$	$-1.8\times 10^{-3}$	$2.0\times 10^{-1}$	$4.0\times 10^{-7}$	$-2.1\times 10^{1}$	0.39
PO8h	$7.8\times 10^{-4}$	$-8.9\times 10^{-2}$	$1.4\times 10^{-3}$	$-2.1\times 10^{-3}$	$-5.6\times 10^{-1}$	$-1.2\times 10^{-6}$	$8.0\times 10^{1}$	0.55
PO10h	$7.2\times 10^{-4}$	$-7.3\times 10^{-2}$	$-3.2\times 10^{-3}$	$-2.2\times 10^{-3}$	$1.3\times 10^{0}$	$2.8\times 10^{-6}$	$-1.6\times 10^{2}$	0.36
O1h	$-9.1\times 10^{-4}$	$-1.0\times 10^{-1}$	$4.7\times 10^{-5}$	$-2.2\times 10^{-3}$	$-2.0\times 10^{-2}$	$-2.6\times 10^{-8}$	$1.0\times 10^{1}$	0.39
O2h	$2.4\times 10^{-3}$	$2.7\times 10^{-2}$	$7.5\times 10^{-4}$	$-2.1\times 10^{-3}$	$-2.9\times 10^{-1}$	$-6.3\times 10^{-7}$	$4.4\times 10^{1}$	0.35
I1h	$1.6\times 10^{-3}$	$-8.1\times 10^{-2}$	$-3.6\times 10^{-3}$	$-2.0\times 10^{-3}$	$1.4\times 10^{0}$	$3.0\times 10^{-6}$	$-1.8\times 10^{2}$	0.86
I2h	$8.2\times 10^{-4}$	$-8.9\times 10^{-2}$	$-2.2\times 10^{-3}$	$-1.9\times 10^{-3}$	$9.0\times 10^{-1}$	$1.9\times 10^{-6}$	$-1.1\times 10^{2}$	0.48
AFp9	$4.2\times 10^{-3}$	$-5.6\times 10^{-2}$	$-3.6\times 10^{-3}$	$-2.3\times 10^{-3}$	$1.4\times 10^{0}$	$3.1\times 10^{-6}$	$-1.7\times 10^{2}$	0.48
AFp7	$5.1\times 10^{-3}$	$-1.4\times 10^{-1}$	$-2.1\times 10^{-3}$	$-2.3\times 10^{-3}$	$7.8\times 10^{-1}$	$1.8\times 10^{-6}$	$-1.0\times 10^{2}$	0.19
AFp5	$2.5\times 10^{-4}$	$-8.4\times 10^{-2}$	$1.1\times 10^{-4}$	$-1.2\times 10^{-3}$	$-1.8\times 10^{-2}$	$-1.4\times 10^{-7}$	$5.5\times 10^{0}$	0.19
AFp3	$5.8\times 10^{-4}$	$-8.4\times 10^{-2}$	$3.7\times 10^{-4}$	$-1.1\times 10^{-3}$	$-1.3\times 10^{-1}$	$-3.5\times 10^{-7}$	$2.0\times 10^{1}$	0.18
AFp1	$1.9\times 10^{-3}$	$-1.2\times 10^{-1}$	$2.5\times 10^{-4}$	$-1.3\times 10^{-3}$	$-9.8\times 10^{-2}$	$-2.2\times 10^{-7}$	$1.9\times 10^{1}$	0.16
AFpz	$1.8\times 10^{-3}$	$-1.1\times 10^{-1}$	$-9.7\times 10^{-5}$	$-1.5\times 10^{-3}$	$3.8\times 10^{-2}$	$8.4\times 10^{-8}$	$1.7\times 10^{0}$	0.18
AFp2	$1.4\times 10^{-3}$	$-1.1\times 10^{-1}$	$2.1\times 10^{-4}$	$-1.4\times 10^{-3}$	$-8.6\times 10^{-2}$	$-1.8\times 10^{-7}$	$1.8\times 10^{1}$	0.18
AFp4	$3.8\times 10^{-4}$	$-8.1\times 10^{-2}$	$4.3\times 10^{-4}$	$-1.4\times 10^{-3}$	$-1.6\times 10^{-1}$	$-3.9\times 10^{-7}$	$2.5\times 10^{1}$	0.20
AFp6	$6.8\times 10^{-4}$	$-6.8\times 10^{-2}$	$5.5\times 10^{-4}$	$-1.4\times 10^{-3}$	$-2.2\times 10^{-1}$	$-4.7\times 10^{-7}$	$3.5\times 10^{1}$	0.21
AFp8	$2.5\times 10^{-3}$	$1.4\times 10^{-2}$	$-4.2\times 10^{-4}$	$-2.3\times 10^{-3}$	$1.8\times 10^{-1}$	$3.3\times 10^{-7}$	$-1.6\times 10^{1}$	0.17
AFp10	$3.4\times 10^{-3}$	$-5.0\times 10^{-2}$	$-2.2\times 10^{-3}$	$-2.3\times 10^{-3}$	$8.8\times 10^{-1}$	$1.8\times 10^{-6}$	$-1.1\times 10^{2}$	0.36
AFF9	$3.9\times 10^{-3}$	$-1.8\times 10^{-2}$	$-3.5\times 10^{-3}$	$-2.6\times 10^{-3}$	$1.4\times 10^{0}$	$2.9\times 10^{-6}$	$-1.8\times 10^{2}$	0.73
AFF7	$-7.8\times 10^{-4}$	$-6.5\times 10^{-2}$	$-2.8\times 10^{-3}$	$-1.6\times 10^{-3}$	$1.1\times 10^{0}$	$2.3\times 10^{-6}$	$-1.4\times 10^{2}$	0.20
AFF5	$8.3\times 10^{-4}$	$-1.0\times 10^{-1}$	$-6.9\times 10^{-5}$	$-8.4\times 10^{-4}$	$4.4\times 10^{-2}$	$3.0\times 10^{-8}$	$-8.4\times 10^{-1}$	0.18
AFF3	$8.1\times 10^{-4}$	$-1.0\times 10^{-1}$	$-1.4\times 10^{-4}$	$-1.1\times 10^{-3}$	$6.4\times 10^{-2}$	$1.0\times 10^{-7}$	$-2.7\times 10^{0}$	0.15
AFF1	$6.4\times 10^{-4}$	$-1.1\times 10^{-1}$	$-6.6\times 10^{-5}$	$-1.1\times 10^{-3}$	$2.5\times 10^{-2}$	$5.6\times 10^{-8}$	$4.0\times 10^{0}$	0.15
AFFz	$1.5\times 10^{-3}$	$-1.4\times 10^{-1}$	$-1.0\times 10^{-3}$	$-1.3\times 10^{-3}$	$4.1\times 10^{-1}$	$8.7\times 10^{-7}$	$-4.6\times 10^{1}$	0.18
AFF2	$1.2\times 10^{-3}$	$-1.3\times 10^{-1}$	$-9.1\times 10^{-4}$	$-1.2\times 10^{-3}$	$3.7\times 10^{-1}$	$7.4\times 10^{-7}$	$-4.3\times 10^{1}$	0.19
AFF4	$3.0\times 10^{-4}$	$-1.1\times 10^{-1}$	$-9.0\times 10^{-4}$	$-1.1\times 10^{-3}$	$3.6\times 10^{-1}$	$7.5\times 10^{-7}$	$-4.1\times 10^{1}$	0.18
AFF6	$-1.3\times 10^{-3}$	$-1.5\times 10^{-1}$	$-1.3\times 10^{-3}$	$-9.9\times 10^{-4}$	$4.9\times 10^{-1}$	$1.1\times 10^{-6}$	$-5.7\times 10^{1}$	0.11
AFF8	$6.2\times 10^{-4}$	$-1.7\times 10^{-1}$	$-8.4\times 10^{-4}$	$-1.5\times 10^{-3}$	$3.4\times 10^{-1}$	$7.1\times 10^{-7}$	$-3.8\times 10^{1}$	0.24
AFF10	$2.8\times 10^{-3}$	$-6.9\times 10^{-2}$	$-3.3\times 10^{-3}$	$-2.8\times 10^{-3}$	$1.3\times 10^{0}$	$2.8\times 10^{-6}$	$-1.6\times 10^{2}$	0.70
FFT9	$1.8\times 10^{-3}$	$2.1\times 10^{-3}$	$-1.5\times 10^{-3}$	$-2.7\times 10^{-3}$	$5.9\times 10^{-1}$	$1.2\times 10^{-6}$	$-7.3\times 10^{1}$	0.83
FFT7	$-1.4\times 10^{-3}$	$3.0\times 10^{-2}$	$1.7\times 10^{-3}$	$-1.7\times 10^{-3}$	$-6.3\times 10^{-1}$	$-1.5\times 10^{-6}$	$8.4\times 10^{1}$	0.33
FFC5	$-1.4\times 10^{-3}$	$-3.9\times 10^{-2}$	$2.1\times 10^{-4}$	$-9.3\times 10^{-4}$	$-8.3\times 10^{-2}$	$-1.7\times 10^{-7}$	$1.7\times 10^{1}$	0.07
FFC3	$3.5\times 10^{-4}$	$-1.1\times 10^{-1}$	$-6.7\times 10^{-4}$	$-1.1\times 10^{-3}$	$2.6\times 10^{-1}$	$5.8\times 10^{-7}$	$-2.5\times 10^{1}$	0.11
FFC1	$1.5\times 10^{-3}$	$-1.2\times 10^{-1}$	$-1.0\times 10^{-3}$	$-1.1\times 10^{-3}$	$3.8\times 10^{-1}$	$9.1\times 10^{-7}$	$-3.9\times 10^{1}$	0.14
FFCz	$7.7\times 10^{-4}$	$-1.1\times 10^{-1}$	$-1.8\times 10^{-3}$	$-1.2\times 10^{-3}$	$6.9\times 10^{-1}$	$1.6\times 10^{-6}$	$-8.2\times 10^{1}$	0.21
FFC2	$9.5\times 10^{-4}$	$-1.0\times 10^{-1}$	$-3.0\times 10^{-3}$	$-1.2\times 10^{-3}$	$1.2\times 10^{0}$	$2.5\times 10^{-6}$	$-1.5\times 10^{2}$	0.25
FFC4	$4.2\times 10^{-4}$	$-7.6\times 10^{-2}$	$-1.2\times 10^{-3}$	$-1.0\times 10^{-3}$	$5.1\times 10^{-1}$	$1.0\times 10^{-6}$	$-6.2\times 10^{1}$	0.18
FFC6	$-1.2\times 10^{-3}$	$-8.4\times 10^{-2}$	$-6.3\times 10^{-4}$	$-9.4\times 10^{-4}$	$2.7\times 10^{-1}$	$5.1\times 10^{-7}$	$-3.0\times 10^{1}$	0.14
FFT8	$2.3\times 10^{-3}$	$7.7\times 10^{-2}$	$-1.7\times 10^{-3}$	$-1.7\times 10^{-3}$	$6.8\times 10^{-1}$	$1.4\times 10^{-6}$	$-8.6\times 10^{1}$	0.11
FFT10	$1.9\times 10^{-3}$	$1.7\times 10^{-2}$	$-4.2\times 10^{-3}$	$-2.7\times 10^{-3}$	$1.7\times 10^{0}$	$3.5\times 10^{-6}$	$-2.1\times 10^{2}$	0.93
FTT9	$-2.6\times 10^{-4}$	$3.2\times 10^{-2}$	$-1.5\times 10^{-3}$	$-2.1\times 10^{-3}$	$6.1\times 10^{-1}$	$1.3\times 10^{-6}$	$-7.2\times 10^{1}$	0.71
FTT7	$1.3\times 10^{-3}$	$-8.3\times 10^{-2}$	$-1.5\times 10^{-3}$	$-2.2\times 10^{-3}$	$6.0\times 10^{-1}$	$1.3\times 10^{-6}$	$-7.1\times 10^{1}$	0.16
FCC5	$-5.4\times 10^{-4}$	$-5.3\times 10^{-2}$	$5.2\times 10^{-4}$	$-1.1\times 10^{-3}$	$-2.1\times 10^{-1}$	$-4.4\times 10^{-7}$	$3.4\times 10^{1}$	0.09
FCC3	$-9.8\times 10^{-4}$	$-7.1\times 10^{-2}$	$-4.0\times 10^{-5}$	$-1.2\times 10^{-3}$	$2.6\times 10^{-2}$	$2.0\times 10^{-8}$	$2.4\times 10^{0}$	0.14
FCC1	$1.7\times 10^{-3}$	$-8.5\times 10^{-2}$	$-1.3\times 10^{-3}$	$-1.4\times 10^{-3}$	$5.3\times 10^{-1}$	$1.1\times 10^{-6}$	$-6.4\times 10^{1}$	0.21
FCCz	$1.2\times 10^{-3}$	$-2.3\times 10^{-1}$	$-3.4\times 10^{-3}$	$-1.5\times 10^{-3}$	$1.3\times 10^{0}$	$2.9\times 10^{-6}$	$-1.6\times 10^{2}$	0.20
FCC2	$-4.9\times 10^{-4}$	$-1.4\times 10^{-1}$	$-4.3\times 10^{-3}$	$-1.4\times 10^{-3}$	$1.7\times 10^{0}$	$3.7\times 10^{-6}$	$-2.1\times 10^{2}$	0.19
FCC4	$-1.1\times 10^{-3}$	$-9.2\times 10^{-2}$	$-2.1\times 10^{-3}$	$-1.2\times 10^{-3}$	$8.5\times 10^{-1}$	$1.8\times 10^{-6}$	$-1.0\times 10^{2}$	0.13
FCC6	$-1.9\times 10^{-3}$	$-7.4\times 10^{-2}$	$-1.3\times 10^{-3}$	$-1.0\times 10^{-3}$	$5.3\times 10^{-1}$	$1.1\times 10^{-6}$	$-6.5\times 10^{1}$	0.12
FTT8	$-3.1\times 10^{-3}$	$-1.7\times 10^{-2}$	$-1.5\times 10^{-3}$	$-1.5\times 10^{-3}$	$6.1\times 10^{-1}$	$1.2\times 10^{-6}$	$-7.8\times 10^{1}$	0.14
FTT10	$3.4\times 10^{-3}$	$6.2\times 10^{-2}$	$-3.2\times 10^{-3}$	$-1.9\times 10^{-3}$	$1.3\times 10^{0}$	$2.6\times 10^{-6}$	$-1.7\times 10^{2}$	0.33
TTP9	$1.7\times 10^{-3}$	$-5.1\times 10^{-2}$	$-1.1\times 10^{-3}$	$-1.9\times 10^{-3}$	$4.0\times 10^{-1}$	$9.4\times 10^{-7}$	$-4.4\times 10^{1}$	0.42
TTP7	$-1.4\times 10^{-3}$	$-4.0\times 10^{-2}$	$1.2\times 10^{-3}$	$-2.2\times 10^{-3}$	$-4.3\times 10^{-1}$	$-1.1\times 10^{-6}$	$5.9\times 10^{1}$	0.28
CCP5	$-7.9\times 10^{-4}$	$-7.9\times 10^{-2}$	$3.5\times 10^{-4}$	$-1.3\times 10^{-3}$	$-1.1\times 10^{-1}$	$-3.3\times 10^{-7}$	$1.9\times 10^{1}$	0.18
CCP3	$-4.5\times 10^{-4}$	$-3.5\times 10^{-2}$	$-3.8\times 10^{-4}$	$-1.5\times 10^{-3}$	$1.7\times 10^{-1}$	$2.8\times 10^{-7}$	$-1.8\times 10^{1}$	0.24
CCP1	$6.9\times 10^{-4}$	$9.1\times 10^{-3}$	$-7.7\times 10^{-4}$	$-1.6\times 10^{-3}$	$3.2\times 10^{-1}$	$6.1\times 10^{-7}$	$-3.8\times 10^{1}$	0.25
CCPz	$2.3\times 10^{-3}$	$-8.2\times 10^{-2}$	$-1.4\times 10^{-3}$	$-1.7\times 10^{-3}$	$5.4\times 10^{-1}$	$1.2\times 10^{-6}$	$-6.2\times 10^{1}$	0.24
CCP2	$2.3\times 10^{-3}$	$-1.3\times 10^{-1}$	$-2.0\times 10^{-3}$	$-1.6\times 10^{-3}$	$7.7\times 10^{-1}$	$1.7\times 10^{-6}$	$-9.1\times 10^{1}$	0.21
CCP4	$-1.5\times 10^{-3}$	$-6.6\times 10^{-2}$	$-6.3\times 10^{-4}$	$-1.4\times 10^{-3}$	$2.6\times 10^{-1}$	$5.1\times 10^{-7}$	$-2.9\times 10^{1}$	0.23
CCP6	$-1.5\times 10^{-3}$	$-3.9\times 10^{-2}$	$-1.0\times 10^{-5}$	$-1.2\times 10^{-3}$	$2.8\times 10^{-2}$	$-2.2\times 10^{-8}$	$1.8\times 10^{-1}$	0.16
TTP8	$-2.8\times 10^{-4}$	$-1.1\times 10^{-1}$	$5.0\times 10^{-4}$	$-2.0\times 10^{-3}$	$-1.7\times 10^{-1}$	$-4.6\times 10^{-7}$	$2.6\times 10^{1}$	0.19
TTP10	$2.1\times 10^{-3}$	$-7.2\times 10^{-2}$	$-1.8\times 10^{-3}$	$-1.7\times 10^{-3}$	$7.3\times 10^{-1}$	$1.5\times 10^{-6}$	$-9.0\times 10^{1}$	0.13
TPP9	$-1.0\times 10^{-3}$	$-1.5\times 10^{-2}$	$-9.9\times 10^{-4}$	$-1.6\times 10^{-3}$	$4.1\times 10^{-1}$	$7.8\times 10^{-7}$	$-5.1\times 10^{1}$	0.78
TPP7	$-8.6\times 10^{-5}$	$-4.0\times 10^{-2}$	$-9.1\times 10^{-5}$	$-2.0\times 10^{-3}$	$6.2\times 10^{-2}$	$5.9\times 10^{-8}$	$-3.0\times 10^{0}$	0.29
CPP5	$3.3\times 10^{-4}$	$-9.8\times 10^{-2}$	$5.8\times 10^{-4}$	$-1.6\times 10^{-3}$	$-2.0\times 10^{-1}$	$-5.4\times 10^{-7}$	$3.0\times 10^{1}$	0.31
CPP3	$-4.5\times 10^{-5}$	$-5.4\times 10^{-2}$	$7.0\times 10^{-7}$	$-1.7\times 10^{-3}$	$3.2\times 10^{-2}$	$-66.8\times 10^{-8}$	$-1.6\times 10^{0}$	0.31
CPP1	$8.0\times 10^{-4}$	$-1.9\times 10^{-2}$	$-8.1\times 10^{-4}$	$-1.8\times 10^{-3}$	$3.4\times 10^{-1}$	$6.3\times 10^{-7}$	$-4.2\times 10^{1}$	0.34
CPPz	$5.5\times 10^{-4}$	$-1.4\times 10^{-2}$	$-6.2\times 10^{-4}$	$-1.9\times 10^{-3}$	$2.7\times 10^{-1}$	$4.6\times 10^{-7}$	$-3.2\times 10^{1}$	0.33
CPP2	$7.3\times 10^{-4}$	$-3.8\times 10^{-2}$	$-1.2\times 10^{-3}$	$-1.9\times 10^{-3}$	$4.8\times 10^{-1}$	$9.6\times 10^{-7}$	$-5.9\times 10^{1}$	0.33
CPP4	$-8.1\times 10^{-5}$	$-5.7\times 10^{-2}$	$1.7\times 10^{-4}$	$-1.7\times 10^{-3}$	$-4.9\times 10^{-2}$	$-1.8\times 10^{-7}$	$1.1\times 10^{1}$	0.29
CPP6	$-9.7\times 10^{-4}$	$-7.5\times 10^{-2}$	$1.1\times 10^{-3}$	$-1.5\times 10^{-3}$	$-4.2\times 10^{-1}$	$-9.5\times 10^{-7}$	$6.0\times 10^{1}$	0.21
TPP8	$4.0\times 10^{-4}$	$-2.8\times 10^{-3}$	$7.2\times 10^{-4}$	$-2.0\times 10^{-3}$	$-2.7\times 10^{-1}$	$-6.3\times 10^{-7}$	$4.0\times 10^{1}$	0.27
TPP10	$-1.1\times 10^{-4}$	$1.8\times 10^{-1}$	$5.3\times 10^{-4}$	$-1.3\times 10^{-3}$	$-1.5\times 10^{-1}$	$-5.9\times 10^{-7}$	$1.9\times 10^{1}$	0.83
PPO9	$8.4\times 10^{-4}$	$-5.9\times 10^{-2}$	$-1.2\times 10^{-3}$	$-1.7\times 10^{-3}$	$4.8\times 10^{-1}$	$9.3\times 10^{-7}$	$-6.0\times 10^{1}$	0.80
PPO7	$4.0\times 10^{-4}$	$-2.8\times 10^{-2}$	$-1.4\times 10^{-3}$	$-2.4\times 10^{-3}$	$5.5\times 10^{-1}$	$1.2\times 10^{-6}$	$-6.2\times 10^{1}$	0.44
PPO5	$-2.5\times 10^{-4}$	$-5.8\times 10^{-2}$	$1.2\times 10^{-4}$	$-1.8\times 10^{-3}$	$-1.9\times 10^{-2}$	$-1.5\times 10^{-7}$	$5.7\times 10^{0}$	0.31
PPO3	$2.8\times 10^{-4}$	$-7.9\times 10^{-2}$	$-1.1\times 10^{-3}$	$-1.7\times 10^{-3}$	$4.6\times 10^{-1}$	$9.1\times 10^{-7}$	$-5.5\times 10^{1}$	0.29
PPO1	$9.0\times 10^{-4}$	$-4.3\times 10^{-2}$	$-1.9\times 10^{-3}$	$-1.8\times 10^{-3}$	$7.6\times 10^{-1}$	$1.6\times 10^{-6}$	$-9.4\times 10^{1}$	0.33
PPOz	$5.5\times 10^{-4}$	$-2.3\times 10^{-2}$	$-1.3\times 10^{-3}$	$-1.9\times 10^{-3}$	$5.2\times 10^{-1}$	$1.0\times 10^{-6}$	$-6.5\times 10^{1}$	0.33
PPO2	$8.2\times 10^{-4}$	$-5.7\times 10^{-2}$	$-1.1\times 10^{-3}$	$-1.9\times 10^{-3}$	$4.7\times 10^{-1}$	$9.1\times 10^{-7}$	$-5.7\times 10^{1}$	0.33
PPO4	$6.6\times 10^{-4}$	$-6.8\times 10^{-2}$	$-7.9\times 10^{-4}$	$-1.8\times 10^{-3}$	$3.3\times 10^{-1}$	$6.4\times 10^{-7}$	$-3.7\times 10^{1}$	0.31
PPO6	$1.1\times 10^{-3}$	$-8.9\times 10^{-2}$	$8.0\times 10^{-5}$	$-1.7\times 10^{-3}$	$-2.4\times 10^{-2}$	$-8.2\times 10^{-8}$	$8.9\times 10^{0}$	0.36
PPO8	$-8.0\times 10^{-4}$	$-8.5\times 10^{-3}$	$3.9\times 10^{-4}$	$-2.2\times 10^{-3}$	$-1.7\times 10^{-1}$	$-2.9\times 10^{-7}$	$3.1\times 10^{1}$	0.36
PPO10	$6.6\times 10^{-4}$	$-3.8\times 10^{-2}$	$8.0\times 10^{-4}$	$-1.9\times 10^{-3}$	$-2.9\times 10^{-1}$	$-7.8\times 10^{-7}$	$4.1\times 10^{1}$	0.47
POO9	$2.1\times 10^{-3}$	$-5.8\times 10^{-2}$	$-3.4\times 10^{-3}$	$-1.9\times 10^{-3}$	$1.3\times 10^{0}$	$2.8\times 10^{-6}$	$-1.7\times 10^{2}$	0.93
POO7	$-2.0\times 10^{-4}$	$1.1\times 10^{-2}$	$-4.7\times 10^{-4}$	$-2.3\times 10^{-3}$	$1.8\times 10^{-1}$	$4.0\times 10^{-7}$	$-1.4\times 10^{1}$	0.40
POO5	$4.9\times 10^{-4}$	$-4.5\times 10^{-2}$	$6.2\times 10^{-4}$	$-2.0\times 10^{-3}$	$-2.5\times 10^{-1}$	$-5.3\times 10^{-7}$	$3.8\times 10^{1}$	0.35
POO3	$-3.1\times 10^{-4}$	$-7.4\times 10^{-2}$	$-7.4\times 10^{-4}$	$-1.9\times 10^{-3}$	$3.0\times 10^{-1}$	$6.2\times 10^{-7}$	$-3.3\times 10^{1}$	0.40
POO1	$5.4\times 10^{-4}$	$-9.4\times 10^{-2}$	$-1.1\times 10^{-3}$	$-1.9\times 10^{-3}$	$4.5\times 10^{-1}$	$9.3\times 10^{-7}$	$-5.2\times 10^{1}$	0.37
POOz	$1.1\times 10^{-3}$	$-6.2\times 10^{-2}$	$-1.0\times 10^{-3}$	$-1.8\times 10^{-3}$	$4.2\times 10^{-1}$	$8.6\times 10^{-7}$	$-4.9\times 10^{1}$	0.46
POO2	$1.4\times 10^{-3}$	$-7.8\times 10^{-2}$	$-1.0\times 10^{-3}$	$-1.8\times 10^{-3}$	$4.2\times 10^{-1}$	$8.3\times 10^{-7}$	$-5.0\times 10^{1}$	0.37
POO4	$7.5\times 10^{-4}$	$-9.2\times 10^{-2}$	$-3.0\times 10^{-4}$	$-1.8\times 10^{-3}$	$1.3\times 10^{-1}$	$2.2\times 10^{-7}$	$-1.3\times 10^{1}$	0.37
POO6	$-1.1\times 10^{-3}$	$-6.7\times 10^{-2}$	$2.9\times 10^{-4}$	$-1.9\times 10^{-3}$	$-1.3\times 10^{-1}$	$-2.0\times 10^{-7}$	$2.6\times 10^{1}$	0.46
POO8	$-1.2\times 10^{-3}$	$-1.9\times 10^{-2}$	$-1.5\times 10^{-3}$	$-2.4\times 10^{-3}$	$5.5\times 10^{-1}$	$1.3\times 10^{-6}$	$-6.1\times 10^{1}$	0.33
POO10	$1.5\times 10^{-3}$	$-6.7\times 10^{-2}$	$-2.6\times 10^{-3}$	$-2.1\times 10^{-3}$	$1.0\times 10^{0}$	$2.2\times 10^{-6}$	$-1.3\times 10^{2}$	0.78
OI1	$-3.8\times 10^{-4}$	$-2.1\times 10^{-2}$	$-2.3\times 10^{-3}$	$-2.2\times 10^{-3}$	$9.2\times 10^{-1}$	$1.9\times 10^{-6}$	$-1.2\times 10^{2}$	0.39
OIz	$1.3\times 10^{-4}$	$-8.5\times 10^{-2}$	$-4.0\times 10^{-3}$	$-2.1\times 10^{-3}$	$1.5\times 10^{0}$	$3.4\times 10^{-6}$	$-1.9\times 10^{2}$	0.39
OI2	$3.4\times 10^{-4}$	$-2.8\times 10^{-2}$	$-4.1\times 10^{-3}$	$-2.2\times 10^{-3}$	$1.6\times 10^{0}$	$3.5\times 10^{-6}$	$-2.0\times 10^{2}$	0.38

## Appendix A: Generalized Model of the Differential Pathlength Factor

The earlier works by Scholkmann and Wolf^37^ and Duncan et al.^31^ have suggested equations to estimate the DPF as a function of the subject age. Using our simulations from the N=90 subjects in this work, we attempted to fit a similar equation to the work of Scholkmann and Wolf to additionally incorporate head circumference and gender. The DPF values from each of the 320 10-5 positions on the head by 90 subjects were fit to a linear regression model using up to a third power of head circumference, age, and wavelength. A stepwise regression of the entire dataset was used to find the best set of covariates given the inclusion criteria of p<0.05 for the change in F-statistic of the increased model. Based on this, we model with a total of seven coefficients given by

$$\mathrm{DPF}=\beta_{0}+\beta_{1}(\mathrm{circ})+\beta_{2}{\left(\mathrm{circ}\right)}^{2}+\beta_{3}{\left(\mathrm{circ}\right)}^{3}+\beta_{4}\{\begin{array}{c} 1\;\text{if}\;\text{female}\\ 0 \end{array}\}+\beta_{5}\cdot (\text{age})+\beta_{6}\cdot (\lambda ),$$

where circ is the head circumference in millimeters, age is in months, and the wavelength is in nanometeres units.
